# Rye Snow Mold-Associated *Microdochium nivale* Strains Inhabiting a Common Area: Variability in Genetics, Morphotype, Extracellular Enzymatic Activities, and Virulence

**DOI:** 10.3390/jof6040335

**Published:** 2020-12-03

**Authors:** Vladimir Gorshkov, Elena Osipova, Mira Ponomareva, Sergey Ponomarev, Natalia Gogoleva, Olga Petrova, Olga Gogoleva, Azat Meshcherov, Alexander Balkin, Elena Vetchinkina, Kim Potapov, Yuri Gogolev, Viktor Korzun

**Affiliations:** 1 Laboratory of Plant Infectious Diseases, FRC Kazan Scientific Center of RAS, ul. Lobachevskogo, 2/31, 420111 Kazan, Russia; eva-0@mail.ru (E.O.); smponomarev@yandex.ru (M.P.); S.Ponomarev2020@yandex.ru (S.P.); negogoleva@gmail.com (N.G.); poe60@mail.ru (O.P.); gogolewaoa@yandex.ru (O.G.); strosaz125@gmail.com (A.M.); balkinas@yandex.ru (A.B.); potapov_ko@mail.ru (K.P.); gogolev.yuri@gmail.com (Y.G.); viktor.korzun@kws.com (V.K.); 2 Institute of Biochemistry and Physiology of Plants and Microorganisms, Russian Academy of Sciences (IBPPM RAS), 13 Prospekt Entuziastov, 410049 Saratov, Russia; elenavetrus@yandex.ru; 3 KWS SAAT SE & Co. KGaA, Grimsehlstr. 31, 37555 Einbeck, Germany

**Keywords:** plant–microbe interactions, *Microdochium nivale*, snow mold, plant cell-wall-degrading enzymes, virulence

## Abstract

Snow mold is a severe plant disease caused by psychrophilic or psychrotolerant fungi, of which *Microdochium* species are the most harmful. A clear understanding of *Microdochium* biology has many gaps; the pathocomplex and its dynamic are poorly characterized, virulence factors are unknown, genome sequences are not available, and the criteria of plant snow mold resistance are not elucidated. Our study aimed to identify comprehensive characteristics of a local community of snow mold-causing *Microdochium* species colonizing a particular crop culture. By using the next-generation sequencing (NGS) technique, we characterized fungal and bacterial communities of pink snow mold-affected winter rye (*Secale cereale*) plants within a given geographical location shortly after snowmelt. Twenty-one strains of *M. nivale* were isolated, classified on the basis of internal transcribed spacer 2 (ITS2) region, and characterized by morphology, synthesis of extracellular enzymes, and virulence. Several types of extracellular enzymatic activities, the level of which had no correlations with the degree of virulence, were revealed for *Microdochium* species for the first time. Our study shows that genetically and phenotypically diverse *M. nivale* strains simultaneously colonize winter rye plants within a common area, and each strain is likely to utilize its own, unique strategy to cause the disease using “a personal” pattern of extracellular enzymes.

## 1. Introduction

Snow mold is a severe plant disease caused by psychrophilic or psychrotolerant fungi and oomycetes under snow cover at or shortly below the freezing point [1,2]. The presence of prolonged snow cover reduces competition with other (non-psychrotolerant) pathogens, weakens plant immunity, and provides insulation, darkness, and humidity, creating favorable conditions for the snow mold-causing pathogens [3,4,5,6]. Snow mold is distributed worldwide (Canada, United States (US), United Kingdom (UK), Europe, Japan), predominantly in the Northern Hemisphere [7,8,9,10]. In the central European part of Russia (Tatarstan Republic), a regular annual occurrence of snow mold in winter rye for the period 2001–2019 was observed at nearly or above the epiphytotic level [11,12]. In the Kirov region of Russia, the snow mold spread in winter ranged from 29% to 100% of total growing area with a total loss of the cereal crops in nine out of 20 years [13,14]. From 2017 to 2019, snow mold affected an area of 445,600 to 856,500 hectares in Russia [15]. In Canada, the prevention of snow mold disease accounts for almost 50% of the yearly fungicide use on turf grass [3]. The resistance of the most important cereal cultivars to these pathogens is currently low [12]. Therefore, snow mold is an important problem for breeding and managing winter cereals (rye, wheat, oat, barley, triticale), as well as forage and turf grasses [16].

There are several types of snow mold, each caused by different taxa: pink snow mold (*Microdochium nivale*, *M. majus*), gray or speckled snow mold (*Typhula idahoensis*, *T. ishikariensis*, *T. incarnata*), snow scald (*Myriosclerotinia borealis*), and snow rot (*Pythium iwayami* and *P. okanoganense*) [5]. *Microdochium nivale* (Fr.) Samuels & Hallett and *M. majus* (Wollenw.) Glynn & S.G. Edwards are the most harmful species among the snow mold-causing pathogens. *M. nivale* and *M. majus* were long considered as varieties of a single species (*M. nivale* var. *nivale* and *M. nivale* var. *majus*). These two varieties were distinguished mostly on the basis of conidial morphology [17]. Furthermore, according to a comparison of the elongation factor 1-alpha gene, these two varieties were reclassified into separate species *M. nivale* and *M. majus* [18]. Nevertheless, since many studies on closely related *M. nivale* and *M. majus* were performed before they were recognized as distinct species, they collectively continue to be referred to as *M. nivale sensu lato*, and, when the variety is specified, it is referred to as *M. nivale sensu stricto* and *M. majus* [19].

During winter, when most of the phytopathogens are not active, psychrotolerant *M. nivale sensu lato* can grow at temperatures as low as −5 °C which is conferred by special adaptive mechanisms related to alterations in the fatty-acid composition [20]. These fungi monopolize plant resources by proliferating in the under-snow habitat where antagonists are practically absent. Pink snow mold caused by *M. nivale sensu lato* is associated with leaf and stem desiccation coupled with the extensive growth of white or pink mycelium and formation of orange sporodochia, which is expressed in bleached to orange-brown patches of matted leaf tissue [21]. In addition, these fungi lead to the reduction of seed germination, as well as pre- and post-emergence death of seedlings [22,23,24]. Being a facultative snow mold pathogen, *M. nivale sensu lato* may thrive even before the snow cover or after the snow melting if prolonged periods of high humidity and low positive temperatures are maintained [10,21,25]. These phytopathogens can damage plants throughout the entire growing season causing seedling blight, foot rot, leaf blight, and head blight [26,27,28,29,30,31,32,33,34,35]. Thus, *M. nivale sensu lato*-caused diseases are not restricted only to regions with prolonged snow cover, as was recently thought. *M. nivale sensu lato* may combine biotrophic, necrotrophic, and saprotrophic lifestyles depending on a number of factors; therefore, its presence in a phytocenosis does not necessary lead to plant damage. These fungi do not produce mycotoxins and, thus, do not adversely affect grain quality, but may cause a significant yield reduction [27,29,36,37,38,39,40,41].

*M. nivale* initially infects leaf sheaths and leaf blades growing in contact with infected soil and invades the plant interior via stomata. Hyphae then penetrate the crown cortex and vascular bundles spreading systemically through the plant. *M. nivale* was shown to form vesicle-like structures resembling haustoria inside the plant cells [42,43]. After penetrating the mesophyll, hyphae may grow outward and protrude through the stomata. The virulence of different isolates of *M. nivale sensu stricto* and *M. majus* differs significantly [41,44,45,46]. However, the genetic and physiological bases for the differences in virulence remain to be determined.

Although *M. nivale sensu stricto* and *M. majus* colonize several plant species, each of these species was shown to have preferences in host plant [47,48,49,50,51,52,53]. On turf grasses, *M. nivale sensu stricto* was common [48,50,53], while *M. majus* was almost restricted to cereals. *M. nivale sensu stricto* is more frequently found on rye, while *M. majus* prefers wheat, oat, barley, and triticale [43,49,54,55,56]. *M. majus* showed a selective advantage on winter wheat and winter oat seedlings and *M. nivale sensu stricto* showed a strong selective advantage on winter rye seedlings in a mixed inoculation trial [49]. Irrespective of their original host, *M. nivale sensu stricto* and *M. majus* display differential virulence toward their hosts [52]. *M. nivale sensu stricto* (that is known to be characterized by high genetic diversity in contrast to *M. majus* [46,51]) was shown to have genetic differences related to different host plants (grasses and cereals) [25] and different turf grass species [53].

Although snow mold disease is of a great economic importance, snow mold-causing pathogens, including *M. nivale*, have been poorly investigated. The snow mold-related pathocomplex and its dynamic during disease progression are poorly characterized in the environment. Although the genetic heterogeneity of *M. nivale* is shown by the example of strains isolated from geographically distant regions and/or from different hosts, the diversity of *M. nivale* within a common agrocenosis and particular host plant has not been analyzed except for turf grass-colonizing varieties [53]. No virulence factors, including major plant cell-wall-degrading enzymes (PCWDEs), of *M. nivale* have been revealed, and the molecular physiological criteria of high or low virulence of these pathogens were not defined. No genome sequences of snow mold-causing fungi are available. In addition, the theoretical bases of plant snow mold resistance are not understood, and only few snow mold-resistant/tolerant cereal cultivars exist [12].

Our study aimed to characterize the community of snow mold-causing *Microdochium* species within a given geographical location and particular crop culture (winter rye). For the first time, fungal and bacterial communities were analyzed by the next-generation sequencing (NGS) technique from snow mold-damaged plants shortly after snowmelt. Twenty-one strains of *M. nivale sensu lato* were isolated from snow mold-damaged rye plants. The strains were classified on the basis of the sequence of the ITS2 region and characterized by morphology, synthesis of extracellular enzymes, and virulence. Our study shows that winter rye plants within a given geographical point are simultaneously colonized by genetically and phenotypically diverse *M. nivale sensu lato* strains. The attribution to a particular genetic group has no correlation with the phenotypes, including the PCWDE activities and virulence. Major PCWDEs were revealed for *Microdochium* species for the first time. Comprehensive characteristics of the isolated *M. nivale sensu lato* strains showed that strains with the highest level of virulence causing similar disease symptoms seem to use their own, unique plant colonization strategies involving the use of “a personal” pattern of extracellular enzymatic activities.

## 2. Materials and Methods

### 2.1. Sample Collection

Samples for isolation and taxonomic comparison of *Microdochium* sp. strains were collected at the field of Federal Research Center “Kazan Scientific Center of Russian Academy of Sciences”, located in the forest–steppe area of the Volga region, Laishev District, Tatarstan Republic, Russia (Universal Transverse Mercator (UTM) north (N) 55.649 east (E) 49.3083). Winter rye (*Secale cereale* L.) cultivar Ogonek having moderate susceptibility to snow mold was used in this study. Plants were grown during the 2018–2019 season under uniform agronomic management (fertilizer, herbicides, etc.) for winter rye production. The samples were collected on 4 May, 10 days after the snow had melted. The disease score was determined according to a 0–4 scale on the basis of visual observation: (0)—no symptoms of the disease; (1)—25%, (2)—50%, (3)—75% of plant parts with disease symptoms; (4)—dead plants [57]. For determination of the taxonomic composition of microbiome using metabarcoding, roots (R-samples), green parts of leaves (GL-samples), and desiccated dead parts of leaves (DL-samples) were collected separately. Each sample type was analyzed in 7–13 biological replicates. The samples were washed several times with distilled water, then held in 70% ethanol for 10 s, and then washed twice in sterile distilled water. Samples for DNA isolation were frozen in liquid nitrogen and held at −83 °C before use. For isolation of *Microdochium* sp. strains, surface-sterilized areas between green parts and desiccated dead parts of leaves, as well as root fragments, were taken and placed on potato sucrose agar (PSA) with 200 µg/mL gentamicin [58].

### 2.2. Isolation of Microdochium sp. Strains, Analysis of Morphology, and Growth Rate

Samples of winter rye (roots and leaves—the boundary zone between green visually healthy and dead desiccated part of leaves) were surface-sterilized and placed on PSA with 200 µg/mL gentamicin. After 10–15 days of incubation at +4 °C, small fragments of mycelium resembling the mycelium of *Microdochium* species [27] were transferred to fresh PSA medium with gentamicin. After 5–10 days of cultivation at 20 °C, actively growing hyphae from the edge of the colony were transferred to fresh PSA medium with gentamicin; this procedure was repeated at least twice. The morphology of the obtained strains preliminary attributed to *Microdochium* sp. was described. Conidia were analyzed using the microscope Biomed-6 (Biomed, Russia).

To assess the growth rates of the isolated strains, 5 mm diameter mycelial plugs cut from the periphery of 10 day old cultures were placed in the center of Petri dishes with PSA medium with 200 µg/mL gentamicin. Fungal cultures were cultivated in darkness at 20 °C for 2 weeks. Every 2 days, two perpendicular radii of a fungal colony were measured. The average of two perpendicular radii was used for the estimation of fungal growth rate. The growth rate of the strains was calculated by determining the slope of the linear regression obtained after plotting the change in the radius of a colony with time. Each strain was analyzed in four biological replicates; the significance of differences in growth rates was performed using the Mann–Whitney *U*-test (*p* < 0.05).

### 2.3. DNA Extraction, DNA Library Preparation, and Sequencing

Total DNA was extracted from the samples using a DNeasy PowerBiofilm Kit (Qiagen, Hilden, Germany) and Fast Prep-24 homogenizer (MP Biomedicals, Solon, OH, USA) according to the protocol provided by the manufacturer. The quality and quantity of extracted DNA were evaluated using NanoDrop 2000 spectrophotometer (Thermo Fisher Scientific, Waltham, MA, USA) and Qubit (Invitrogen, Carlsbad, CA, USA) with high-sensitivity DNA concentration kit, respectively. The ITS2 region of the fungal ribosomal RNA (rRNA) locus was amplified using ITS3_KYO2 (5′–GAT GAA GAA CGY AGY RAA–3′) and ITS4 (5′–TCC TCC GCT TAT TGA TAT GC–3′) primers [59]. Bacterial 16S rDNA libraries were prepared according to the Illumina protocol (Illumina protocol, part no. 15044223, Rev. B). DNA amplification was performed using Bakt_341F (5′–CCT ACG GGN GGC WGC AG–3′) and Bakt_805R (5′–GAC TAC HVG GGT ATC TAA TCC–3′) primers [60] targeting V3 and V4 regions of the 16S rRNA gene. Bacterial 16S rDNA libraries were sequenced on the MiSeq platform using MiSeq Reagent Kit v3 (600-cycles) (Illumina). The libraries containing fungal ITS2 region were sequenced on the MiSeq platform using MiSeq Reagent Kit v2 (500-cycles) (Illumina). All datasets were deposited in the National Center for Biotechnology Information (NCBI) Sequence Reading Archive (SRA) and are available under the PRJNA674969 bioproject.

### 2.4. Bioinformatic Procedures

Raw reads were demultiplexed and quality checked by FastQC v. 0.11.9 (https://www.bioinformatics.babraham.ac.uk/projects/fastqc/). Reads were trimmed against primer sequences using cutadapt v. 2.9 [61]. Then, reads were processed (sequence quality control, including denoising, trimming, and chimera removal) and amplicon sequence variants (ASVs) were generated using DADA2 v.1.14.1 [62]. To assign the taxonomy to ASVs, the naive Bayesian classifier on the Silva v. 132 trainset and UNITE 8.2 database [63] were used for bacterial and fungal datasets, respectively. ASVs assigned to chloroplasts (for bacterial dataset) or unclassified at the kingdom level (for bacterial and fungal datasets) were removed. Bacterial ASVs were analyzed directly. Fungal ASVs were clustered into operational taxonomic units (OTUs) using Swarm v2 [64] with clustering threshold value d = 2. ITSx software [65] was used for the extraction of ITS2 highly variable subregion.

Data were rarefied to the minimum library size and then transformed to centered log-ratio (CLR). Alpha diversity analysis (calculation of richness (Chao 1) and diversity (Simpson) indices) was performed using MicrobiomeAnalyst tool [66]. Significant differences in alpha-diversity indices between sample types were tested with Kruskal–Wallis tests at the OTU/ASV level, *p*-value < 0.01). Minimum count filter (abundance value = 4) was applied before beta-diversity analysis. Nonmetric multidimensional scaling (NMDS) plots were constructed using the Bray–Curtis dissimilarity index with permutational multivariate analysis of variance confirmation. The above-described procedures of OTU generation were applied for the *Microdochium* sp. strains isolated in the present study.

### 2.5. Phylogenetic Classification of Microdochium sp. Isolates

Nucleotide sequences of ITS2 of *Microdochium* species (*M. nivale*, *M. majus*, *M. bolleyi*, *M. poae*, *M. colombiense*) were collected from the NCBI nucleotide databank. The sequences that displayed >90% identity and coverage with the sequences of the isolated strains were considered. Then, 164 bp fragments of ITS2 of *Microdochium* species were used for multiple sequence alignment (ClustalW 1.6 algorithm for non-coding sequences); the alignment was visualized by WebLogo3 [67]. The phylogenetic tree was built using the Bayesian inference method implemented in the MrBayes program (v3.2.6) [68]. The alignment and phylogenetic tree visualization were performed using the MegaX [69].

### 2.6. Enzymatic Activity Assays

The extracellular activities of eight enzymes (cellulase (endoglucanase), xylanase, arabinofuranosidase, pectate lyase, lignin peroxidase, protease, amylase, and invertase) were measured in the culture supernatants of the isolated *Microdochium nivale* strains. Fifty milliliters of liquid potato sucrose medium was inoculated with three 5 mm diameter mycelial plugs cut from the periphery of 10 day old cultures grown on potato sucrose agar. After 20 and 30 days of cultivation, 10 mL aliquots of the cultures were collected. Traces of fungal mycelium were removed by centrifugation at 13,000× *g*, 10 min, 4 °C. The supernatants were stored frozen at −20 °C until further use. The enzymatic activities were measured in three biological and three technical replicates using a PB2201B spectrophotometer (SOLAR, Belarus); the sterile growth medium was used as a blank.

The amylase, cellulase (endoglucanase), xylanase, and invertase activities were determined by measuring the reducing sugars released after enzymatic hydrolysis of the corresponding substrates. The reducing sugars were measured using 3,5-dinitrosalicylic acid (DNS reagent) (Sigma, Saint Louis, MO, USA) at 540 nm [70]. Amylase activity was determined by measuring the decomposition of soluble starch (Sigma, Saint Louis, MO, USA) [71]. The starch was dissolved by heating (45 °C) in 50 mM in a citrate buffer (pH 5.5). Then, 125 μL of the cultural supernatant were mixed with 125 μL of 1% starch solution and incubated 30 min at 37 °C. Cellulase (endoglucanase) activity was determined using carboxymethyl cellulose as a substrate (Sigma, Saint Louis, MO, USA) [72]. First, 250 μL of the cultural supernatant were mixed with 250 μL of 2% carboxymethyl cellulose in 100 mM citrate buffer (pH 5.5) and incubated 30 min at 50 °C. For the determination of xylanase activity, the beechwood xylan (Sigma, Saint Louis, MO, USA) was used [73]. Then, 10 μL of the cultural supernatant was mixed with 490 μL of 1% xylan in 50 mM phosphate buffer (pH 7.0) and incubated 30 min at 55 °C. Invertase activity was determined by incubating 600 μL of culture supernatant with 150 μL of 150 mM sucrose in 20 mM phosphate buffer (pH 6.2) at 37 °C for 3 h [74]. The above-described reactions were stopped by heating at 100 °C, 5 min before the analysis of products by DNS reagent. One unit (U) of activities was defined as the amount of enzyme releasing 1 µmol of reducing sugars (glucose or xylose)/min per mg of protein.

For the determination of protease activity, 200 μL of 2% casein sodium sault (Sigma, Saint Louis, MO, USA) in deionized water was mixed with 100 μL of the cultural supernatant and incubated 30 min at 37 °C. Then, the reactions were stopped by the addition of 500 μL of 5% trichloroacetic acid (Sigma, Saint Louis, MO, USA). The sediment was removed by centrifugation at 5000× *g*, 10 min, 25 °C. Then, 100 μL of the supernatant, 400 μL of 6% Na_2_CO_3_, and 100 μL of Folin–Ciocalteu’s phenol reagent (Supelco, Darmstadt, Germany) were mixed and incubated 30 min at 25 °C. Tryptophan released from the casein was measured at 600 nm [75]. One unit of protease activity was defined as the amount of enzyme releasing 1 µmol of tryptophan/min per mg of protein.

Arabinofuranosidase activity was determined by the rate of conversion of 4-nitrophenyl α-l-arabinofuranoside (4NPA) (Megazyme, Bray, Ireland) to 4-nitrophenol [76]. First, 50 μL of 10 mM 4NPA in 50 mM citrate buffer (pH 5.0) was mixed with 450 μL of the cultural supernatant and incubated with shaking (600× *g*) at 37 °C for 2 h. Then, the reactions were stopped by the addition of 500 μL of 1 M Na_2_CO_3_. The samples were centrifuged for 10 min at 10,000× *g* and the optical density (OD) was measured at 405 nm. One unit of arabinofuranosidase activity was defined as the amount of enzyme releasing 1 µmol of 4-nitrophenol/min per mg protein.

Pectate lyase activity was determined by measuring the degradation of polygalacturonic acid (PGA) into unsaturated products [77]. First, 435 μL of 0.25% PGA (Sigma, Saint Louis, MO, USA) in 50 mM Tris-HCl buffer (pH 8,5) was mixed with 50 μL of 10 mM CaCl_2_ and 50 μL of the cultural supernatant at 37 °C. The accumulation of the unsaturated products was measured at 234 nm. One unit of pectate lyase activity was defined as the amount of enzyme releasing 1 µmol unsaturated products/min per mg of protein.

The activity of lignin peroxidase was determined by the rate of oxidation of veratryl alcohol to veratraldehyde [78]. First, 140 μL of 2 mM veratryl alcohol (Acros Organics, Fair Lawn, NJ, USA) in sodium tartrate buffer (100 mM, pH 3.0) was mixed with 10 μL of 0.4 mM H_2_O_2_ and 50 μL of the cultural supernatant at 25 °C. The accumulation of the veratraldehyde (ε = 9.3 mM^−1^·cm^−1^) was measured at 310 nm. One unit of lignin peroxidase activity was defined as the amount of enzyme releasing 1 µmol veratraldehyde/min per mg protein. Protein concentration was assayed by the Bradford method [79]. The calculation of Pearson’s correlation coefficient for different enzymatic activities and growth rate of the strains, as well as the generation of a heat map showing the pattern of enzymatic activities for each strain, was performed using R (version 3.6).

### 2.7. Virulence Assay

The virulence of the isolated *Microdochium nivale sensu lato* strains was assessed toward winter rye (*Secale cereale* L.) cultivar Ogonek obtained from Tatar Scientific Research Institute of Agriculture (Kazan, Russia). Seeds were washed and sterilized using 1% SDS (2 times for 10 min each), 0.01% potassium permanganate (for 10 min), and sodium hypochlorite (1% and 5% for 5 min each), then washed five times with sterile distilled water, and transferred to water agar (pH 5.8). Seeds were germinated for 2 days at 28 °C in darkness. Seedlings were transferred to individual sterile 50 mL glass tubes with 7 mL of ¼ diluted Murashige and Skoog medium without organic carbon. Simultaneously, the infection with the isolated strains was performed by placing an 8 mm mycelial plug (cut from the periphery of 10–14 day old cultures grown on PSA) into the tube in contact with the seedling. For the control plants, 8 mm plugs of sterile PSA were used instead of the mycelial plugs. Control and infected plants were grown at 20 °C with a 16 h light/8 h dark cycle photoperiod for 20 days. A total of 12–16 biological replicates were analyzed for each of the experimental variants. Twenty days after infection, the number of plants displaying necrosis on leaves and stems, as well as plants showing brownish root pigmentation, were counted and the length of roots and shoots, the quantity of roots and leaves, and the fresh weight of roots and shoots were measured.

The results were analyzed using XLSTAT Statistical Analysis Software (2020.4.1.1018). Pearson’s correlation analysis was performed to assess the correlation between the development of different symptoms or between the degree of virulence and enzymatic activities, growth rate, and attribution to the particular OTU. Cluster analysis of the isolated strains according to the degree of virulence (manifestation of different symptoms on the host plant) was performed using agglomerative hierarchical clustering with unweighted and pair group average method according to the similarity Pearson correlation coefficient. To verify the assembled clusters of the isolated strains, one-way analysis of variance (ANOVA) to determine a significant *F*-test (*p* < 0.05) and a multiple comparison using Duncan’s multiple range test (*p* < 0.05) were performed.

## 3. Results

### 3.1. Analysis of the Taxonomic Composition of the Microbiome of Snow Mold-Affected Rye Plants

**Figure 1:**
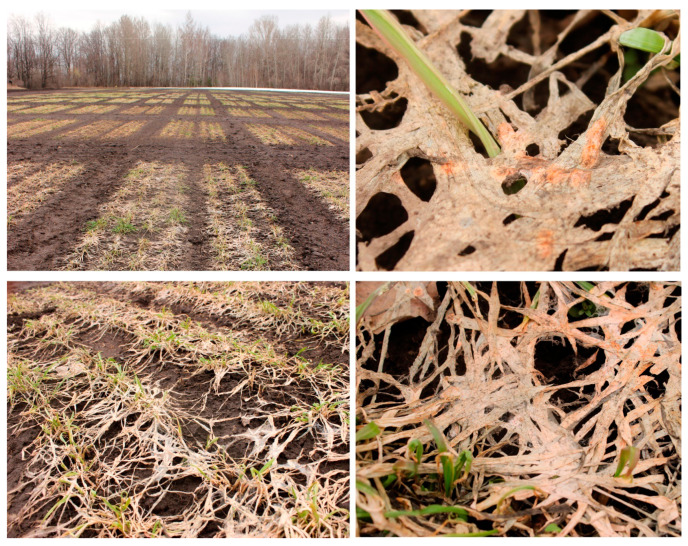
Snow mold-damaged winter rye plants (Tatarstan, Russia, 2019).

The samples of the snow mold-affected winter rye plants (roots (R-samples), green parts of leaves (GL-samples), and desiccated dead parts of leaves (DL-samples) were collected separately in the field of the Federal Research Center “Kazan Scientific Center of Russian Academy of Sciences”. Due to the snow mold epiphytotia in the Volga region in 2018–2019 [11], the plants were severely damaged (disease score 3) (Figure 1). Snow mold progression was expressed in the visible symptoms of tissue damage and necrosis, as well as in a significant growth of the mycelium on the leaves, tillers, and soil surface. The fungal and bacterial communities of the snow mold-affected winter rye plants were characterized by analyzing the fungal ITS2 region and bacterial V4 region of the 16S rRNA gene using NGS techniques.

In total, 3,945,809 (Table S1, Supplementary Materials) and 2,839,056 (Table S2, Supplementary Materials) reads were obtained for the amplicons synthesized using ITS2- and 16S rRNA-specific primers, respectively. From the pool of reads obtained for amplicons synthesized using ITS2-specific primers, after the read filtration, dereplication, and chimera removal, 1296 amplicon sequence variants (ASVs) were generated. Then, 366 ASVs were excluded from the subsequent analysis because their sequences did not correspond to fungi. A total of 930 ASVs assigned to fungi were clustered into 678 operational taxonomic units (OTUs). The clustered 678 OTUs (Tables S3 and S4, Supplementary Materials) comprising the “fungal dataset” belonged to 250 fungal genera.

A total of 2448 ASVs were generated from the pool of the processed reads obtained for amplicons synthesized using 16S rRNA-specific primers. Then, 609 ASVs assigned to fungi and plants were excluded from the subsequent analysis. The 1839 ASVs assigned to bacteria (Tables S5 and S6, Supplementary Materials) were analyzed directly without clustering. The revealed 1839 ASVs comprising the “bacterial dataset” belonged to 223 bacterial genera.

**Figure 2:**
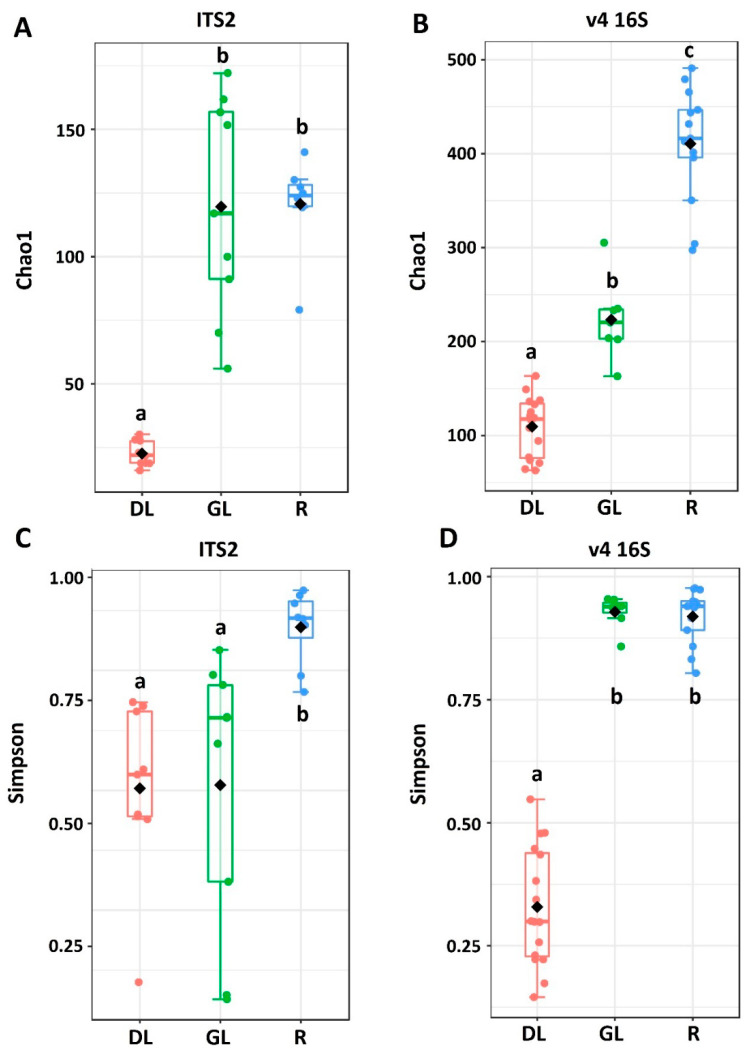
Richness (Chao1 index) (**A**,**B**) and diversity (Simpson index) (**C**,**D**) of the fungal (**A**,**C**) and bacterial (**B**,**D**) communities of winter rye plants affected by the snow mold disease. Desiccated dead parts of leaves (DL), green parts of leaves (GL), and roots (R) were analyzed separately. Different letters on the bars indicate significant differences between different sample types (Kruskal–Wallis tests at the operational taxonomic unit (OTU) level, *p*-value < 0.01).

Chao1 and Simpson indices were calculated to estimate the richness and diversity, respectively, of the fungal and bacterial communities in GL-, DL-, and R-samples of snow mold-affected rye (Figure 2). The Chao1 estimator [80,81] calculates the estimated true species diversity of a sample. Simpson’s diversity index is used to calculate a measure of diversity, considering the number of species, as well as its relative abundance. An increase in the Simpson index in the range from 0 to 1 indicates an increase in the contribution of dominant species to the population of the community. The richness of fungal community (Chao1 index) did not differ in R- and GL-samples (101 and 96 OTUs, respectively), whereas it was lower in DL-samples (20 OTUs) (Figure 2). In turn, the richness of the bacterial community differed in all three sample types: R-samples—397 ASVs, GL-samples—207 ASVs, and DL-samples—83 ASVs. The diversity (Simpson index) of fungal community was lower in GL- and DL-samples compared to R-samples. Furthermore, the intragroup variability of the Simpson index was large for GL-samples pointing to a greater variability of the fungal community in green leaves compared to roots or desiccated leaves. For the bacterial community, the diversity in GL-samples was as high as in R-samples, while it was lower in DL-samples (Figure 2).

**Figure 3:**
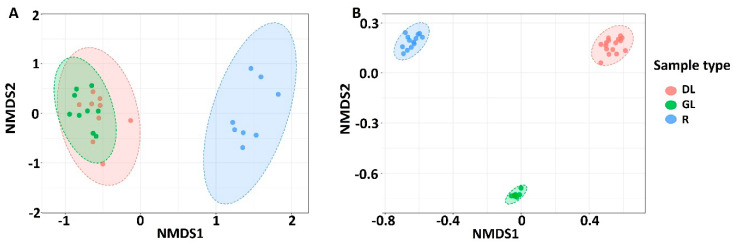
Nonmetric multidimensional scaling (NMDS) analysis of the fungal (**A**) and bacterial (**B**) communities of winter rye plants affected by the snow mold disease. Desiccated dead parts of leaves (DL, red), green parts of leaves (GL, green), and roots (R, blue) were analyzed separately on the basis of Bray–Curtis distance. Statistical analysis of group similarities showed a significant difference between clusters DL/GL and R (PERMANOVA, *p*-value < 0.001) for the fungal dataset and among all clusters for the bacterial dataset (PERMANOVA, *p*-value < 0.001).

To examine if the composition of the fungal and bacterial communities differed depending on the sample type (R-, GL-, DL-samples), nonmetric multidimensional scaling (NMDS) plots were constructed on the basis of Bray–Curtis dissimilarity (Figure 3). NMDS analysis of the fungal dataset showed that the samples of aboveground plant parts formed a mutual cluster distinct from the R-sample cluster (PERMANOVA, *p*-value < 0.001). In turn, for the bacterial dataset, each sample type formed a distinct cluster (Figure 3). This means that the fungal communities were more or less similar in green leaves and desiccated leaves, while the bacterial communities were significantly different.

**Figure 4:**
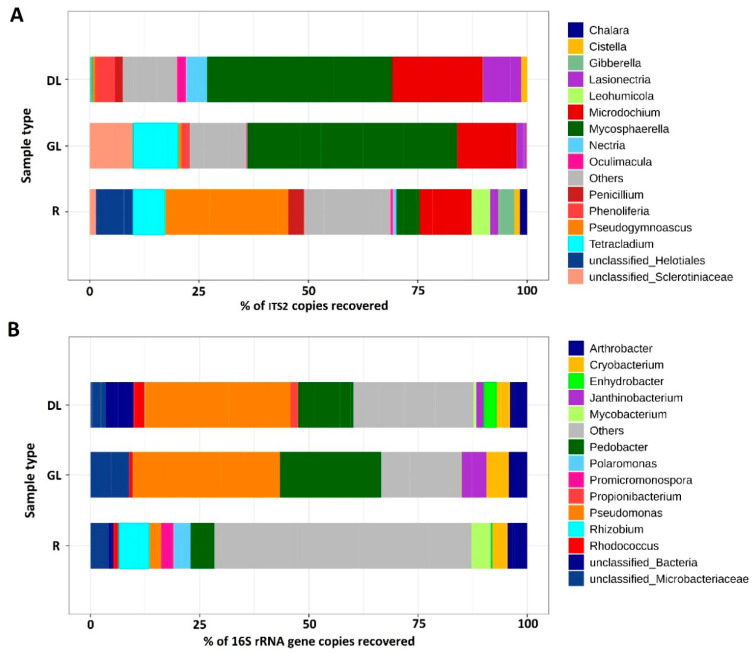
The taxonomy composition of the fungal (**A**) and bacterial (**B**) communities of winter rye plants affected by the snow mold disease. Desiccated dead parts of leaves (DL), green parts of leaves (GL), and roots (R) were analyzed separately. Each color represents a relative abundance of the particular fungal or bacterial genus in the samples. The genera with the abundance of ≥10% in at least one of the analyzed samples are presented; the other genera are merged into the category “others”. The relative abundance of the particular genus was determined by calculating the percentage of genus-corresponding OTUs in fungal/bacteria datasets.

The most abundant phylum within the fungal dataset was *Ascomycota*: from 88.1% in GL- samples to 96.8% in R-samples. The *Basidiomycota* represented 1.5% (R-samples) to 11.4% (GL-samples). *Mortierellomycota*, *Mucoromycota*, and *Olpidiomycota* constituted less than 1% of the fungal dataset (Table S4, Supplementary Materials). At a genus level, two genera dominated in the DL- and GL-samples: *Microdochium*—some species of which cause the snow mold disease (36.7% and 16.5% of the fungal dataset in DL- and GL-samples, respectively) and *Mycosphaerella*—which includes well-known phytopathogenic species (33.5% and 42.1% of the fungal dataset in DL- and GL-samples, respectively) (Figure 4). In addition, the members of fungal genera *Tetracladium, Lasionectria, Cistella, Phenoliferia, Nectria, Oculimacula,* and *Penicillum* were represented in the DL- and/or GL-samples (Table S7, Supplementary Materials). In the R-samples, the members of *Microdochium* and *Mycosphaerella* were also present (9.8% and 7.2%, respectively); however, the dominant genus was *Pseudogymnoascus* (27.6%). In addition, the members of fungal genera *Cistella*, *Lasionectria*, *Nectria*, *Oculimacula*, *Penicillum*, and *Tetracladium* (that were also revealed in DL- and/or GL-samples), as well as *Chalara*, *Gibberella*, and *Leochumicola* (that were not revealed in DL- and/or GL-samples), were represented in the R-samples (Figure 4, Table S4, Supplementary Materials).

Within a bacterial community of snow mold-affected rye plants, the representatives of phyla Proteobacteria, Actinobacteria, Bacteroidetes, and Firmicutes were revealed in GL- (47.9%, 22.4%, 26.0%, and 3.6%, respectively), DL- (53.0%, 21.4%, 18.0%, and 3.7%, respectively), and R- (43.8%, 43.9%, 8.0%, and 2.4%, respectively) samples (Table S6, Supplementary Materials). The major classes of bacteria were, in general, similarly represented in the GL- and DL-samples: Gammaproteobacteria (34.0% and 38.0%, respectively), Actinobacteria (22.0% and 26.0%, respectively), and Sphingobacteria (22.9% and 17.1%, respectively). In the R-samples, Actinobacteria (42.8%), Alphaproteobacteria (27.5%), and Betaproteobacteria (10.2%) were the most represented classes (Table S8, Supplementary Materials). At the genus level, GL- and DL-samples were also similar, in general, with *Pseudomonas* (33.8% and 34.5%, respectively) and *Pedobacter* (22.8% and 17.0%, respectively) as the dominant genera (Figure 4). NMDS analysis showed that the bacterial communities in GL- and DL-samples were significantly different (see above, Figure 3). This likely means that visually healthy leaves and dead leaves are colonized by different varieties (species/strains having different ASV variants) of the similar genera. In the R-samples, no clearly dominant genera of bacteria were revealed (Figure 4); the bulk of the root colonizing bacterial community (63.3%) was not identified at the genus level (Table S9, Supplementary Materials). The presence of a large amount of nonidentified bacteria is typical of rhizosphere communities [82,83].

**Figure 5:**
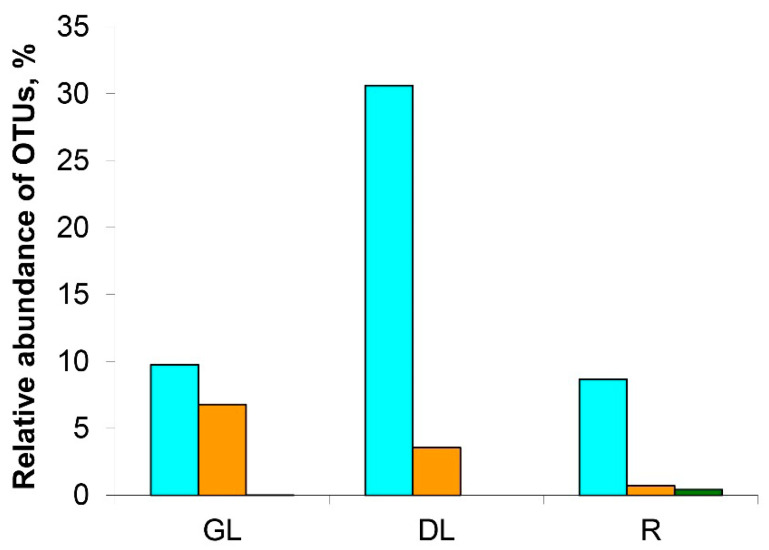
Relative abundance of *Microdochium*-related OTUs (M.OTU1—blue; M.OTU2—orange; M.OTU3—green) in the pool of fungal OTUs of snow mold-damaged winter rye. The mycobiomes of green leaves (GL), desiccated dead leaves (DL), and roots (R) were analyzed separately.

Since our study emphasized snow mold disease, we gave special attention to the composition of representatives of the *Microdochium* genus in the diseased plants. Three different OTU variants related to *Microdochium* genus were found (M.OTU1, M.OTU2, and M.OTU3, referred to as OTU4, OTU8, and OTU57 in the raw dataset; Tables S1 and S10, Supplementary Materials) within the fungal dataset (Figure 5).

All three variants together were revealed only in R-samples (8.6%, 0.7%, and 0.4% of fungal dataset for M.OTU1, M.OTU2, and M.OTU3, respectively). In the GL- and DL-samples, M.OTU3 was absent. M.OTU1 and M.OTU2 were represented rather equally in GL-samples (9.7% and 6.8%, respectively); on the other hand, in the DL-samples, M.OTU1 was strongly dominant compared to M.OTU2 (30.6% and 3.6%, respectively) (Figure 5). This means that *Microdochium* species/strains belonging to M.OTU1 have an advantage over the representatives of M.OTU2 as the host plant tissues collapse.

### 3.2. Isolation of Microdochium Strains and Their Primary Characteristics

Twenty-one strains that were preliminary attributed to *Microdochium* genus according to the mycelium morphology were isolated from winter rye. All strains are maintained in the collection of the Laboratory of Plant Infectious Diseases in the Federal Research Center “Kazan Scientific Center of Russian Academy of Sciences”. The strains are referred to as 1–21 throughout the paper; the corresponding accession numbers in the collection are given in Table S11 (Supplementary Materials). Nineteen strains were isolated from leaves and two (No. 7 and 17) were isolated from roots.

**Figure 6:**
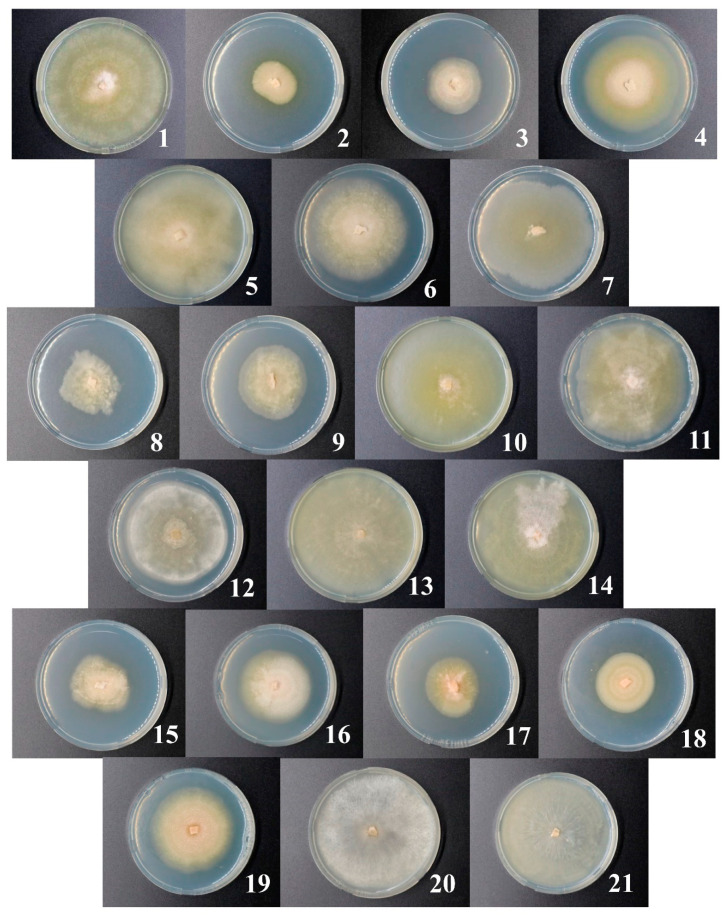
Morphology of the isolated candidate *Microdochium* sp. strains. The number on a photo corresponds to the number of a strain. The photos were made 14 days after inoculation.

Strains differed in their morphology. Mycelium color was whitish, creamy, or light pink depending on the strain (Figure 6). Most (but not all) strains produced a (pale) yellow–ochraceous exudate. Aerial mycelium characteristics of different strains varied: pellicular, farinaceous, floccose, felty, cottony, and silky; in two of the strains, the aerial mycelium was absent. The marginal zones of the fungal colonies were even or uneven. The zonation of the colonies of different strains was radial or concentric, sometimes with solid segments; for some of the strains, the zonation was not pronounced. Details about the morphology of the strains are given in the Table S12 (Supplementary Materials).

**Figure 7:**
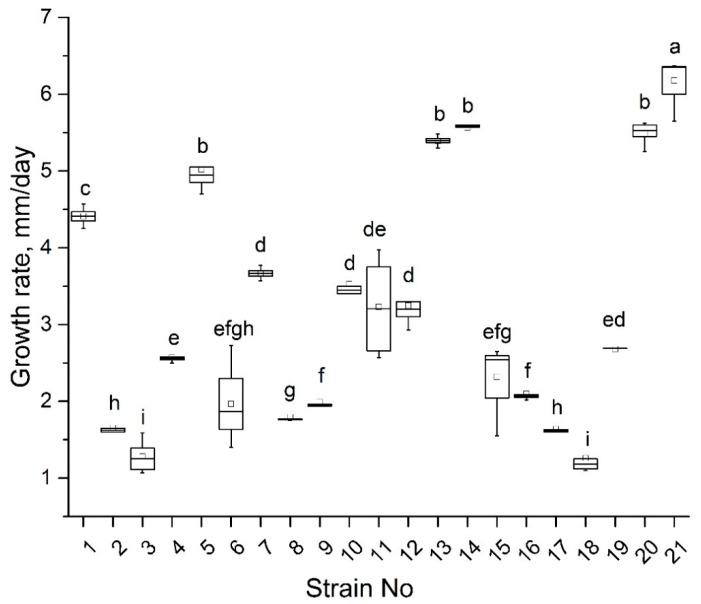
Growth rate of the isolated *Microdochium* sp. strains. Boxes that do not share the same letter have significantly different medians in pairwise comparison (Mann–Whitney test, *p* < 0.05). The experiment was performed in four biological replicates.

The isolated strains had different growth rates (Figure 7) ranging from 1 to 6 mm per day. The strains 1, 5, 13, 14, 20, and 21 were characterized by the fastest growth, while the growth rate of strains 2, 3, 8, 17, and 18 was the lowest. Seven strains (1, 3, 5, 10, 14, 17, and 19) formed conidia typical of *M. nivale sensu stricto* [17] with 0–3 septa and sizes of 10.1–20.3 µm length and 1.8–4.1 µm width depending on the strain (Table S13, Supplementary Materials).

### 3.3. ITS Sequencing and Phylogenetic Classification of Microdochium sp. Strains

To check if the isolated strains belonged to *Microdochium* sp. and to attribute the strains to the particular species, the 164 bp fragments of ITS2 regions were sequenced for all of the 21 strains. ITS2 sequence analysis proved that all the strains belonged to *Microdochium* sp. Four different OTUs were revealed: M.OTU1, M.OTU2 (that were also revealed among the most represented OTUs in the microbiome of rye, see above), M.OTU4, and M.OTU5 (that were not revealed among the most represented in the microbiome of rye) (Table S14, Supplementary Materials). The strains 1, 2, 4, 5, 6, 7, 13, 14, 15, 18, and 19 belonged to M.OTU1; the strains 3, 9, 10, 12, 16, 17, 20, and 21 belonged to M.OTU2; the strains 11 and 8 belonged to M.OTU4 and M.OTU5, respectively (Table S14, Supplementary Materials). M.OTU3 (that was revealed as a minor OTU among the OTUs that belonged to *Microdochium* sp. in the microbiome of rye) was not revealed among the isolated strains.

**Figure 8:**
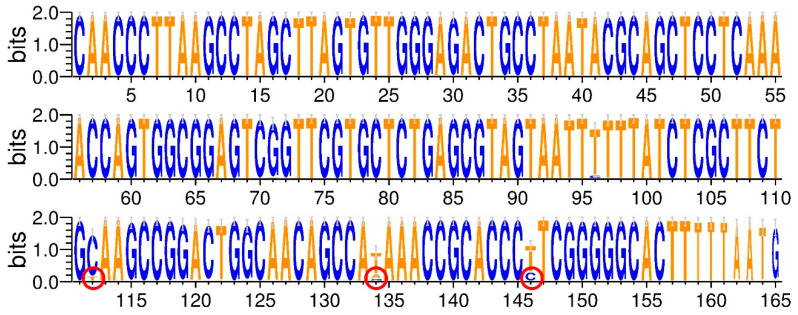
WebLogo3 visualization of ClustralW multiple alignment of 164 bp fragments of ITS2 of 24 *Microdochium majus* and 85 *M. nivale sensu stricto* strains. Some (32 of 85) of the *M. nivale sensu stricto* strains have species-specific positions (T112, A/G134, and C146; marked by red circle) that distinguish them from all known *M. majus* strains. Other *M. nivale sensu stricto* strains (53 of 85) have the same nucleotide positions as all *M. majus* strains (C112, T134, and T146).

To rank the isolated strains to the particular species of *Microdochium* genus, the ITS2 sequences corresponding to M.OTU1–M.OTU5 were compared with the most similar sequences in NCBI nucleotide database (Tables S15–S19, Supplementary Materials). Although ITS2 is extensively used for phylogenetic classification of fungi, some authors doubt that this DNA region is suitable for the distinguishing of closely related *M. majus* and *M. nivale sensu stricto* species [84]. To check if the analyzed 164 bp region contains unique, *M. nivale sensu stricto* or *M. majus* species-specific positions, corresponding DNA regions of 24 *M. majus* and 85 *M. nivale sensu stricto* strains were compared. We found that, within the analyzed 164 bp region, many (32 of 85) strains of *M. nivale sensu stricto* have at least one species-specific nucleotide position (T112 (11 of 85 strains), A/G134 (28 of 85 strains), and C146 (24 of 85 strains)) that distinguishes these *M. nivale sensu stricto* strains from all the *M. majus* strains (Figure 8). However, some of *M. nivale sensu stricto* strains do not have specific nucleotide positions within the analyzed 164 bp region that distinguish them from *M. majus*. Thus, some of *M. nivale sensu stricto* genotypes can be identified at the species level on the basis of the analyzed DNA region, but some of *M. nivale sensu stricto* genotypes cannot be distinguished from the closely related *M. majus* species.

According to species-specific nucleotide positions, M.OTU2 and M.OTU5 (identified among the strains isolated in our study) were characteristic of *M. nivale sensu stricto* but not of *M. majus*. M.OTU2 and M.OTU5 displayed 98.17% similarity (E-value = 4 × 10^−82^) with each other at full coverage. Therefore, the strains 3, 9, 10, 12, 16, 17, 20, 21 (M.OTU2), and 8 (M.OTU5) were attributed to *M. nivale sensu stricto*. M.OTU1 and M.OTU4 (coverage = 99%, percentage identity = 100.00%, E-value = 1 × 10^−86^) were characteristic of both *M. nivale sensu stricto* and *M. majus*, since species-specific nucleotide positions (T112, A/G134, and C146) were not present within the corresponding ITS2 regions. Therefore, we could not attribute the strains 1, 2, 4, 5, 6, 7, 13, 14, 15, 18, 19 (M.OTU1), and 9 (M.OTU4) to the particular *Microdochium* species (*M. nivale sensu lato*). However, given that the strains 1, 5, 14, and 19 formed the conidia typical of *M. nivale sensu stricto*, it is reasonable to speculate that M.OTU1- and M.OTU4-corresponding strains isolated in our study also belonged to *M. nivale sensu stricto*. The M.OTU3 revealed in the rye microbiome but not within the isolated strains corresponded to *M. bolleyi*.

**Figure 9:**
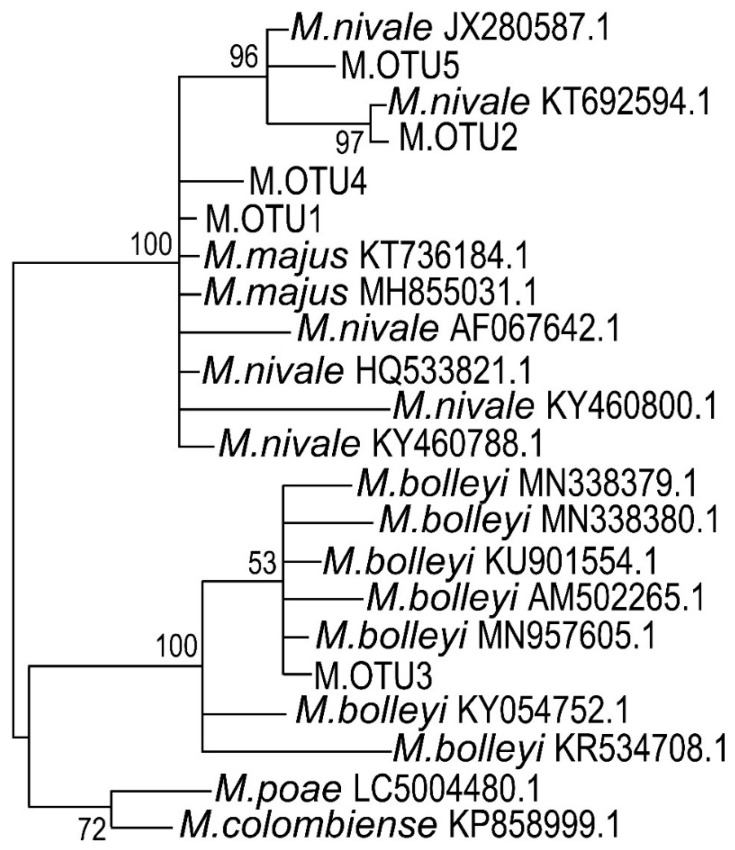
The phylogram of some of the *Microdochium* species built using the Bayesian inference method (MrBayes v3.2.6) on the basis of the 164 bp fragment of ITS2. The phylogram was rooted on midpoint and visualized by MEGAX. Since the sequences of strains of a particular species are usually 100% identical within the analyzed DNA region, the NCBI accession numbers are given only for one of these strains.

To illustrate the phylogenetic positions of M.OTU1–M.OTU5, the phylogram of *Microdochium* species was constructed on the basis of a 164 bp fragment of ITS2 (Figure 9). ITS2 regions of *M. nivale*, *M. majus*, *M. bolleyi*, *M. poae*, and *M. colombiense* that have high level of identity (90–100%) with ITS2 regions of M.OTU1–M.OTU5 were included in the alignment; the corresponding phylogram is presented in Figure 9. M.OTU1, 2, 4, and 5 together with *M. majus* and *M. nivale sensu stricto* formed a common clade due to high sequence similarity (98–100%). Another clade was formed by *M. poae* and *M. colombiense* (first subclade), as well as by OTU3 and *M. bolleyi* (second subclade). The obtained topology of the phylogram perfectly matched the phylogenetic trees constructed previously for *Microdochium* species on the basis of more prolonged sequences of large subunit ribosomal ribonucleic acid (LSU rRNA), small subunit ribosomal ribonucleic acid (SSU rRNA), beta-tubulin, and other genes [85,86,87].

### 3.4. Extracellular Enzyme Activities of the Isolated M. nivale Strains

Most if not all phytopathogenic microorganisms use extracellular enzymes, predominantly plant cell-wall-degrading enzymes (PCWDEs), to colonize host plant tissues. For *Microdochium* species, the extracellular enzymes have not been characterized except for glucosidase, galactosidase [44], and invertase [74]. To check, if *Microdochium* species produce host metabolite-directed extracellular enzymes potentially involved in virulence and if the isolated *Microdochium* strains differ in the level of production of these enzymes, the activities of extracellular cellulase, xylanase, arabinofuranosidase, pectate lyase, lignin peroxidase, protease, amylase, and invertase were measured in the culture supernatants.

**Figure 10:**
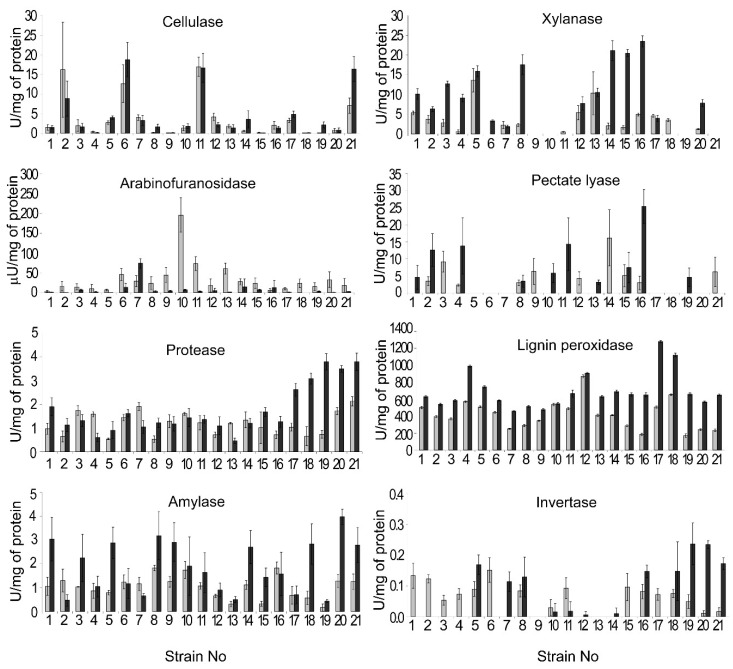
Extracellular enzymatic activities of the isolated *Microdochium nivale* strains. Light-gray column—20 days of cultivation; dark-gray column—30 days of cultivation. The presented values are averages ± SD of three biological replicates.

All of the isolated strains had a pronounced level of extracellular cellulase (endoglucanase), protease, lignin peroxidase, and amylase activities on both the 20th and the 30th days of cultivation (Figure 10). However, the levels of these activities varied between the strains: from 0.05 U/mg and 0.05 U/mg (20th day and 30th day, respectively) to 16.9 U/mg and 18.8 U/mg (20th day and 30th day, respectively) for cellulase (endoglucanase), from 0.5 U/mg and 0.5 U/mg (20th day and 30th day, respectively) to 2.1 U/mg and 3.8 U/mg (20th day and 30th day, respectively) for protease, from 170.8 U/mg and 460.1 U/mg (20th day and 30th day, respectively) to 867.9 U/mg and 1278.5 U/mg (20th day and 30th day, respectively) for lignin peroxidase, and from 0.2 U/mg and 0.4 U/mg (20th day and 30th day, respectively) to 1.8 U/mg and 4.0 U/mg (20th day and 30th day, respectively) for amylase (Figure 10).

Extracellular xylanase activity was below the detectable level in the cultural supernatants of the strains 9, 10, 19, and 21 on both the 20th and the 30th days of cultivation. For the other strains, extracellular xylanase activity ranged from 0.5 U/mg and 1.9 U/mg (20th day and 30th day, respectively) to 13.6 U/mg and 23.5 U/mg (20th day and 30th day, respectively) (Figure 10). Only trace amounts of extracellular arabinofuranosidase activity were detected for most of the strains. Strain 10 displayed the highest level of arabinofuranosidase activity (196 µU/mg) on the 20th day of cultivation that was decreased by the 30th day of cultivation (Figure 10). Extracellular pectate lyase activity was below the detectable level for the strains 5–7, 17, 18, and 20 on both the 20th and the 30th days of cultivation. For the other strains, the pectate lyase activity level ranged from 2.3 U/mg and 3.2 U/mg (20th day and 30th day, respectively) to 16.2 U/mg and 25.4 U/mg (20th day and 30th day, respectively) (Figure 10). Invertase activity was below the detectable level for the strains 9 and 13 on both the 20th and the 30th days of cultivation. For the other strains, the invertase activity level ranged from 0.011 U/mg and 0.006 U/mg (20th day and 30th day, respectively) to 0.15 U/mg and 0.23 U/mg (20th day and 30th day, respectively) (Figure 10).

**Figure 11:**
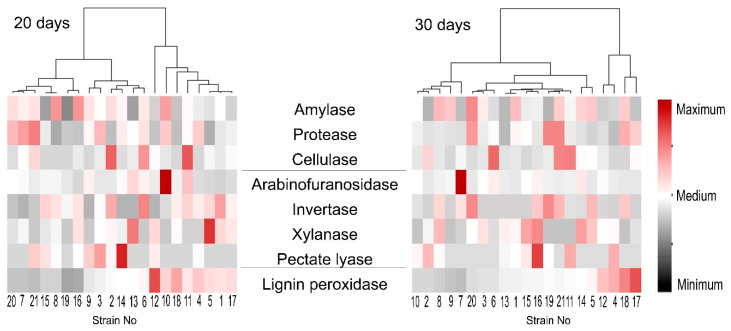
Heat map showing the relative patterns of extracellular enzymatic activities of different *Microdochium nivale* strains. The colors correspond to the level of activity (red (maximum), white (medium), and gray (minimum)) relative to the activity levels of all of the analyzed strains. The values (colors) are normalized in relation to a given activity of different strains but not in relation to different enzymatic activities. The heat map was made using R (version 3.6).

Then, we assessed if the levels of one of the revealed enzymatic activities correlated (positively or negatively) with that of any other one or with the growth rates of the strains. No correlations between the levels of different activities or between the activities and growth rate were revealed (Table S20, Supplementary Materials). To compare the patterns of extracellular enzymatic activities of the strains and to reveal the most “cognate” strains in terms of extracellular activities, a heat map was generated. The heat map showed that each strain had a unique pattern of extracellular enzymatic activities (Figure 11). Moreover, strains that clustered together as more related in terms of the pattern of enzymatic activities measured on the 20th day of cultivation appeared in distant clusters by the 30th day of cultivation (Figure 11).

### 3.5. Virulence of the Isolated M. nivale Strains

**Figure 12:**
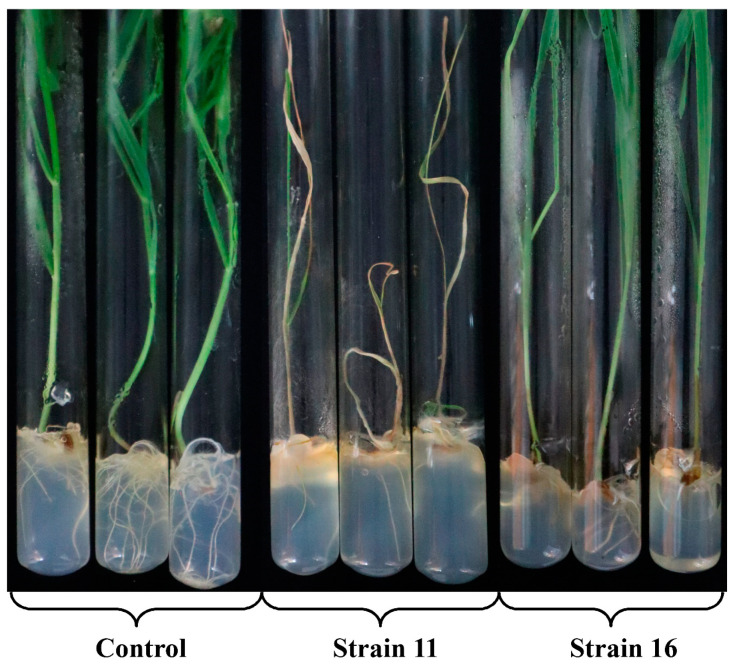
Rye plants (cultivar Ogonek) infected with the isolated *Microdochium nivale* strains (11 and 16). Control—noninfected plants. The presented photos demonstrate the infection (20th day) caused by one of the highly virulent strains (11), which was associated with a severe stem and leaf necrosis and reduction of root biomass, and by one of the moderately virulent strains (16), which did not cause severe damage to the aboveground plant parts but strongly reduced root biomass. Photos of the plants infected by all of the isolated strains are given in Figure S1 (Supplementary Materials).

To compare the virulence of the isolated *M. nivale* strains, rye plants grown under sterile conditions from surface sterilized seeds were infected. Twenty days after infection, the percentage of plants displaying necrosis on leaves and stems, as well as plants showing brownish root pigmentation were scored (Figure 12 and Table 1; Figure S1 and Table S21, Supplementary Materials). The length of roots and shoots, the quantity of roots and leaves, and the fresh weight of roots and shoots were measured (Table 1; Table S21, Supplementary Materials). More than 50% of plants displayed necrosis on leaves and stems after infection by the strains 1, 5, 6, 8, 9, 11–14, 21 (Table 1; Table S21, Supplementary Materials). In addition, infection with strains 7 and 15 led to stem necrosis in 56% and 100% of plants, respectively, but the necrosis on leaves was manifested much less (19% and 47%, respectively). The manifestation of necrosis was coupled with root pigmentation. Furthermore, infection by some of the strains (10, 16) led to extensive root pigmentation without manifestation of necrosis.

The infection by some of the strains caused a reduction in root length (from 12.3 cm in the control plants to 2.6 cm in the plants infected by the strain 15), shoot length (from 37.8 cm in the control plants to 16.1 cm in the plants infected by the strain 21), leaf quantity (from 4.2 in the control plants to 2.4 in the plants infected by the strain 21), fresh weight of roots of 10 plants (from 2.3 g in the control plants to 0.3 g in the plants infected by the strains 11 and 21), and fresh weight of shoots of 10 plants (from 5.8 g in the control plants to 0.8 g in the infected by the strains 11 and 21) (Table S21, Supplementary Materials). Interestingly, although the weight and the length of roots were decreased in the plants infected by most of the strains, the quantity of roots was increased in the plants infected by some of the strains (from 6.8 in control plants to 8.8 in the infected by strain 7) (Table S21, Supplementary Materials). In total, the reduction in the length and weight of roots after infection was more pronounced than the reduction in the length and weight of shoots.

**Figure 13:**
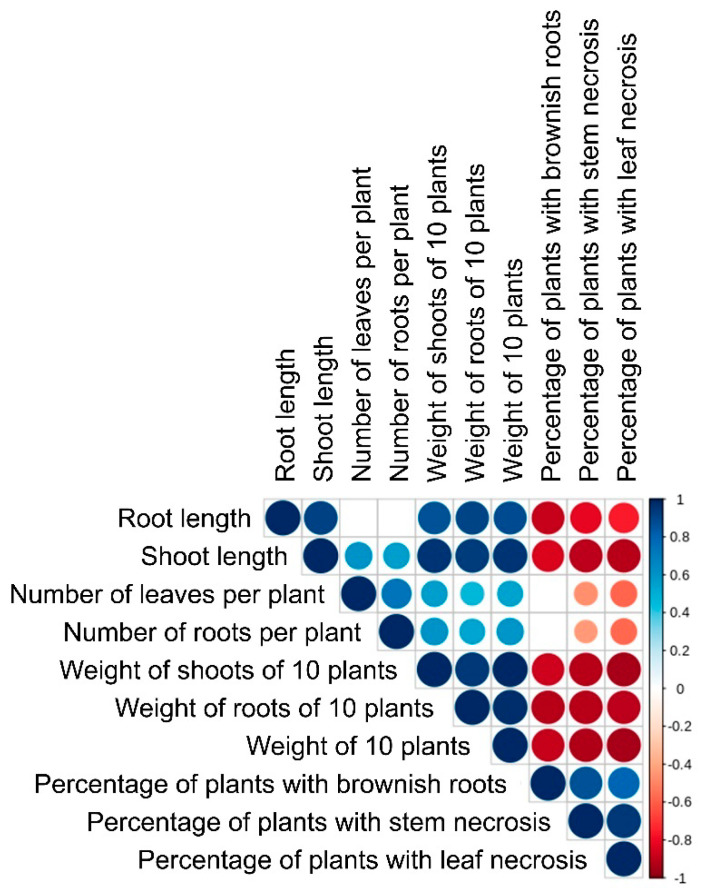
The correlation analysis of different parameters of plants infected by the isolated *Microdochium nivale* strains. The size of a circle and its color corresponds to the value of correlation coefficient. Blue circles—positive correlation; red circles—negative correlation. The scale bar is given to the right. Critical values for Pearson’s correlation coefficient *r* = 0.433.

A statistical analysis was carried out to verify whether there was a correlation between the changes in the assessed parameters of plants during the infection. High positive correlation was found between the pathogen-induced changes in root length and (1) shoot length (0.92), (2) root fresh weight (0.90), and (3) shoot fresh weight (0.86) (Figure 13; Table S20, Supplementary Materials). Moreover, the manifestation of leaf necrosis positively correlated with the manifestation of stem necrosis (0.95) and brownish root pigmentation (0.80). High negative correlation was revealed between the length of roots and (1) percentage of plants with leaf necrosis (−0.75), (2) percentage of plants with stems necrosis (−0.81), and (3) percentage of plants with brownish root pigmentation (−0.75) (Figure 13; Table S20, Supplementary Materials). This means that a greater length and weight of roots and shoots resulted in less manifestation of leaf and stem necrosis and brownish root pigmentation.

**Figure 14:**
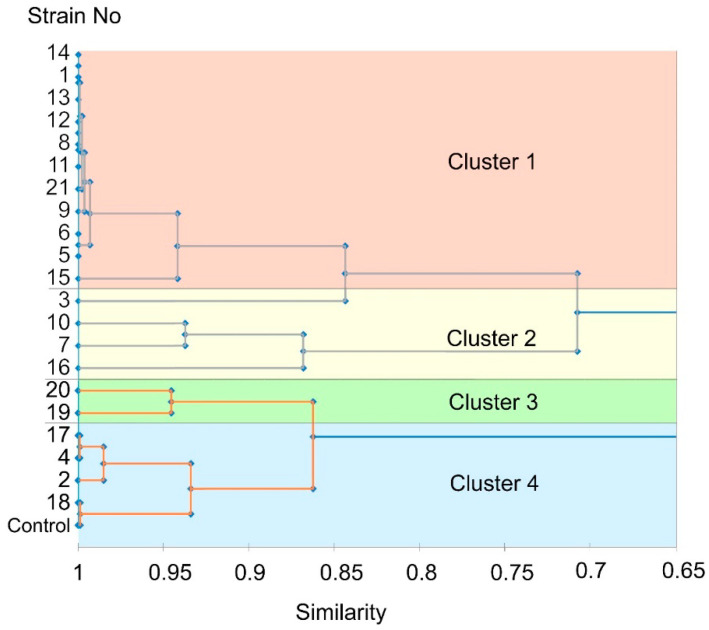
Agglomerative hierarchical clustering of the isolated *Microdochium nivale* strains according to their virulence expressed as the degree of symptom manifestation on the infected rye plants (root length, shoot length, the weight of shoots of 10 plants, the weight of roots of 10 plants, the number of roots, the number of leaves, the percentage of plants with brownish roots, the percentage of plants with stem necrosis, and the percentage of plants with leaf necrosis). The clustering was performed using R, method “complete”. Control—noninfected plants. Clusters were delineated at an arbitrary level of *r* = 0.65.

According to the above-described parameters of the infected plants, we ranged the isolated strains according to the degree of virulence using a multivariate mathematical technique—a cluster analysis. Two main clades were revealed on a dendrogram: the first included strains 1, 3, 5, 6, 7, 8, 9, 10, 11, 12, 13, 14, 15, 16, and 21 (that caused more pronounced disease symptoms), while the second included strains 2, 4, 17, 18, 19, and 20 (that caused less pronounced disease symptoms). Each of the revealed clades branched into two subclades (Figure 14). The first subclade of the first clade included strains 1, 5, 6, 8, 9, 11, 12, 13, 14, 15, and 21—the most virulent strains that caused stem necrosis in 87–100% of the infected plants and leaf necrosis in 47–100% of the infected plants, as well as the most significant reduction in root and shoot length and weight. Therefore, strains 1, 5, 6, 8, 9, 11, 12, 13, 14, 15, and 21 were attributed to the first cluster referred to as “highly virulent strains”. Strain 3 was also included into the first subclade of the first clade. However, this strain formed a separate brunch (Figure 14). This strain led to lesser necrosis (38% and 31% on stems and leaves, respectively) compared to the abovementioned strains and did not cause a reduction in root and shoot length compared to control noninfected plants. Therefore, strain 3 was attributed to cluster 2 “moderately virulent strains” along with strains 7, 10, and 16, which formed the second subclade of the first clade. The strains of the second cluster caused similar, but much less manifested symptoms than the strains of the first cluster (Table 1; Figure S1, Supplementary Materials). Cluster 2 was rather variable in terms of the degree of symptoms caused by different strains of the cluster on the infected plants (Table S21, Supplementary Materials), as reflected on the cluster diagram (Figure 14).

The third cluster “low virulent strains” was formed by two strains (19 and 20) of the first subclade of the second clade (Figure 14). The root length and weight, as well as shoot weight, in the plants infected by these strains significantly differed from the corresponding values of the control plants (Table 1; Table S21, Supplementary Materials). Additionally, these two strains caused leaf and stem necrosis in 7–13% of the infected plants. Lastly, the fourth cluster “avirulent strains” was formed by strains (2, 4, 17, and 18) of the second subclade of the second clade. These strains did not cause stem or leaf necrosis and did not lead to a reduction in root and shoot length or shoot weight compared to the control noninfected plants (Table 1; Table S21, Supplementary Materials). The parameters of the plants infected by strains 2, 4, 17, and 18 clustered together with control noninfected plants (Figure 14).

To check if four assembled clusters indeed statistically differed in the assessed parameters, the ANOVA procedure was performed; variants were compared by the Duncan test. All of the revealed clusters differed in the parameter of root weight (Table 1). The first and second clusters differed in all of the analyzed parameters except for the number of roots and the number of shoots; these two parameters did not differ significantly between any of the assembled clusters. The second and the third clusters differed (in addition to root weight) in the percentage of plants with brownish roots and stem necrosis. The third and the fourth clusters, as well as the fourth cluster and control plants, significantly differed in the root weight (Table 1). In addition, the strains of the fourth cluster did not cause leaf or stem necrosis in contrast to the strains of the third cluster. The obtained clusters are well described according to means of the used indicators (Table 1). Thus, our results show that the four described clusters were assembled correctly and that the root weight is the most precise parameter for monitoring the *M. nivale*-caused infection. Taken together, we ranged the isolated *M. nivale* strains into four categories: highly virulent (1, 5, 6, 8, 9, 11, 12, 13, 14, 15, and 21), moderately virulent (3, 7, 10, and 16), low virulent (19 and 20), and avirulent (2, 4, 17, and 18) strains.

To check if there was a relationship between the degree of virulence of the isolated *M. nivale* strains and their physiological or genetic characteristics (enzymatic activities, growth rate, and attribution to the particular OTU), a correlation analysis was performed. The four above-described virulence groups (highly virulent, moderately virulent, low virulent, and avirulent) were considered. No significant correlation between the degree of virulence and the levels of enzymatic activities or growth rate or attribution to the particular OTU was revealed (Table S20, Supplementary Materials). This means that the degree of virulence of the isolated *M. nivale* strains is not related to the level of a particular enzymatic activity or the growth rate and is not determined by the attribution to a particular OTU.

## 4. Discussion

In the present study we characterized the microbiome of snow mold affected rye plants and isolated the causal agents of this disease—*Microdochium nivale* (*sensu lato*) strains—in order to describe their diversity within a particular area, characterize their potential virulence factors, and compare virulence properties.

### 4.1. Microbiome of Snow Mold-Affected Rye

The microbiome of the snow mold-affected rye plants was characterized differentially for the root endosphere (R-samples), green parts of leaves (GL-samples), and desiccated dead parts of leaves (DL-samples). To the best of our knowledge, the rye microbiome has not been characterized to date except for the rye pollen microbiome [88].

The microbial community richness was the lowest in DL-samples indicating that fewer taxa colonized dead plant tissues compared to live ones. In GL-samples and R-samples, the richness of the fungal community was similar, while it was lower in DL-samples. As for the bacterial community, its richness in R-samples was greater than in GL-samples. The diversity of the fungal community was the greatest in R-samples, while it was similar in GL- and DL-samples. On the other hand, the diversity of bacterial community was similar in R- and GL-samples, while it was lower in DL-samples. The composition of both bacterial and fungal communities differed in R-samples compared to both GL- and DL-samples. Interestingly, although the composition of the bacterial community differed significantly in GL- and DL-samples, the composition of the fungal community was similar in these two sample types. This means that the fungal community underwent fewer alterations compared to the bacterial one as the plant tissues died due to snow mold progression. In the previous studies examining oak leaves, it was shown that both the fungal and the bacterial communities of leaves changed significantly as the leaves died [89,90]. In our case, the fungal community remained relatively stable as the rye leaves collapsed; however, alterations at a species/strain levels cannot be excluded.

In the R-, DL-, and GL-samples together, 15 fungal taxa (13 genera and fungi identified only at the family (Sclerotiniaceae) or order level (Helotiales)) constituted a bulk of the fungal community (genera/taxa with the abundance of ≥10% in at least one of the analyzed samples). The representatives of eight of these 15 taxa (*Microdochium*, *Tetracladium*, *Cistella*, *Phenoliferia*, *Nectria*, *Penicillum*, *Pseudogymnoascus*, Sclerotiniaceae, and Helotiales) were previously described as psychrotolerant fungi [91,92,93,94,95,96,97]. Members of 10 of these taxa (*Microdochium*, *Mycosphaerella*, *Tetracladium*, *Nectria*, *Oculimacula*, *Penicillum*, *Pseudogymnoascus*, *Gibberella*, Sclerotiniaceae, and Helotiales) were previously observed in cereals [19,98,99,100,101,102,103,104,105], and the representatives of three additional genera were described in only noncereal plants: *Leochumicola* (Ericaceae), *Chalara* (woody plants), and *Cistella* (Ericaceae and woody plants) [93,106,107,108,109,110,111]. For two genera (*Lasionectria* and *Phenoliferia*), we did not find information about their presence in plants except for plant remnants [96,112,113,114]. Members of seven of the revealed taxa were previously described as plant pathogens: *Microdochium*, *Mycosphaerella* (many different plant diseases) [19,99,115], *Nectria* (canker, twig blight, coral spot in woody plants, especially fruit trees, and orchards) [92], *Oculimacula* (eyespot disease of cereals) [101,116], *Gibberella* (many different plant diseases) [100], *Chalara* (ash dieback of woody plants) [106,107], and Sclerotiniaceae (many different plant diseases, including the snow mold) [102,117]. Plant endophytic fungi were previously described for seven of the revealed taxa: *Tetracladium* (typical root endophyte of winter wheat) [103,105], *Cistella* (ericoid mycorrhizal fungi) [93], *Penicillum* (endophytes of arctic plants) [118], *Pseudogymnoascus* (endophytes of boreal plants) [91], *Leochumicola* (ericoid mycorrhizal fungi) [119], and Helotiales (mycorrhizal fungi of different plants including cereals) [93,120]. Representatives of two of the revealed genera *Tetracladium* (*T. maxilliforme*) and *Oculimacula* (*O. yallundae*) were previously revealed in plants together with *M. nivale*. Furthermore, *O. yallundae*—a causative agent of the eyespot disease—was proposed to antagonize *M. nivale* [105].

*Mycosphaerella* and *Microdochium* genera strongly dominated in leaf samples (DL- and GL-samples) of winter rye 10 days after the snowmelt. The dominance of *Microdochium* was not a surprise since strong progression of the snow mold on the analyzed plants was observed. However, the leaf spot lesions caused by *Mycosphaerella* [121,122] were visually much less evident in spite of the fact that this genus was even more represented in rye leaves than *Microdochium*. Presumably, the conditions of an early spring did not promote the aggressive behavior of non-psychrotolerant *Mycosphaerella*, while *Microdochium* could fully realize its pathogenic potential. In DL-samples, the genera *Cistella*, *Lasionectria*, *Oculimacula*, *Penicillum*, and *Phenoliferia* were more represented than in GL-samples, and *Nectria* was revealed in DL-samples only. On the other hand, *Tetracladium* and Sclerotiniaceae were more represented in GL-samples compared to DL-samples. In the R-samples compared to GL-samples, the portions of *Mycosphaerella*, Sclerotiniaceae, and *Phenoliferia* were reduced. Moreover, the portion of *Pseudogymnoascus* that was a dominant in the R-samples, as well as *Penicillum* and Helotiales, was increased in the R-samples compared to GL-samples. The members of *Chalara*, *Gibberella*, and *Leochumicola* were revealed in R-samples, but not in GL- or DL-samples. In general, the taxa for which psychrotolerant fungi have been described were more represented in R-samples compared to GL- and DL-samples.

Three genetic variants of *Microdochium* were revealed in rye microbiome referred to as M.OTU1 (*M. nivale sensu lato*), M.OTU2 (*M. nivale sensu stricto*), and M.OTU3 (*M. bolleyi*). M.OTU1 and M.OTU2 were revealed in all sample types, while M.OTU3 was revealed only in R-samples. Interestingly, in the GL-samples, M.OTU1 and M.OTU2 were represented rather equally, while, in the DL-samples, M.OTU1 was strongly dominant. This means that the strains belonging to M.OTU1 have an advantage over the representatives of M.OTU2 as the host plant tissues collapse, indicating that the representatives of M.OTU1 are likely to utilize more necrotrophic lifestyle compared to more biotrophic one of the representatives of M.OTU2.

Thirteen bacterial taxa (12 genera and bacteria of the family Micobacteriaceae) constituted a bulk of the bacterial community of winter rye (R-, DL-, and GL-samples together). The representatives of three of these 13 taxa (*Cryobacterium*, *Polaromonas*, and *Enhydrobacter*) were previously described as psychrotolerant bacteria [123,124,125]. Typical plant pathogenic bacteria were not revealed except for the *Pseudomonas* and *Rhodococcus* genera which are almost ubiquitous and contain plant pathogens, plant endophytes, plant protecting bacteria, and free-living bacteria [126,127,128,129,130]. The members of 11 of the 13 revealed bacterial taxa were previously described to be associated with cereals: nine taxa were shown to colonize plant endosphere (*Rhizobium*, *Pedobacter*, *Cryobacterium*, *Polaromonas*, *Arthrobacter*, *Pseudomonas*, *Rhodococcus*, *Janthinobacterium*, and Microbacteriaceae) and two genera were described as rhizosphere/phylosphere inhabitors (*Mycobacterium* and *Propionibacterium*) [131,132,133,134,135]. Two taxa (*Promicromonospora* and *Enhydrobacter*) were not previously shown to be associated with cereals, but were revealed in rhizosphere of noncereal plants [136,137,138,139,140].

Six of the revealed bacterial taxa (*Rhizobium*, *Pseudomonas, Polaromonas*, *Mycobacterium*, *Arthrobacter*, and Microbacteriaceae) include diazotrophic bacteria. *Rhizobium* and *Polaromonas* were revealed only in roots, and *Mycobacterium* was predominantly found in roots. Additionally, non-diasotrophic genus *Promicromonospora* was detected in roots but not in DL- or GL-samples. As for the leaf samples, *Pseudomonas* and *Pedobacter* were the dominant genera in both DL- and GL-samples; the latter was more represented in GL-samples compared to DL-samples. *Enhydrobacter* and *Propionibacterium* were present in DL-samples but not in GL-samples and roots, indicating that the representatives of these genera utilize a saprotrophic mode of existence. The portion of *Janthinobacterium* and Microbacteriaceae was increased in GL-samples compared to DL-samples, while *Rhodococcus* was more represented in DL-samples compared to GL-samples. Although we did not observe the dramatic differences in the composition of bacterial genera in DL- and GL-samples, NMDS analysis showed that the bacterial communities in these two sample types significantly differed. This likely means that visually healthy leaves and dead leaves are colonized by different varieties (species/strains having different ASV variants) of similar genera.

### 4.2. Microdochium nivale Strains Associated with Rye Snow Mold: Morphology, Genetics, and Enzymatic Activities

Twenty-one strains (referred to as 1–21) preliminary attributed to the *Microdochium* genus on the basis of mycelium morphology were isolated from winter rye. The strains differed in morphology and growth rate. Seven strains formed conidia typical of *M. nivale* [17]. Sequencing of the ITS2 region confirmed that all 21 strains belonged to *Microdochium* genus. The isolated strains were attributed to four different OTUs: M.OTU1, M.OTU2, M.OTU4, and M.OTU5. M.OTU3 that was revealed as a minor OTU among the OTUs that belonged to *Microdochium* in the microbiome of rye was not identified among the isolated strains. According to the performed analysis of specie-specific nucleotide positions, M.OTU1 and M.OTU4 were attributed to *M. nivale sensu lato*, while M.OTU2 and M.OTU5 were attributed to *M. nivale sensu stricto*. M.OTU3 belonged to *M. bolleyi* that does not cause snow mold disease.

Plant pathogenic microorganisms attack their host plants by using extracellular enzymes, mostly PCWDEs. However, the enzymes that are considered as major PCWDEs (cellulases, xylanases, pectinases, proteases, and lignin peroxidases) have not been analyzed for the members of *Microdochium* genus. The only extracellular enzymatic activities described for *Microdochium* species are amylase and invertase (non-PCWDEs) and glucosidase and galactosidase (“minor” PCWDEs) [44,74,141]. In our study, we showed that, in addition to extracellular amylase and invertase, *Microdochium* strains produce extracellular cellulase (endoglucanase), protease, lignin peroxidase, xylanase, arabinofuranosidase, and pectate lyase. Extracellular lignin peroxidase, amylase, cellulase (endoglucanase), and protease activities were revealed in all of the analyzed strains; the other types of activities were absent in some of the strains under the experimental conditions.

The levels of the analyzed activities significantly varied across different strains from threefold (for lignin peroxidase) to 376-fold (for cellulase). Such variability in the activity of the particular enzymes in different strains of a single species was previously described for plant pathogenic fungi [44,142]. Arabinofuranosidase activity was revealed in trace amounts in almost all of the isolated strains. In general, the levels of most analyzed activities (cellulase (endoglucanase), xylanase, pectate lyase, invertase, and amylase) in *M. nivale* strains were more or less comparable to those determined previously for other ascomycetous plant pathogenic or plant-associated fungi [44,143,144,145,146,147,148]. Furthermore, the extracellular lignin peroxidase activity of *M. nivale* strains was greater than in *Paraconiothyrium variabile*, *Aspergillus flavus*, and *Emericella nidulans* [149,150], while arabinofuranosidase and protease activities were lower than in other analyzed ascomycetous plant pathogenic fungi [151,152,153,154,155,156].

Our study showed that *M. nivale* strains produce a cocktail of extracellular enzymes, including PCWDEs, which may serve as virulence determinants of the studied fungi. However, the role of these enzymes in *M. nivale*-caused pathogenesis needs further investigation. The establishment of the role of a particular enzyme in the virulence of plant pathogenic fungi is a challenge because of the redundancy of PCWDEs in fungi, which hampers defining their biological functions. In some of the cases, single mutations caused a reduction in virulence, e.g., the knockout of subtilisin-like protease in *Botrytis cinerea* [157], endo-β-1,4-xylanase in *B. cinerea* and *Valsa mali* [158,159], and lignin peroxidase in *Verticillium nonalfalfae* [160]. However, often, the knockout of a single PCWDE gene has no effect on virulence of plant pathogenic fungi. The targeted deletion of one or even two protease genes failed to change virulence of the plant pathogenic fungi *F. oxysporum*, *Glomerella cingulata*, and *B. cinerea* [161,162,163]. Disruption of single endoxylanase genes in a number of plant pathogenic fungi also did not reduce their virulence [164,165,166,167,168,169,170,171]. The same was true for single mutations in pectate lyase genes [172,173]. However, when two pectate lyase genes were knocked out, the virulence of mutant *F. solani* was drastically reduced compared to the wildtype or single mutants [172]. Similarly, a double (but not single) protease mutant of *F. oxysporum* had reduced virulence [174]. In addition, different PCWDEs are likely to “support each other” in the decomposition of the plant cell wall, and the deficiency in one of the activities seems to be complemented by the other(s). Single mutations in either xylanase or polygalacturunase genes did not affect the virulence of *F. graminearum*, whereas a double mutation in both genes significantly reduced the virulence of the pathogen [175].

Given the synergistic action of different fungal PCWDEs, we presumed that correlation between the levels of activities of different extracellular enzymes in *M. nivale* strains may exist. However, we did not find any correlations in the enzyme activities. Moreover, each strain appeared to be characterized by a unique pattern (at least in quantitative terms) of extracellular enzymatic activities. Thus, our results show that, although 21 analyzed strains fell into only four OTUs (according to 164 bp ITS2 fragment), each particular strain presumably had a unique genotype expressed as variation in the observed morphological traits, growth rates, and extracellular enzymatic activities. Further deeper genome characteristics may shed light on the genetic basis of the observed morphophysiological variability of the isolated *M. nivale* strains.

### 4.3. Virulence of Microdochium nivale Strains

The isolated *M. nivale* strains caused a reduction in the length and weight of roots and shoots, as well as the manifestation of brownish color on roots and necrosis on stems and leaves, of the infected plants. The degree of symptoms caused by different strains varied, consistent with differential virulence of *M. nivale* strains reported in previous studies [41,48,49,52,176]. Most of the parameters of plants infected by different strains had strong positive or negative correlations. The root length, shoot length, root fresh weight, and shoot fresh weight correlated positively. Manifestation of leaf and stem necrosis and brownish root pigmentation also had positive correlation. On the other hand, the length and weight of shoots and roots correlated negatively with leaf and stem necrosis and brownish root pigmentation. The number of leaves or roots did not have strong correlation with any of the assessed parameters.

Both visual inspection and statistical analysis showed that the root system of the infected plants suffered more substantially from *M. nivale* strains compared to the aboveground plant parts, and the root weight appeared to be the most precise parameter for monitoring the *M. nivale* caused disease. Such “susceptibility” of the root system to *M. nivale*-caused infection has not been described previously. Whether the “susceptibility” of the root system is due to its extensive colonization by the *M. nivale* strains or is determined by the disturbance in plant correlative growth because of the extensive colonization of the aboveground plant parts remains to be determined. The latter possibility seems to be more valid since we observed the surface mycelium only on the aboveground plant parts but not on the roots; however, the obligatory endophytic growth of *M. nivale* mycelium inside the roots cannot be excluded.

To characterize the isolated strains according to the degree of virulence, the assessed parameters of the infected plants were analyzed using a multivariate mathematical technique—cluster analysis. The measure of similarities/differences between the strains for the entire set of the studied traits (parameters of the infected plants) was determined and visualized in the form of a dendrogram within which the most similar strains were grouped in a cluster. Four clusters were assembled: highly virulent strains, moderately virulent strains, low virulent strains, and avirulent strains. One-way analysis of variance followed by Duncan’s test for multiple comparisons of means confirmed that the clusters significantly differed from each other and, thus, were assembled correctly.

Given that the isolated *M. nivale* strains differed in the degree of virulence, we tried to elucidate these differences in terms of the activity of the particular extracellular enzymes, growth rate, or attribution to a particular OTU. However, we did not reveal correlations between the degree of virulence and the levels of enzymatic activities, growth rates, or attributions to particular OTUs. The absence of a correlation between the level of enzymatic activity of virulence factors and virulence was previously observed for plant pathogenic fungi [142,177]. On the other hand, correlation between virulence and glucosidase activity was revealed for *M. nivale* and *M. majus* [44]. However, it should be noted that glucosidases are not described as crucial virulence factors or major PCWDEs in contrast to cellulases, xylanases, pectate lyases, proteases, and lignin peroxidases that were analyzed in our study.

Thus, in order to cause the disease, each of the highly virulent *M. nivale* strains seems to use its own, unique strategy involving “a personal” pattern (at least in quantitative terms) of extracellular enzymatic activities. The strains that cause similar (in both quantitative and qualitative terms) disease symptoms are likely to rely on different enzymatic activities. For example, highly virulent strains 6, 11, and 21 are likely to give priority to cellulase, but produce a low level of extracellular xylanase. All other highly virulent strains produce much less cellulase, but display a high level of extracellular xylanase (highly virulent strains 5, 8, 14, and 15), amylase (highly virulent strains 1, 5, 8, 9, 14, and 21), or invertase (highly virulent strains 5, 8, and 21) activity. Highly virulent strains 12 and 13 were absent among the “leaders” in the production of any of the analyzed enzymatic activities. Given that all of the analyzed strains inhabit a common area, it can be speculated that they may act synergistically. Thus, each strain implements the particular functions and contributes in its own way to an integrated pool of extracellular enzymes necessary for disease progression.

## 5. Conclusions

Genetically and phenotypically diverse *Microdochium nivale sensu lato* strains were found to colonize winter rye plants within a common area. The diversity of the microbiome was greater in the live parts of snow mold-affected rye plants compared to dead parts. Pink snow mold-affected winter rye plants were heavily colonized (in addition to *M. nivale*) by *Mycospherella* species. One of the genetic groups of *M. nivale sensu lato* (M.OTU1) was dominant as host plant tissues collapsed, indicating that representatives of this group are characterized by a more saprotrophic mode of action. Distinct strains of *M. nivale sensu lato* (inhabiting a common area and particular host plant—winter rye) have different morphology and growth rates and are characterized by different patterns (at least in quantitative terms) of extracellular enzymatic activities, including PCWDE activities. No correlations existed between levels of different enzymatic activities or activity levels and growth rate. Isolated *M. nivale sensu lato* strains were characterized by different degrees of virulence: highly virulent, moderately virulent, low virulent, and avirulent. The root system of the rye plants was more susceptible to *M. nivale*-caused infection than the aboveground plant parts despite no visible surface mycelium being observed on roots in contrast to the aboveground parts. Root weight was the most precise parameter for monitoring *M. nivale*-caused infection. No correlation was revealed between the degree of virulence of isolated strains and the levels of analyzed enzymatic activities, growth rates, or particular OTU attributes. Consequently, highly virulent strains that cause similar disease symptoms varied in the levels of different extracellular enzymatic activities and, thus, may be likely to use their own, unique strategy of causing disease. This knowledge may accelerate the breeding process by improving and standardizing screening methods for the identification of new and effective resistance sources and may support efficient and sustainable cultivation of winter cereals in areas of higher latitude such as Russia and Canada.

## Figures and Tables

**Table 1:** Mean values of the assessed parameters of the rye plants infected by the isolated *Microdochium nivale* strains of different clusters: 1. highly virulent, 2. moderately virulent, 3. low virulent, and 4. avirulent. The following parameters of the plants were analyzed: number of roots, number of leaves, root length, shoot length, the weight of roots of 10 plants, the weight of shoots of 10 plants, the weight of 10 plants, the percentage of plants with brownish roots, the percentage of plants with stem necrosis, and the percentage of plants with leaf necrosis. Clusters that do not share the same letter (given in brackets) have significantly different means in pairwise comparison (Duncan’s multiple range test).

**Cluster**	**Strain No**	**Number of Roots**	**Number of Leaves**	**Root Length (cm)**	**Shoot Length (cm)**	**Root Weight (g)**	**Shoot Weight (g)**	**Plant Weight (g)**
1. Highly virulent	1, 5, 6, 8, 9, 11, 12, 13, 14, 15, 21	7.0 (a)	3.1 (a)	4.4 (a)	21.4 (a)	0.4 (a)	1.5 (a)	1.9 (a)
2. Moderately virulent	3, 7, 10, 16	7.9 (a)	3.6 (a)	7.8 (b)	32.5 (b)	1.2 (b)	4.0 (b)	5.2 (b)
3. Low virulent	19, 20	7.9 (a)	3.5 (a)	8.8 (bc)	34.3 (bc)	1.5 (c)	4.6 (bc)	6.1 (bc)
4. Avirulent	2, 4, 17, 18	7.6 (a)	3.5 (a)	10.4 (cd)	35.7 (bc)	1.7 (d)	5.0 (cd)	6.7 (c)
Control plants		6.8 (a)	4.2 (a)	12.3 (d)	37.8 (c)	2.3 (e)	5.8 (d)	8.1 (d)
**Cluster**	**Strain No**		**% of Plants with** **Brownish Roots**		**% of Plants with Stem Necrosis**		**% of Plants with Leaf Necrosis**	
1. Highly virulent	1, 5, 6, 8, 9, 11, 12, 13, 14, 15, 21 (c)		99.4		98.8 (c)		89.3 (b)	
2. Moderately virulent	3, 7, 10, 16 (b)		72.8		32.8 (b)		20.0 (a)	
3. Low virulent	19, 20 (a)		19.5		10.0 (a)		10.0 (a)	
4. Avirulent	2, 4, 17, 18 (a)		8.8		0 (a)		0 (a)	
Control plants	(a)		0		0 (a)		0 (a)	

## Supplementary Materials

The following are available online at https://www.mdpi.com/2309-608X/6/4/335/s1: Figure S1. Photos of the rye plants infected with the isolated strains of *Microdochium nivale* (20 dpi); Table S1. Number of reads corresponding to ITS2 region of fungal rRNA locus in the analyzed samples of snow mold damaged winter rye plants; Table S2. Number of reads corresponding to V4 region of the bacterial 16S rRNA gene in the analyzed samples of snow mold damaged winter rye plants; Table S3. Absolute abundance (number of reads corresponding to ITS2 region of fungal rRNA locus) of fungal OTUs in the samples of winter rye plants; Table S4. Percentage abundance of fungal OTUs in the samples of winter rye plants; Table S5. Absolute abundance (number of reads corresponding to V4 region of 16S rRNA gene) of bacterial ASVs in the samples of winter rye plants; Table S6. Percentage abundance of bacterial ASVs in the samples of winter rye plants; Table S7. The percentage of reads related to the fungal genera revealed as the most abundant in winter rye plants; Table S8. The percentage of reads related to the bacterial classes revealed as the most abundant in winter rye plants; Table S9. The percentage of reads related to the bacterial genera revealed as the most abundant in winter rye plants; Table S10. Absolute abundance (upper table, number of reads) and percentage abundance (downer table) of *Microdochium*-related OTUs revealed in the fungal community of snow mold-damaged winter rye plants; Table S11. Collection IDs of the isolated strains of *Microdochium nivale*; Table S12. Morphological characteristics of the isolated *Microdochium nivale* strains after 14 days of cultivation on potato sucrose agar; Table S13. Morphology of the conidia of the isolated *Microdochium nivale* strains; Table S14. Attribution of the isolated *Microdochium nivale* strains to the particular OTUs; Table S15. IDs of the sequences having the highest levels of identity and coverage with the 164 bp fragment of ITS2 of M.OTU1; Table S16. IDs of the sequences having the highest levels of identity and coverage with the 164 bp fragment of ITS2 of M.OTU2; Table S17. IDs of the sequences having the highest levels of identity and coverage with the 164 bp fragment of ITS2 of M.OTU3; Table S18. IDs of the sequences having the highest levels of identity and coverage with the 164 bp fragment of ITS2 of M.OTU4; Table S19. IDs of the sequences having the highest levels of identity and coverage with the 164 bp fragment of ITS2 of M.OTU5; Table S20. Correlation analysis of enzymatic activities and growth rate of the isolated *Microdochium nivale* strains and morphological characteristics of plants infected by the isolated strains; Table S21. Agglomerative hierarchical clustering of the isolated *Microdochium nivale* strains according to the degree of symptom manifestation on the infected rye plants after the infection.
