# Supplementary material for: Rye Snow Mold-Associated Microdochium nivale Strains Inhabiting a Common Area: Variability in Genetics, Morphotype, Extracellular Enzymatic Activities, and Virulence
Source: J Fungi (Basel). 2020 Dec 3;6(4):335. doi: 10.3390/jof6040335 (PMC7761817; doi:10.3390/jof6040335)

Control, non-infected rye plants

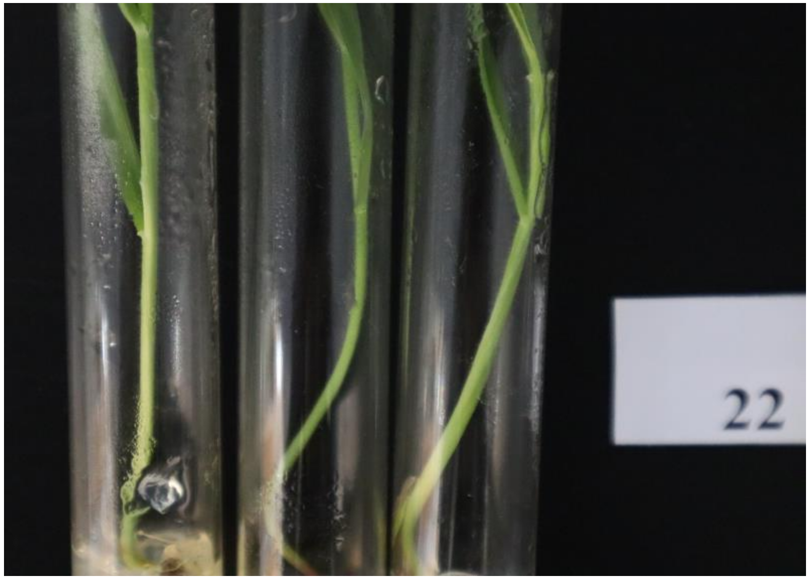

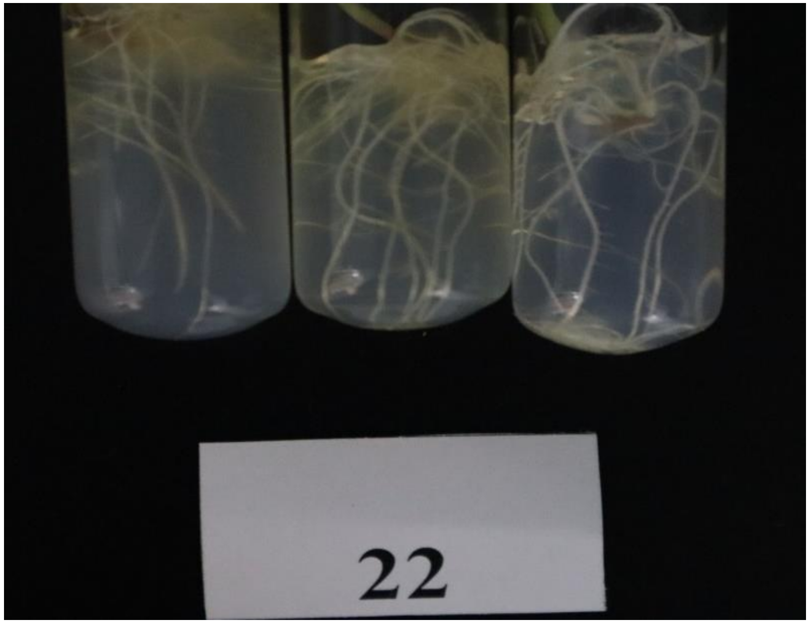

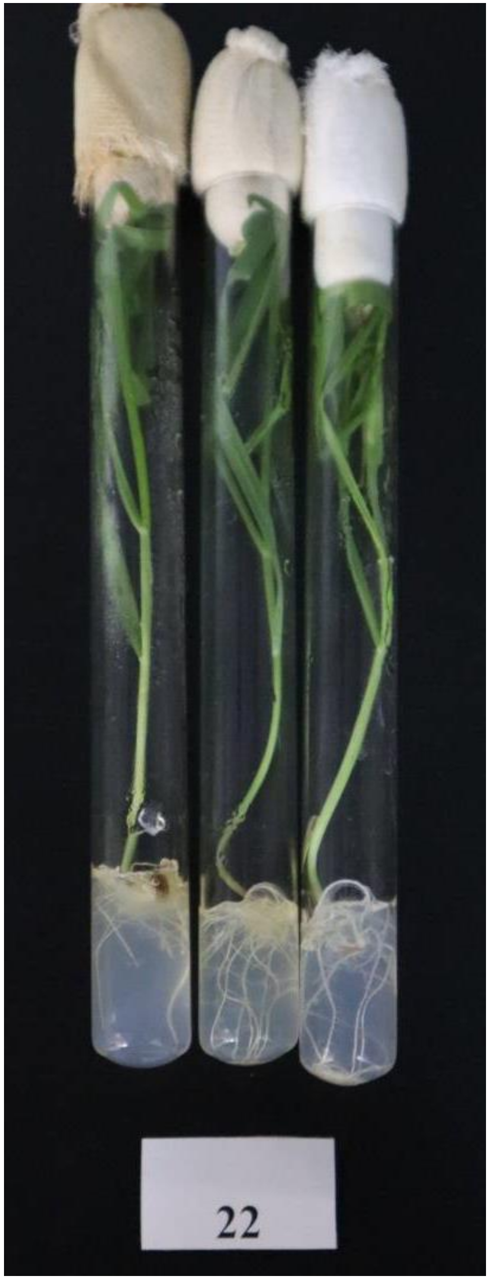

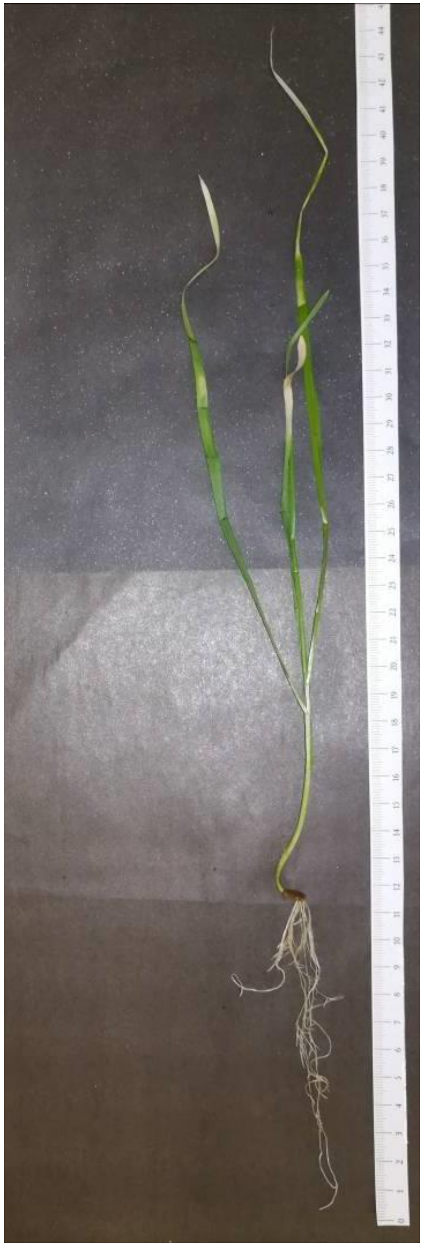

Rye plants infected by *M. nivale* strain 1 (20 dpi)

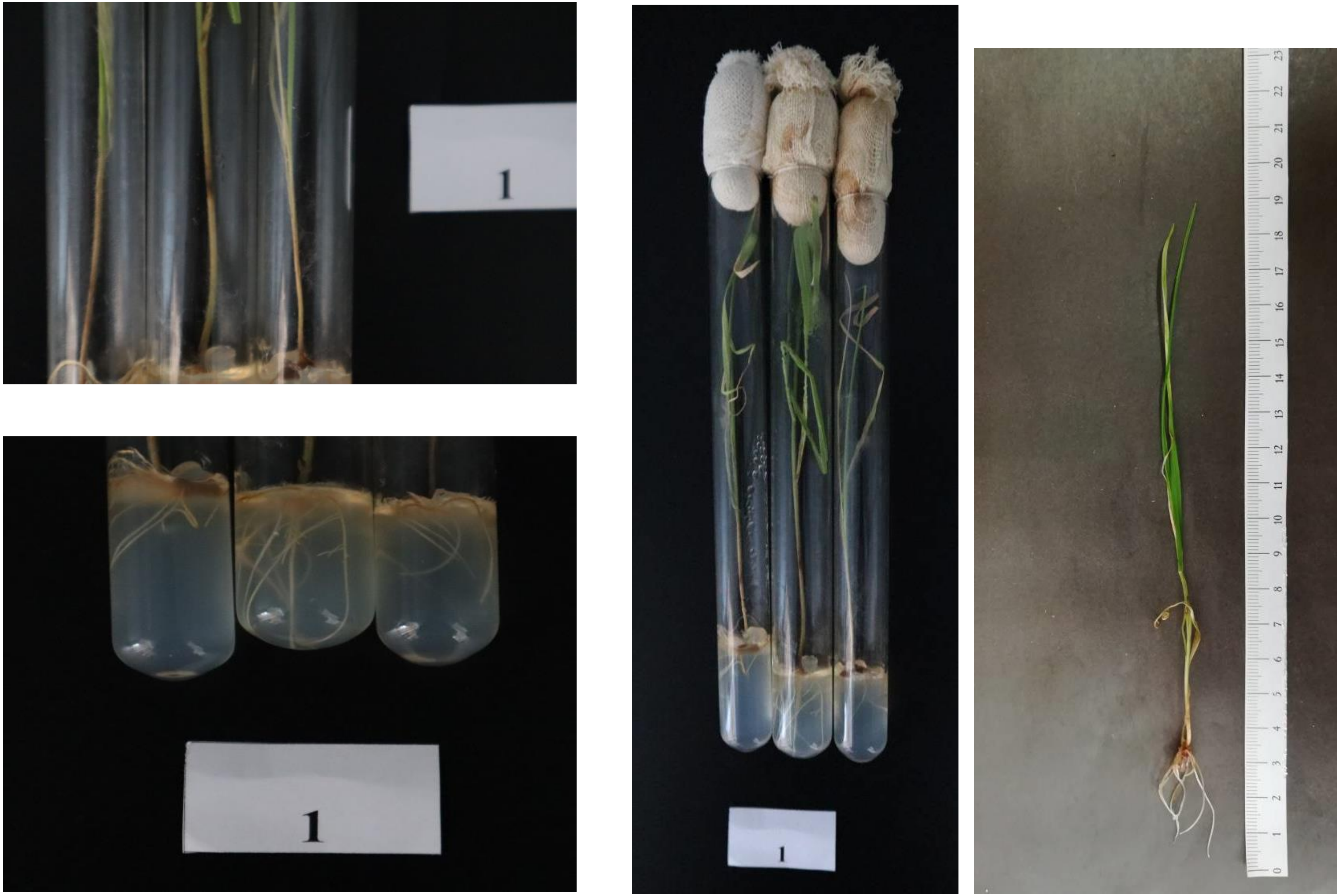

Rye plants infected by *M. nivale* strain 2 (20 dpi)

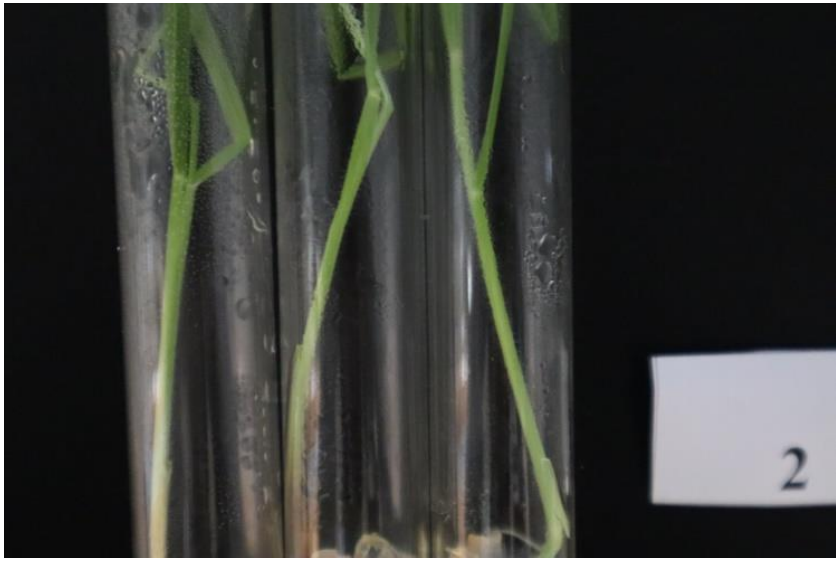

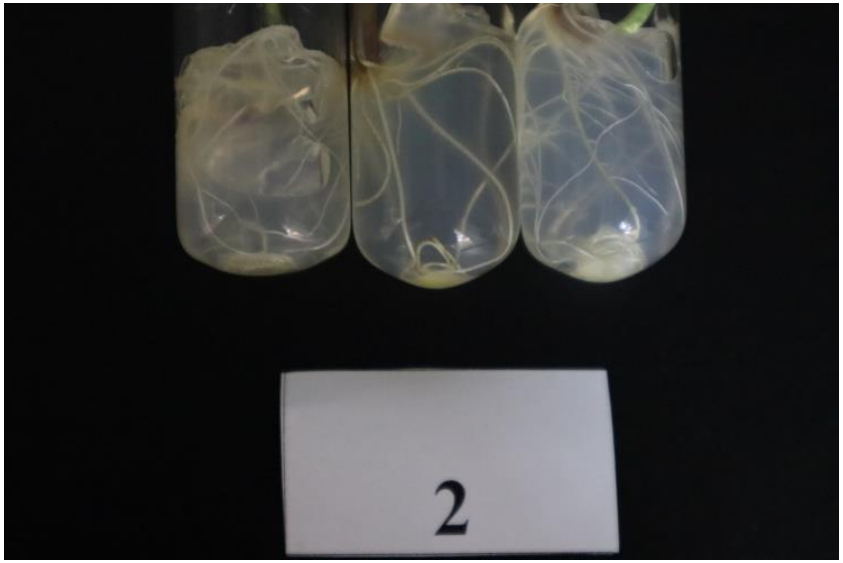

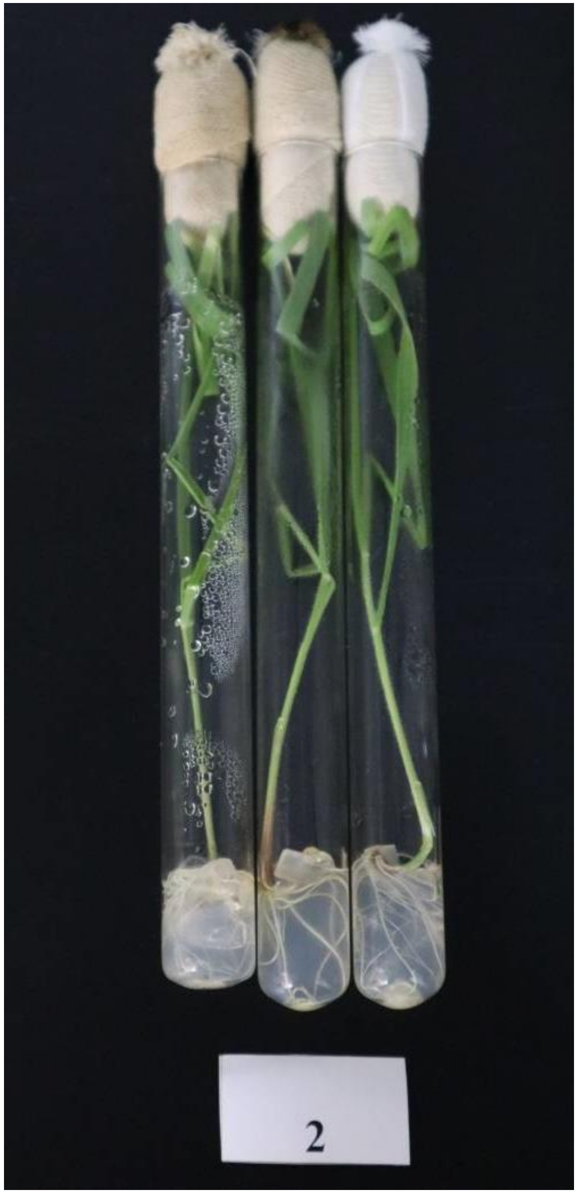

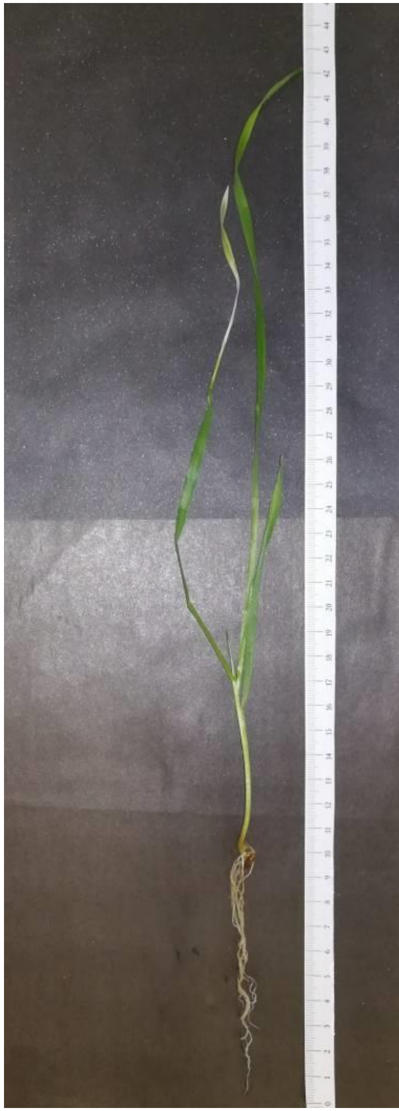

Rye plants infected by *M. nivale* strain 3 (20 dpi)

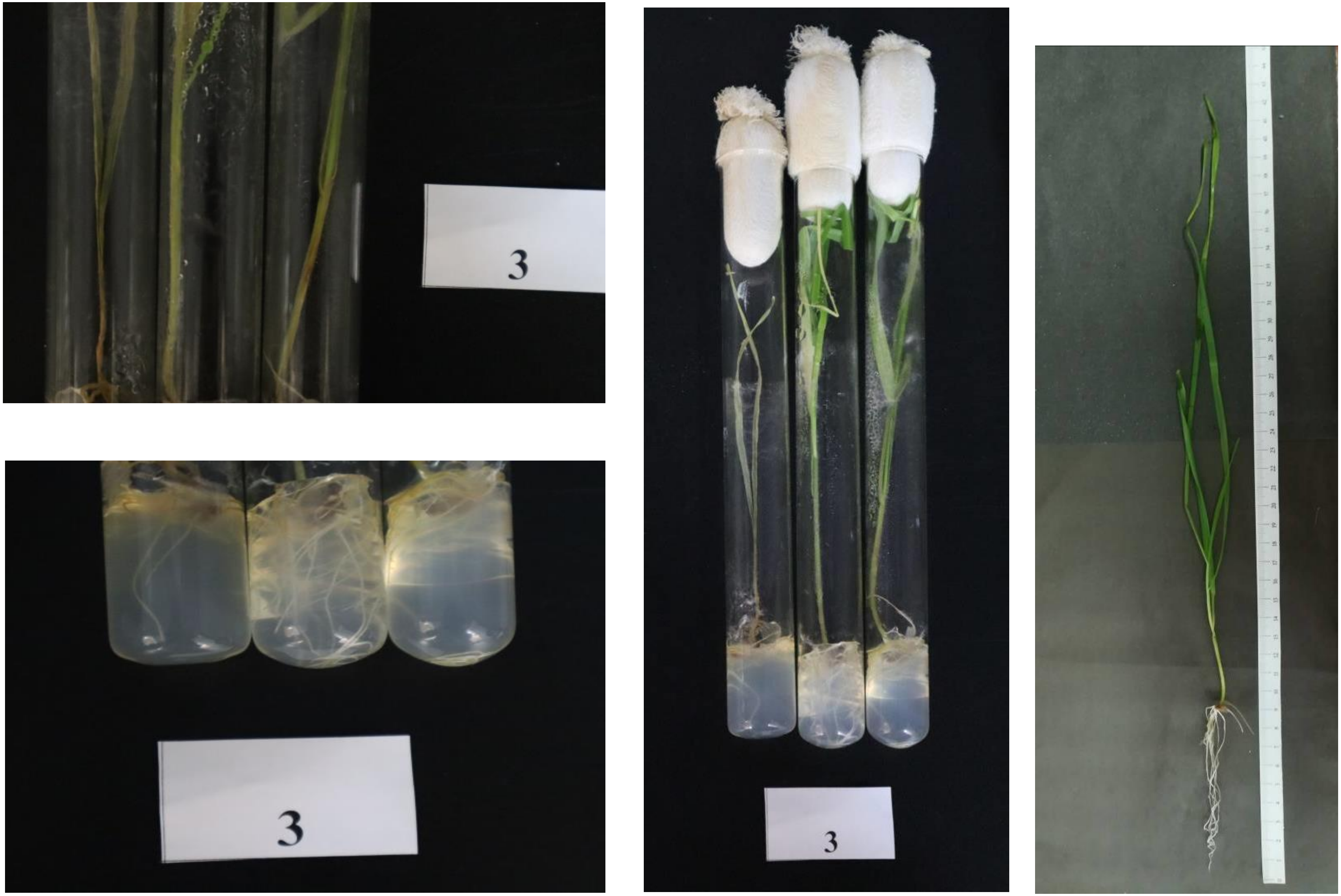

Rye plants infected by *M. nivale* strain 4 (20 dpi)

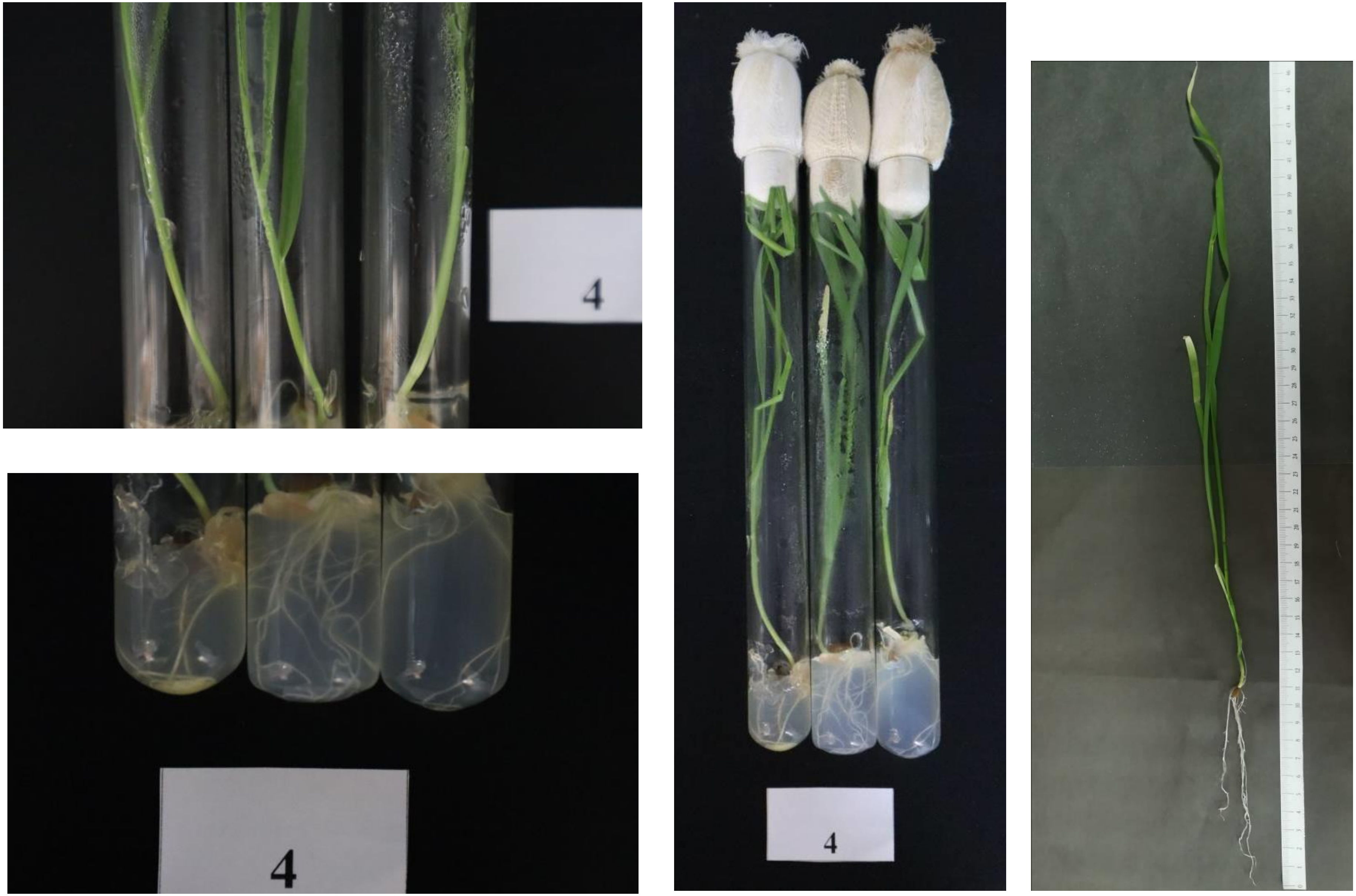

Rye plants infected by *M. nivale* strain 5 (20 dpi)

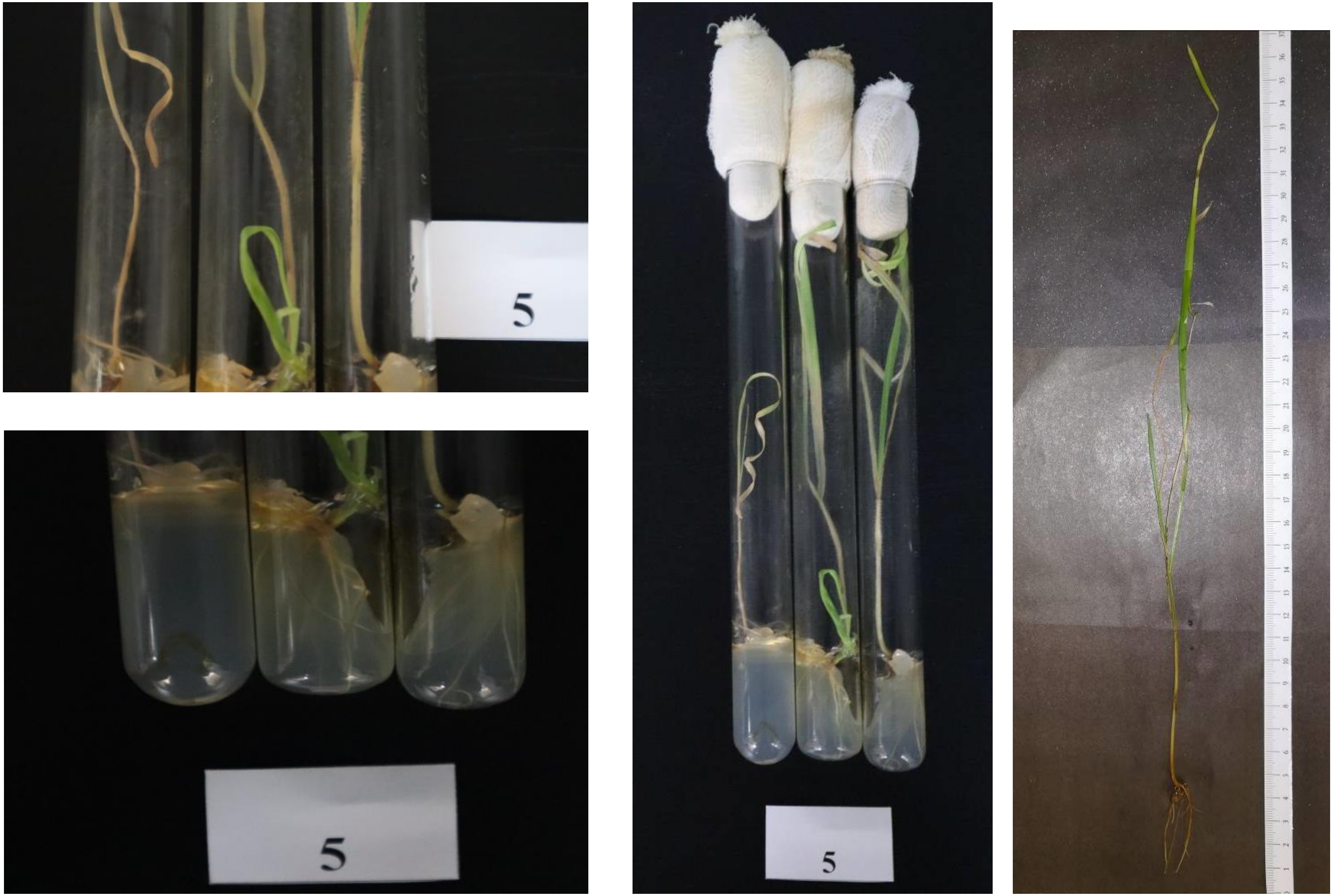

Rye plants infected by *M. nivale* strain 6 (20 dpi)

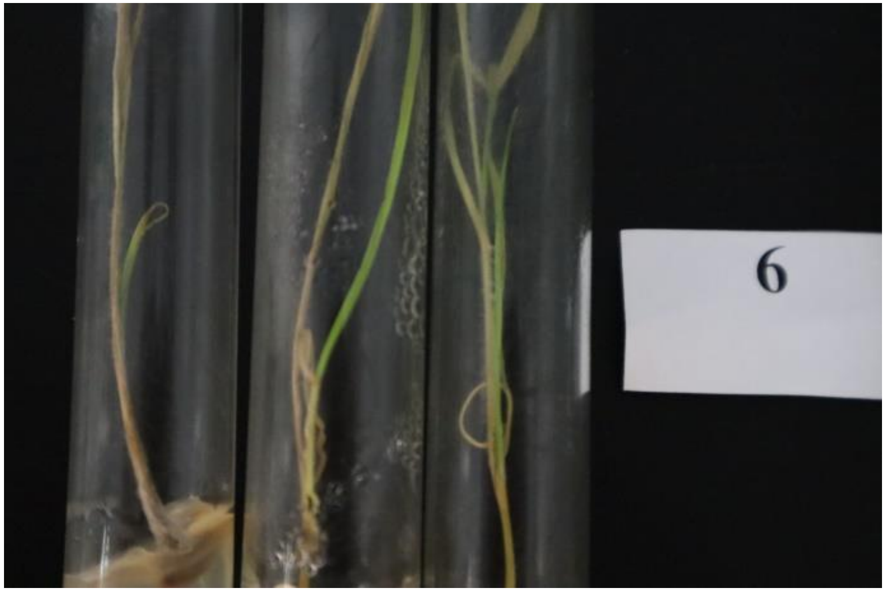

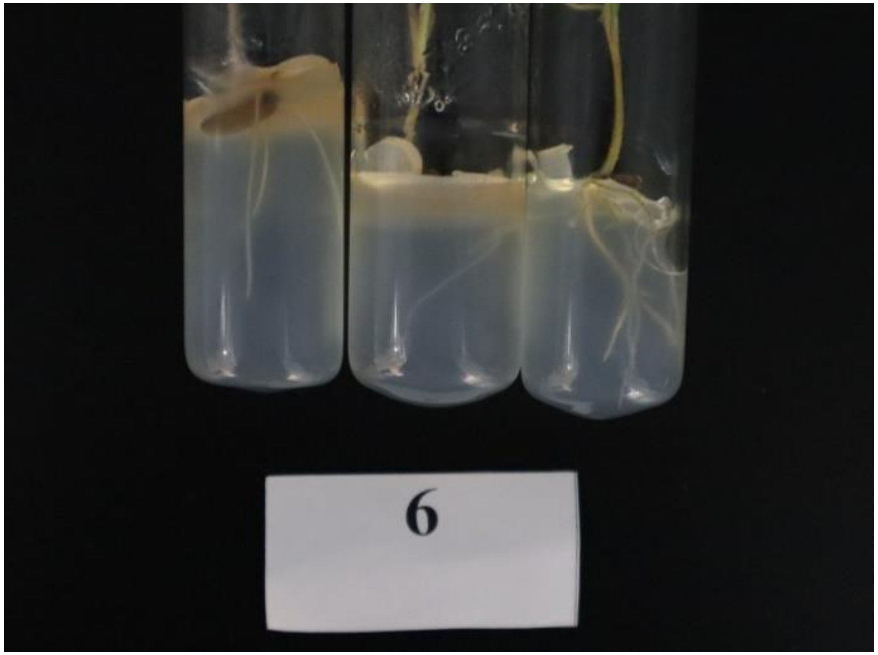

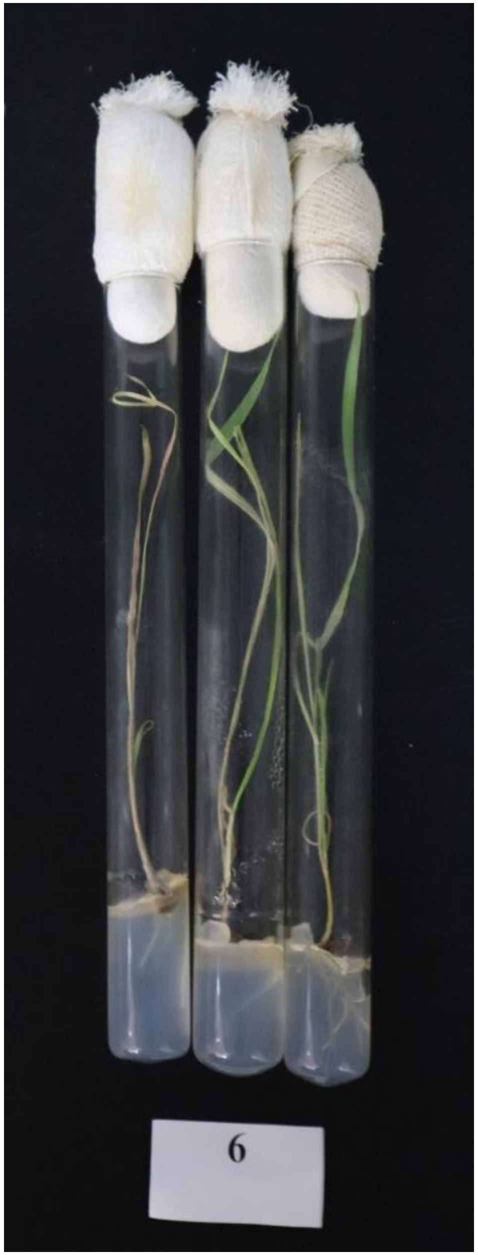

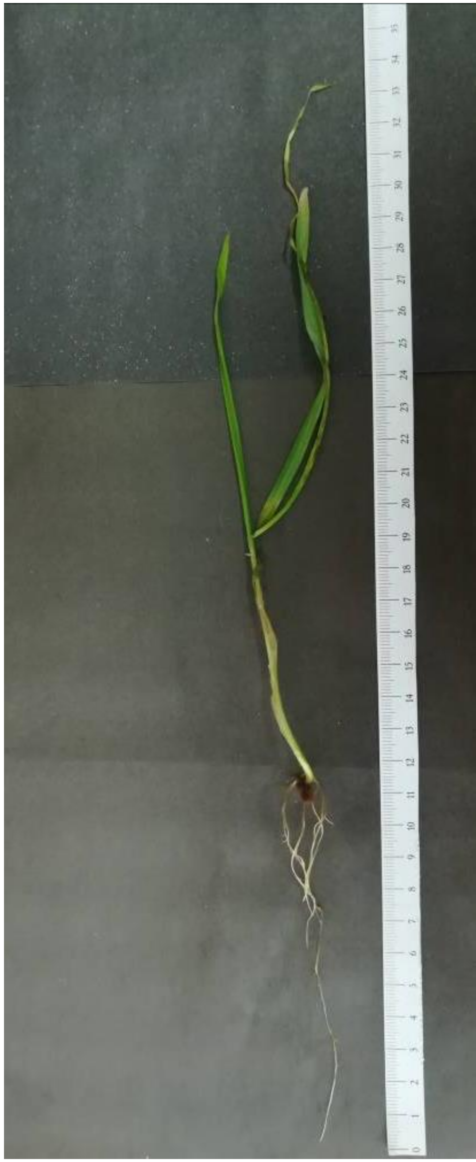

Rye plants infected by *M. nivale* strain 7 (20 dpi)

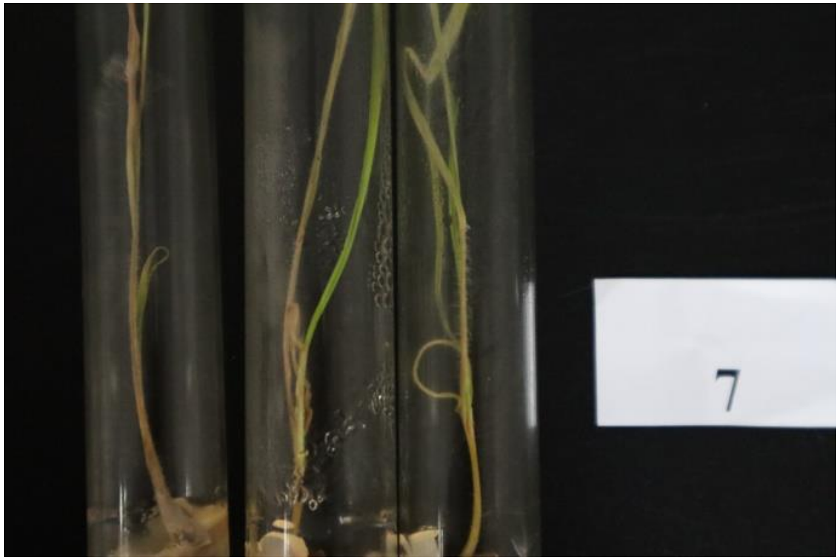

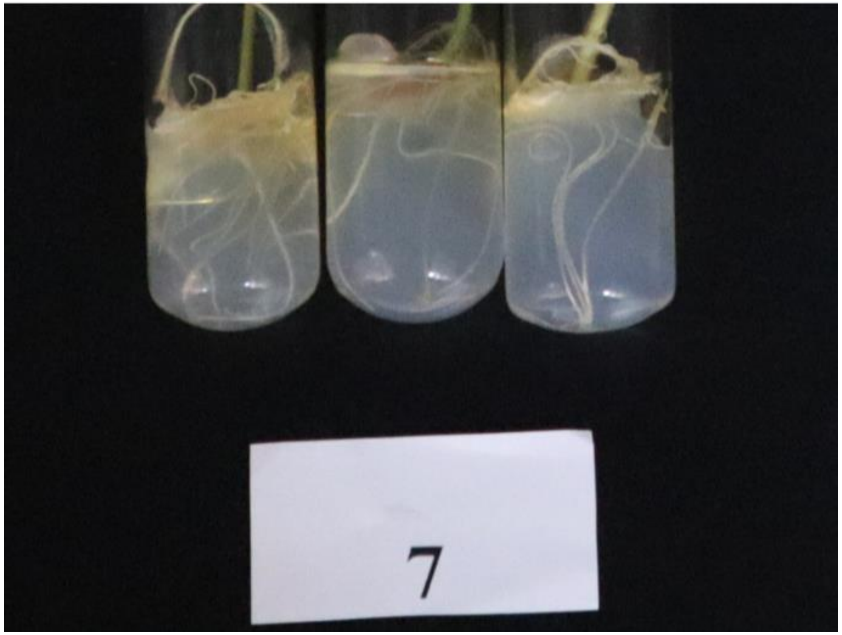

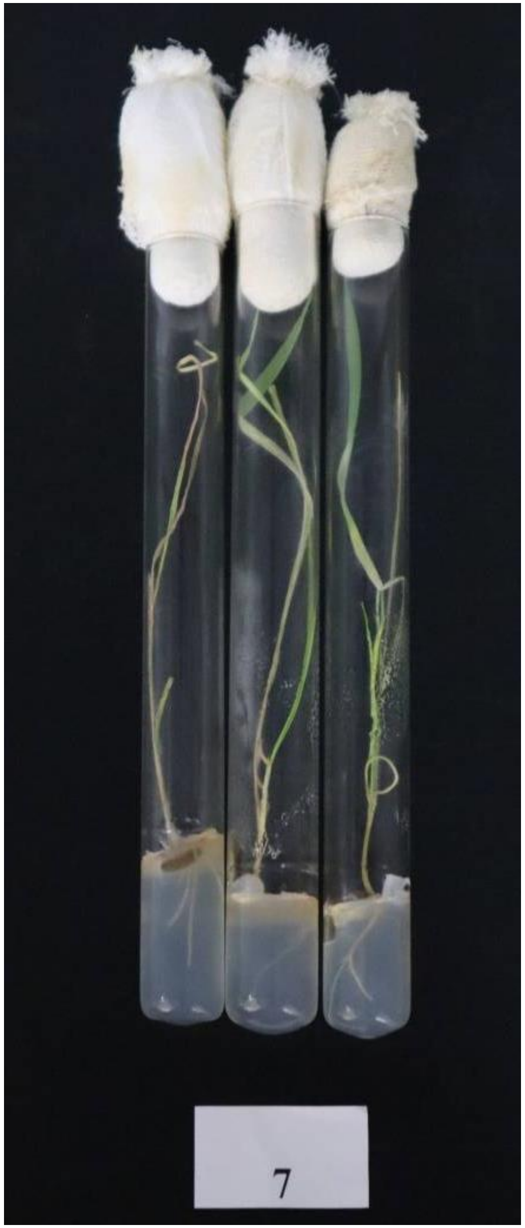

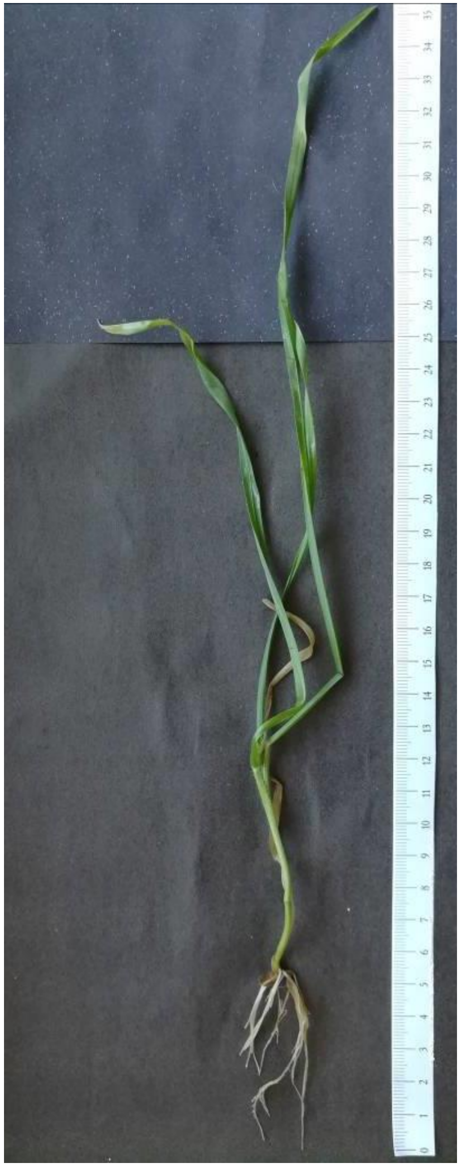

Rye plants infected by *M. nivale* strain 8 (20 dpi)

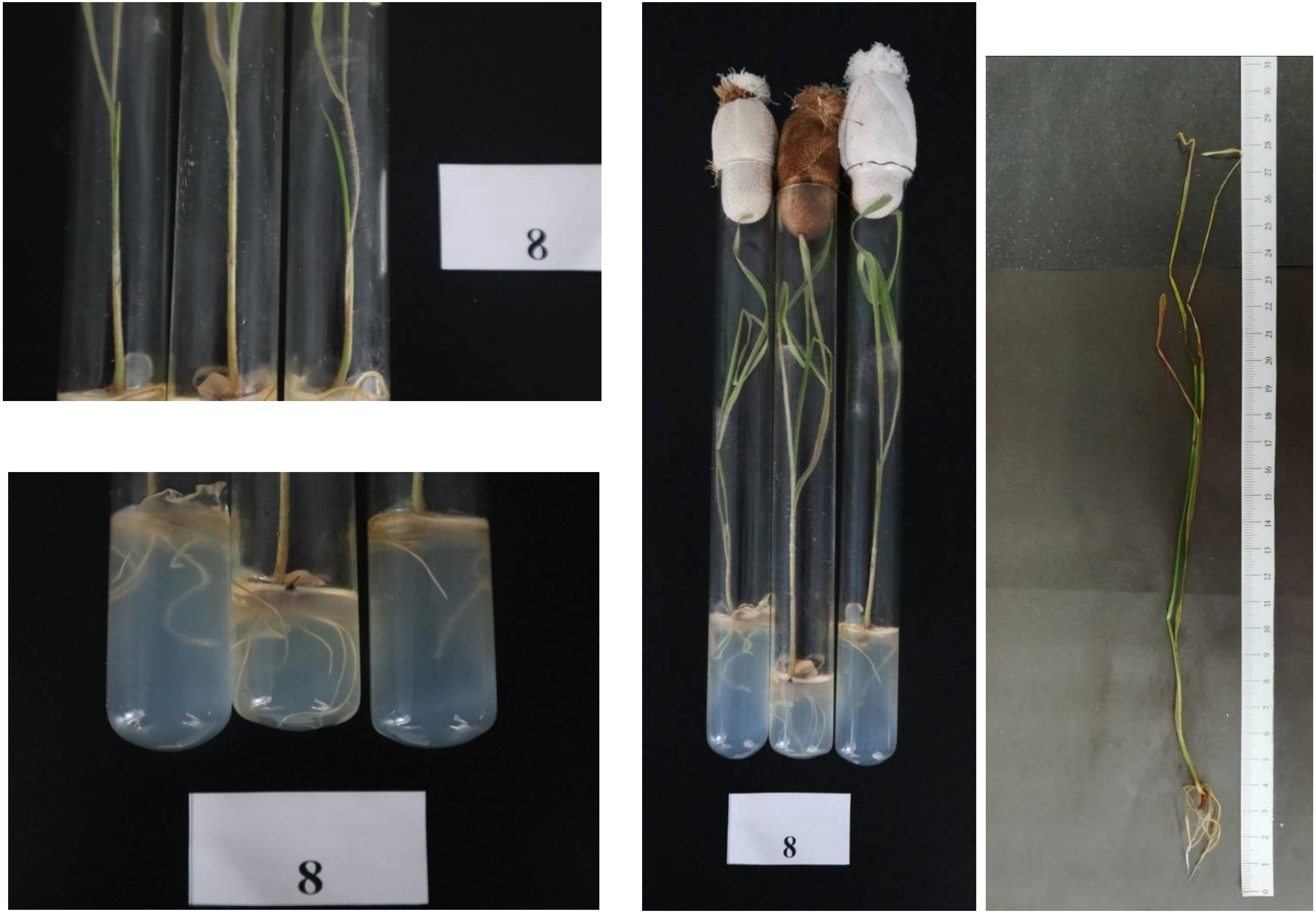

Rye plants infected by *M. nivale* strain 9 (20 dpi)

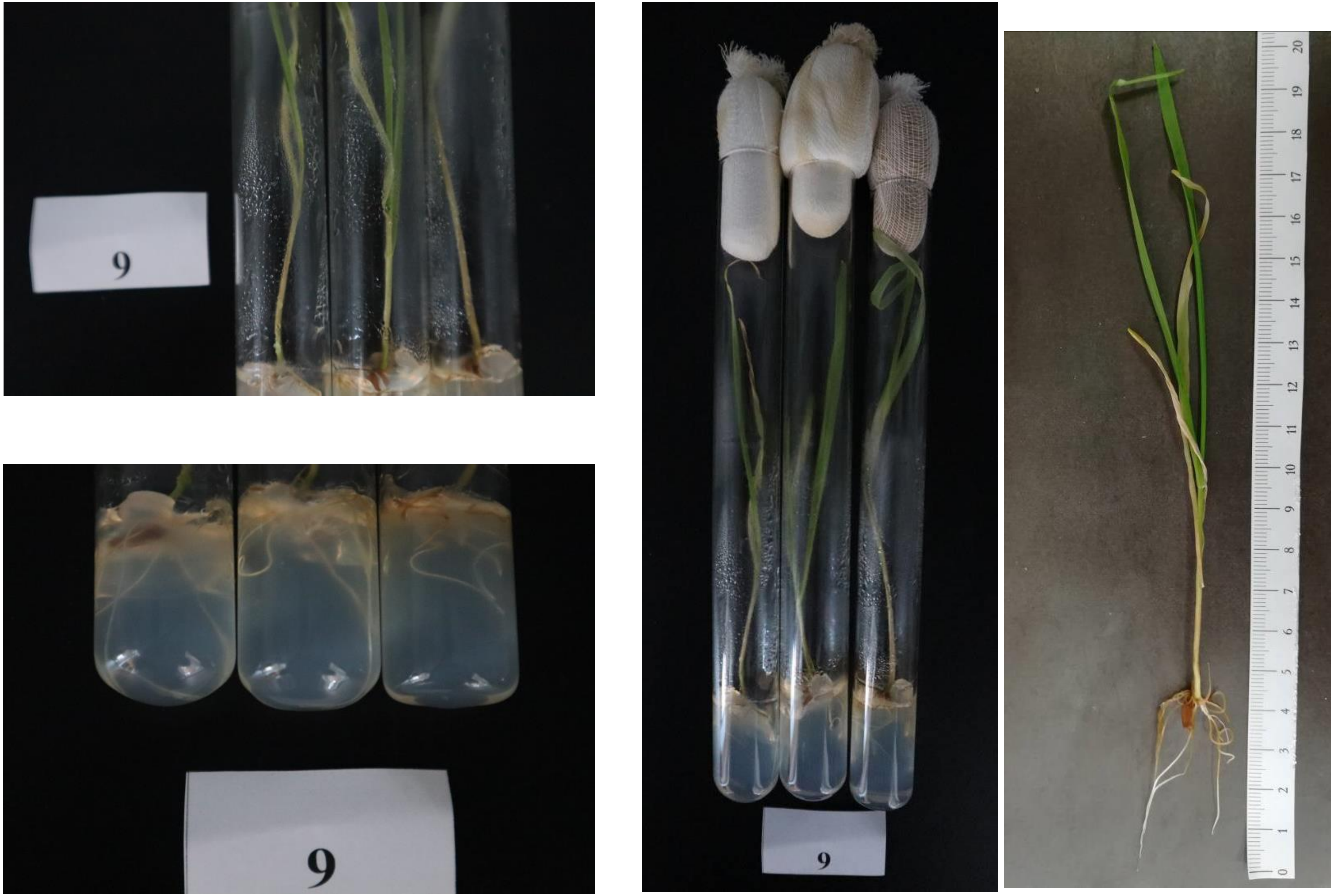

Rye plants infected by *M. nivale* strain 10 (20 dpi)

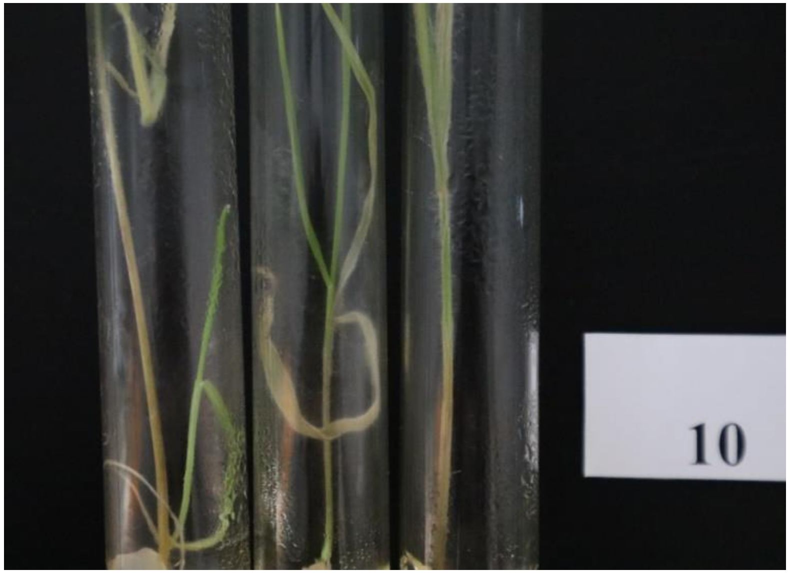

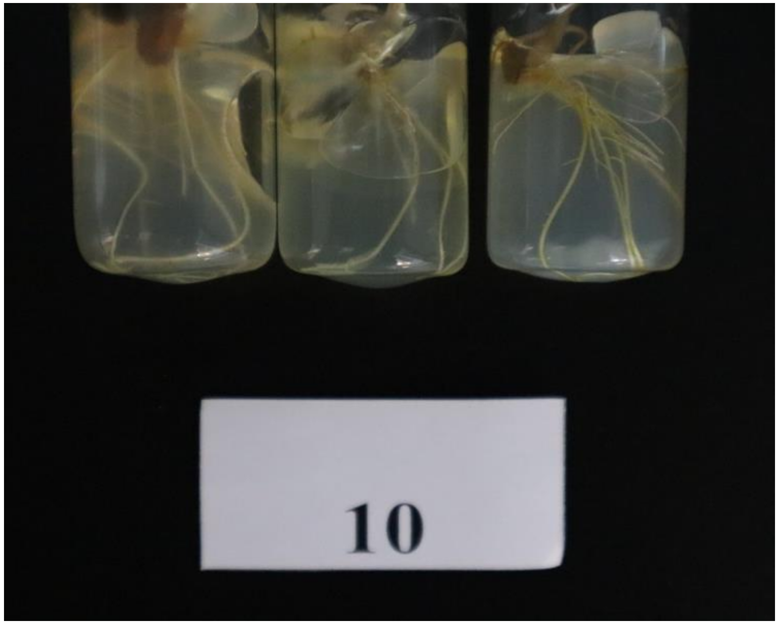

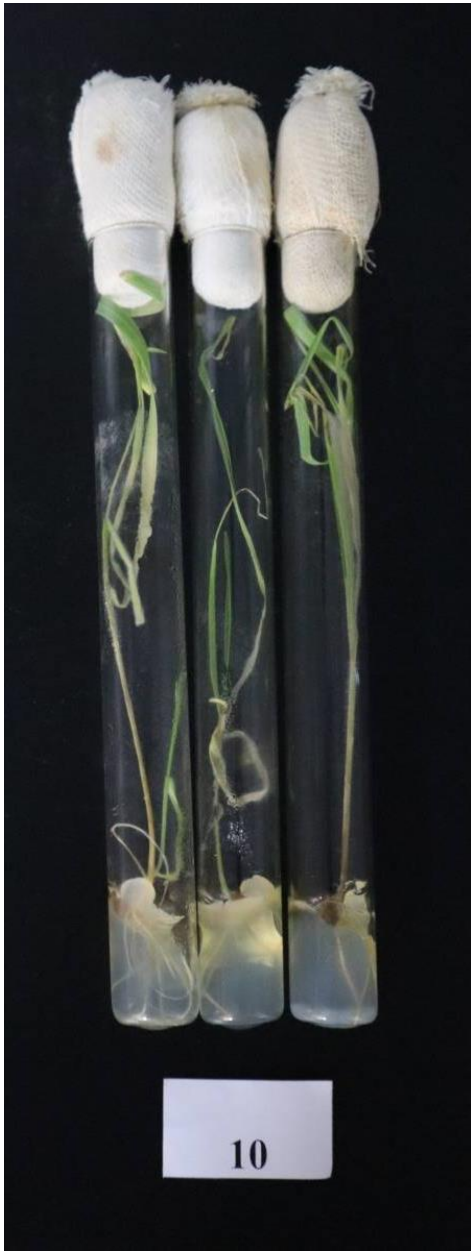

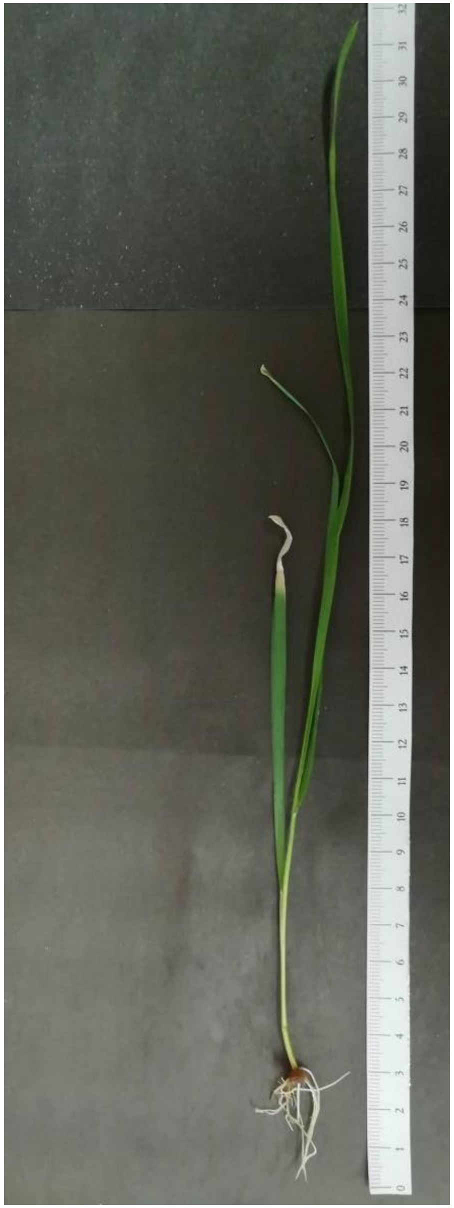

Rye plants infected by *M. nivale* strain 11 (20 dpi)

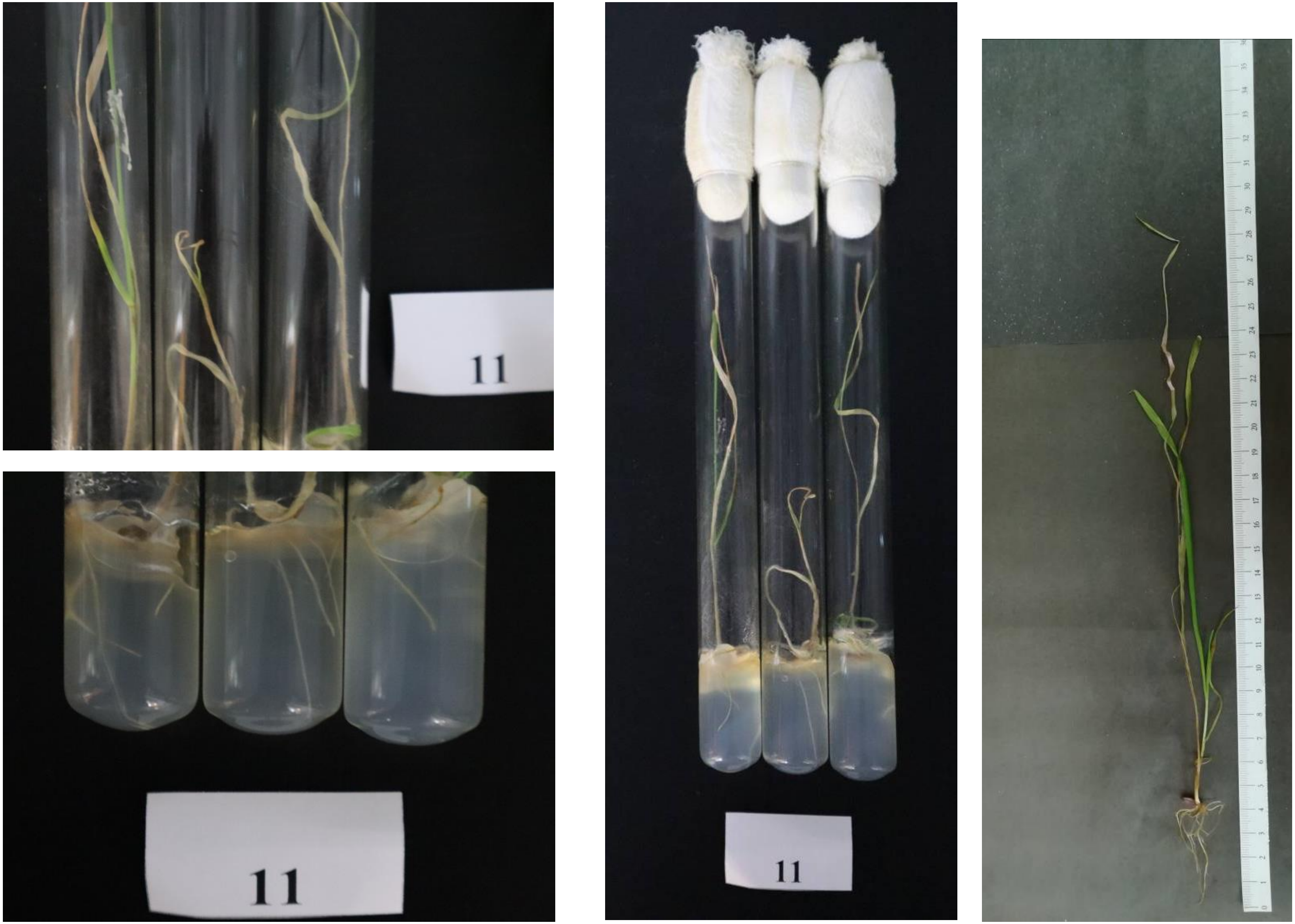

Rye plants infected by *M. nivale* strain 12 (20 dpi)

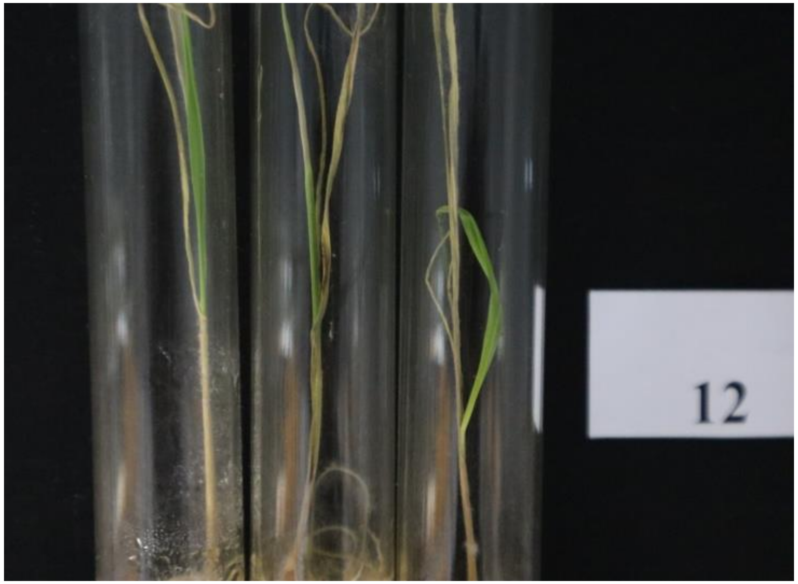

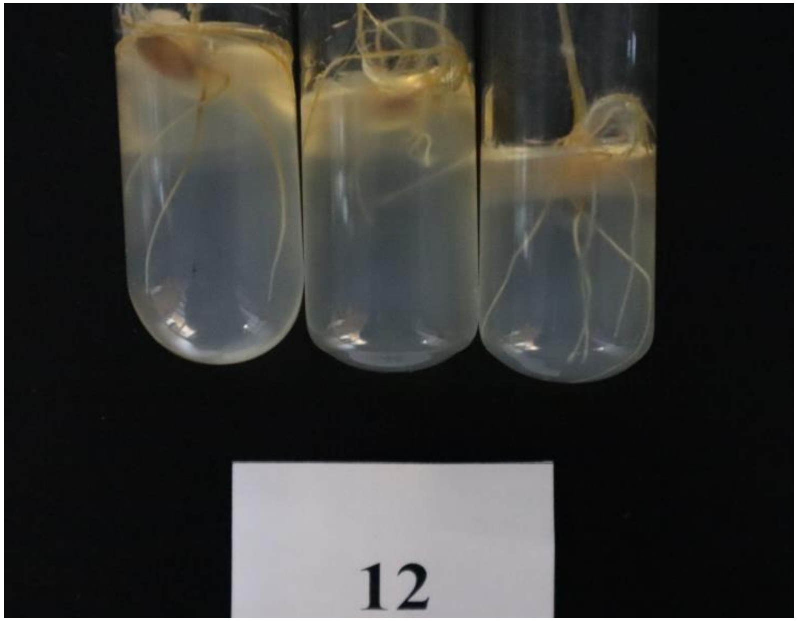

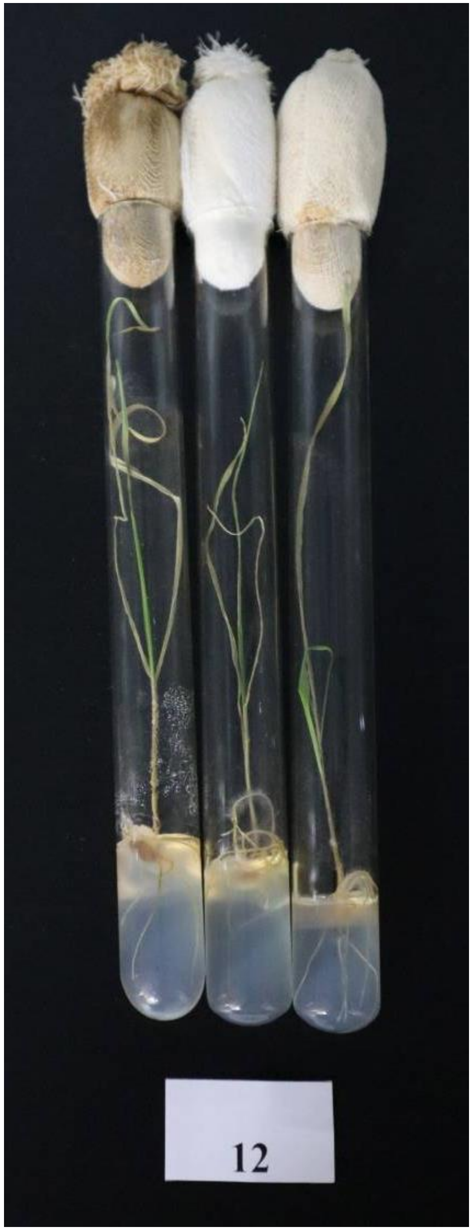

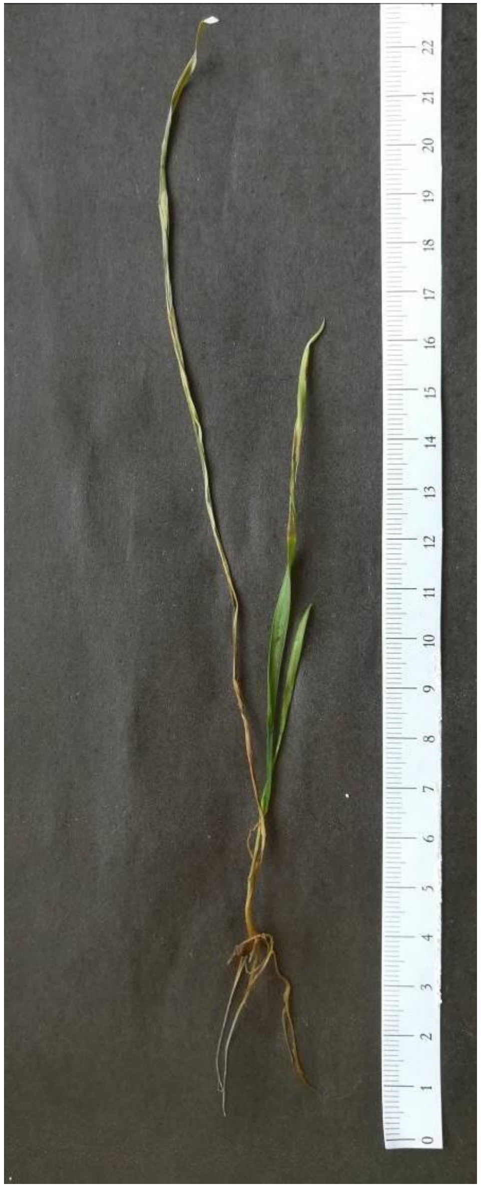

Rye plants infected by *M. nivale* strain13 (20 dpi)

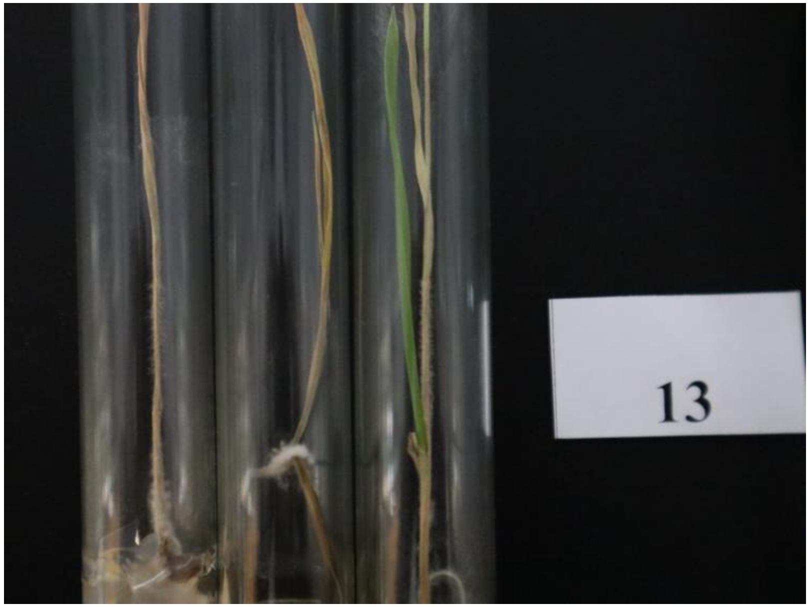

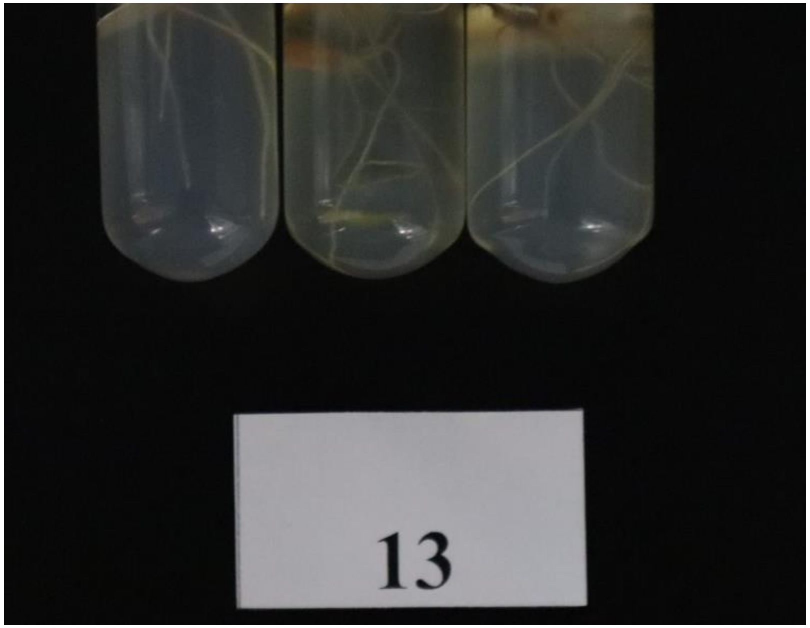

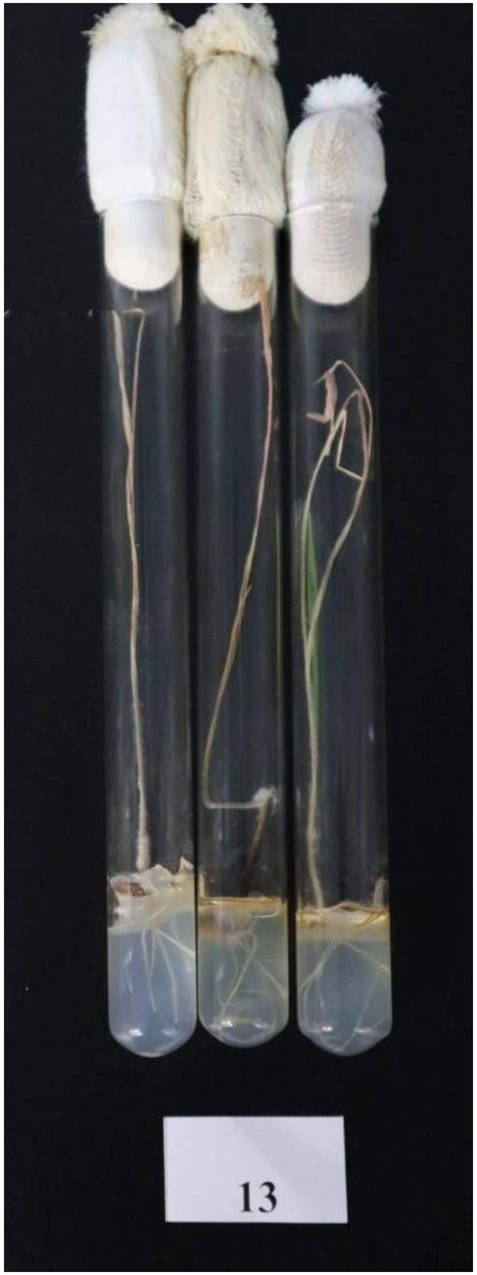

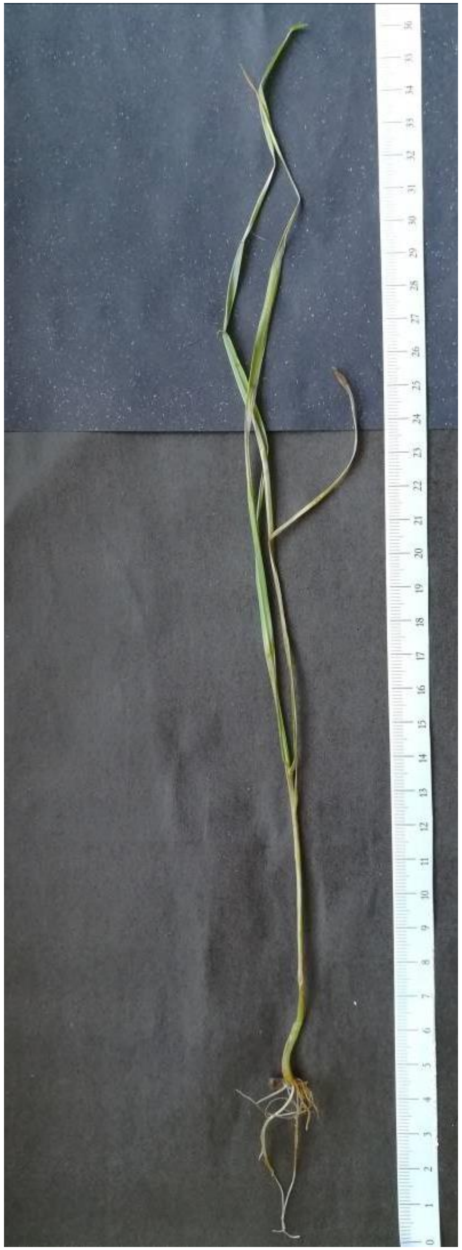

Rye plants infected by *M. nivale* strain 14 (20 dpi)

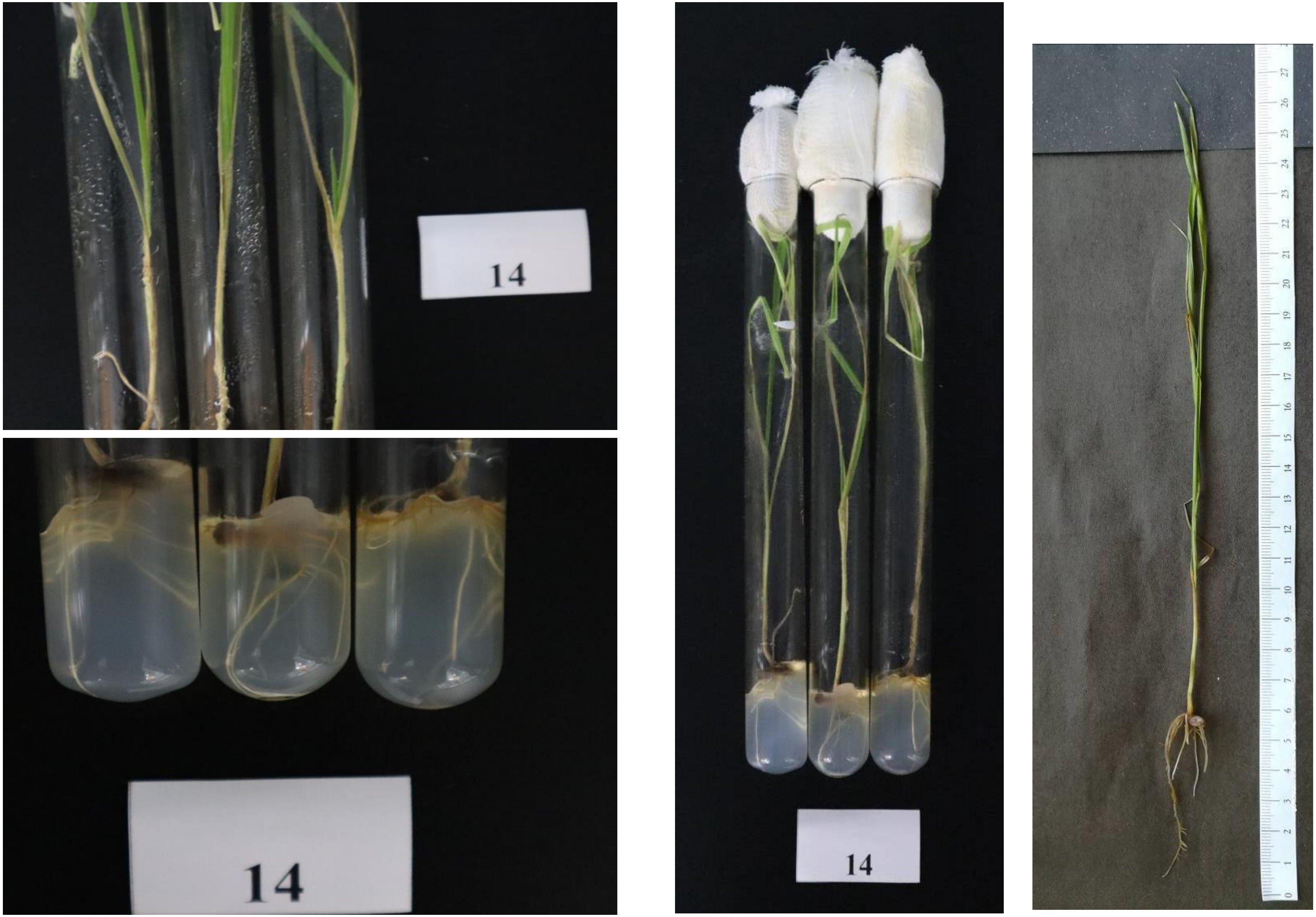

Rye plants infected by *M. nivale* strain 15 (20 dpi)

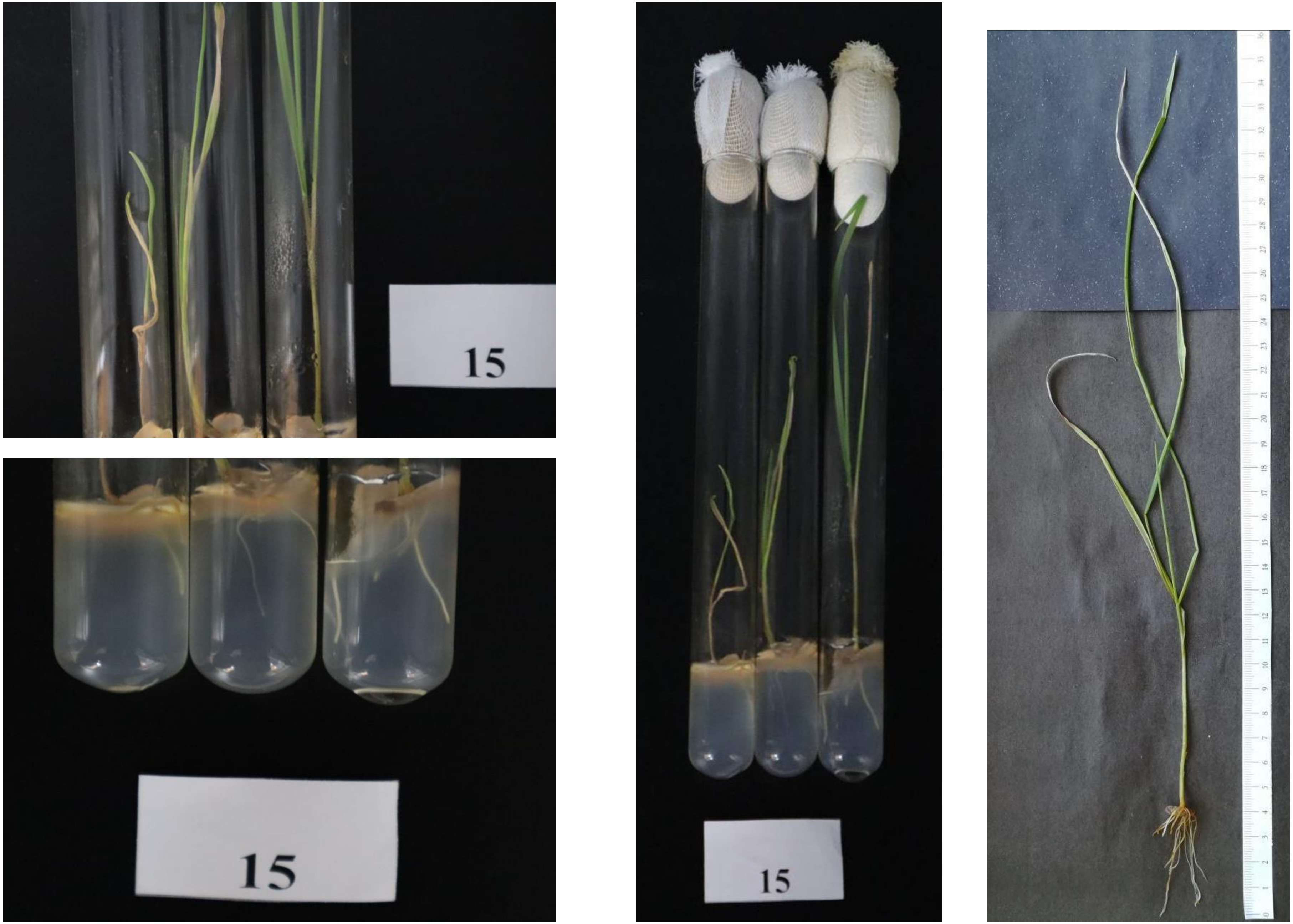

Rye plants infected by *M. nivale* strain 16 (20 dpi)

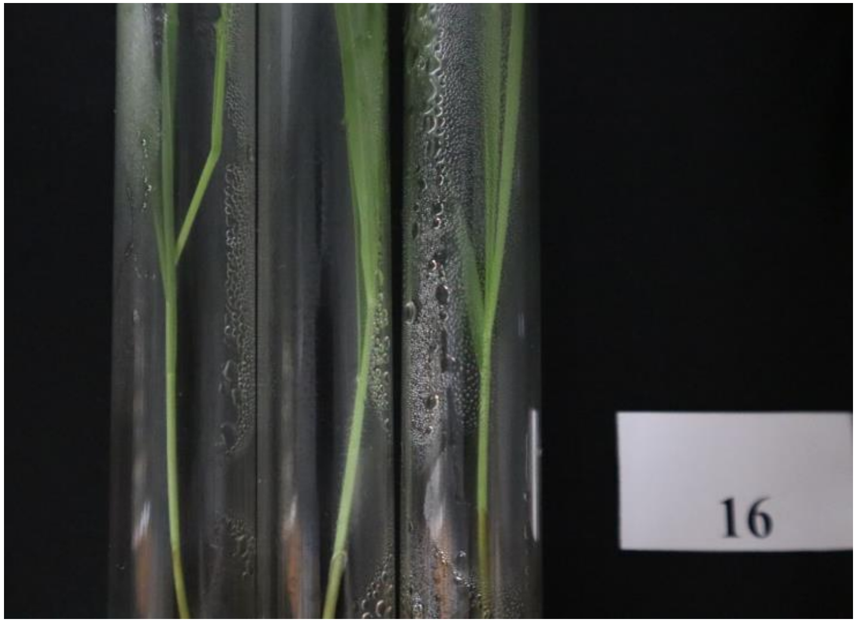

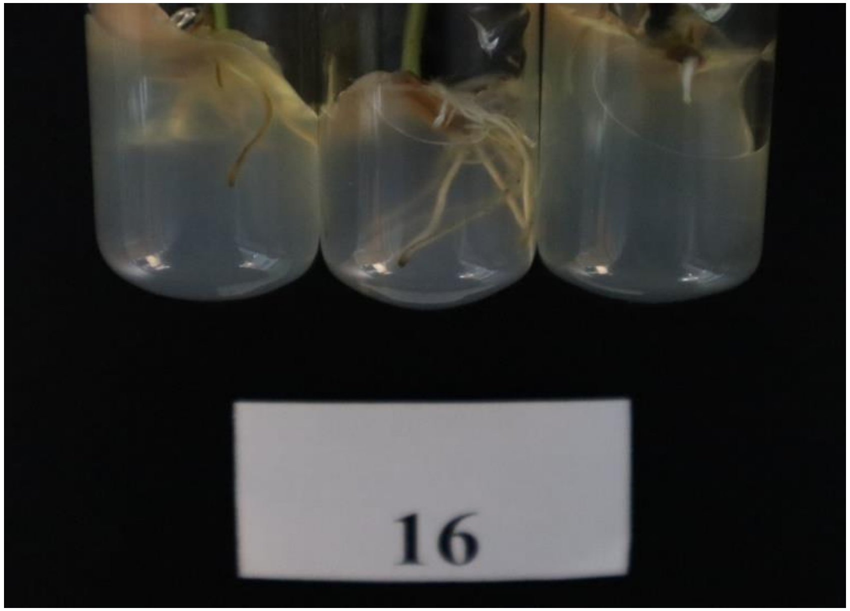

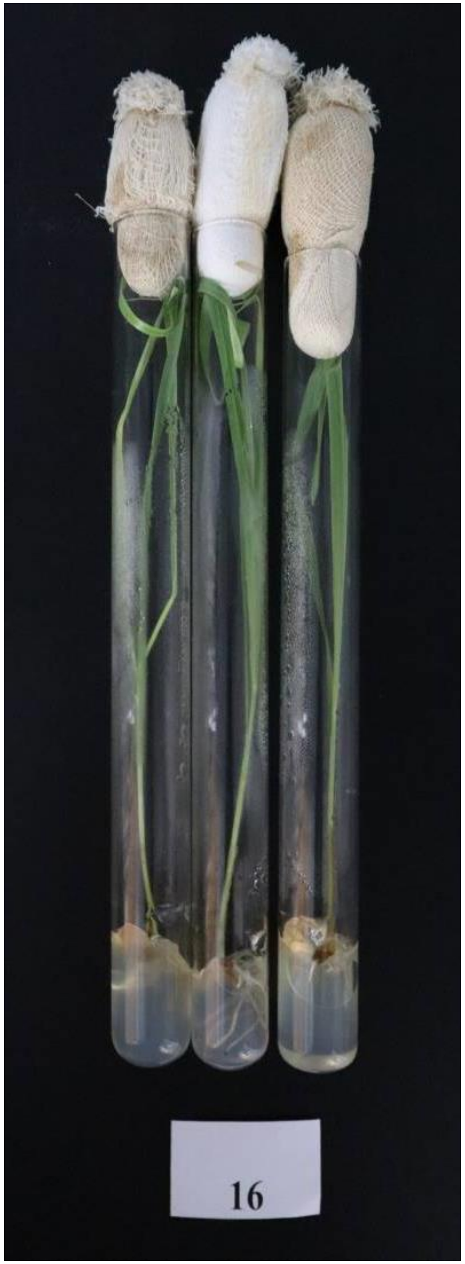

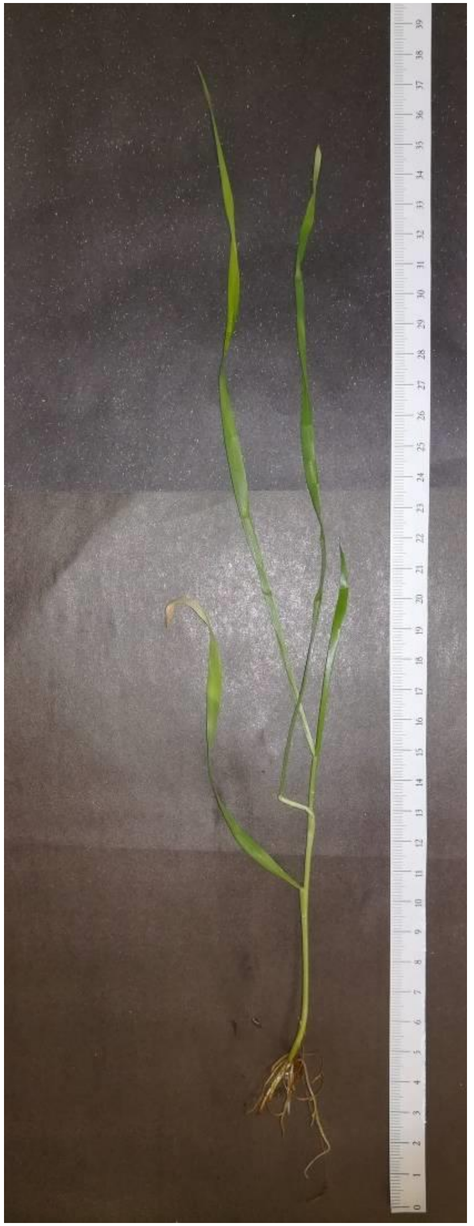

Rye plants infected by *M. nivale* strain 17 (20 dpi)

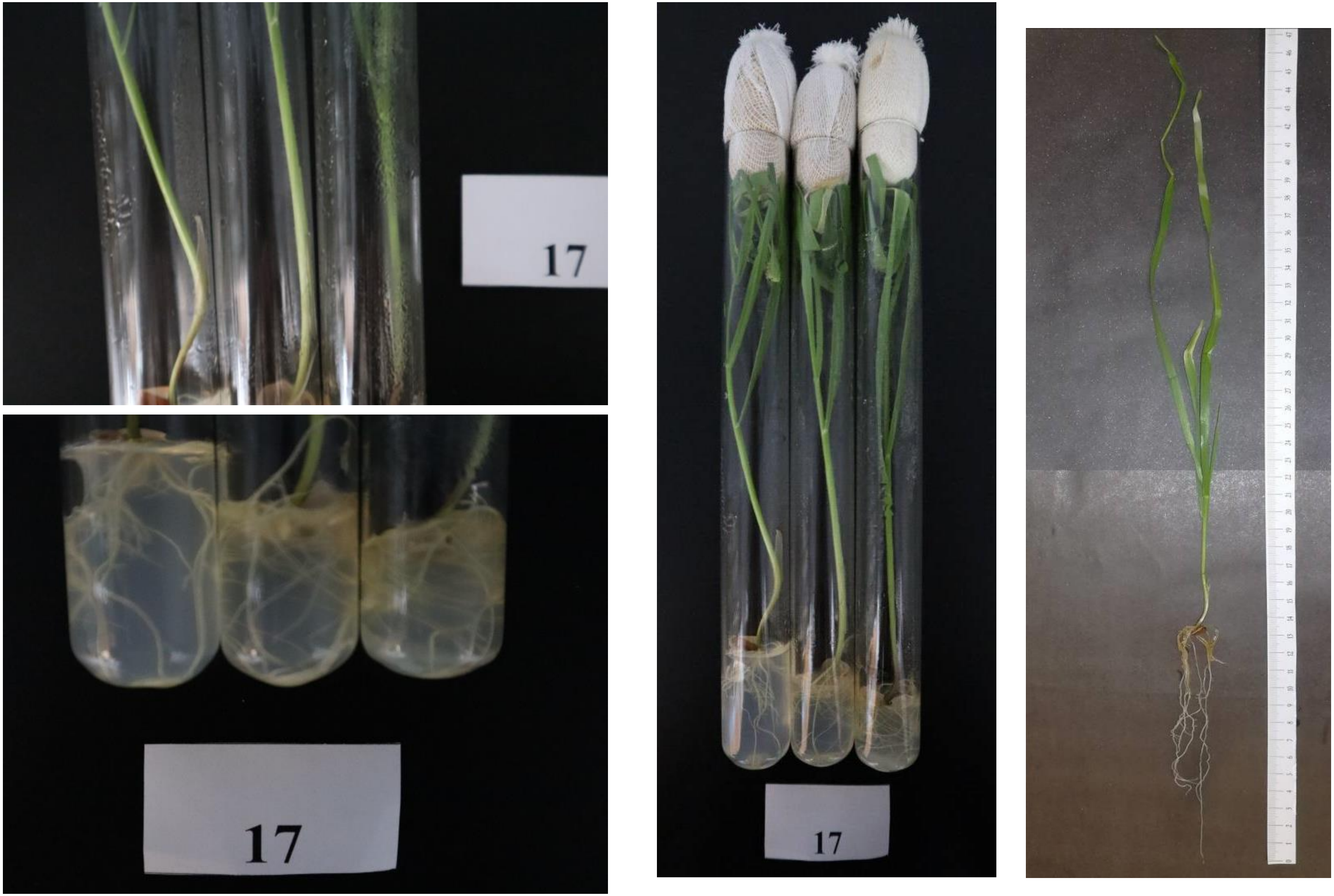

Rye plants infected by *M. nivale* strain 18 (20 dpi)

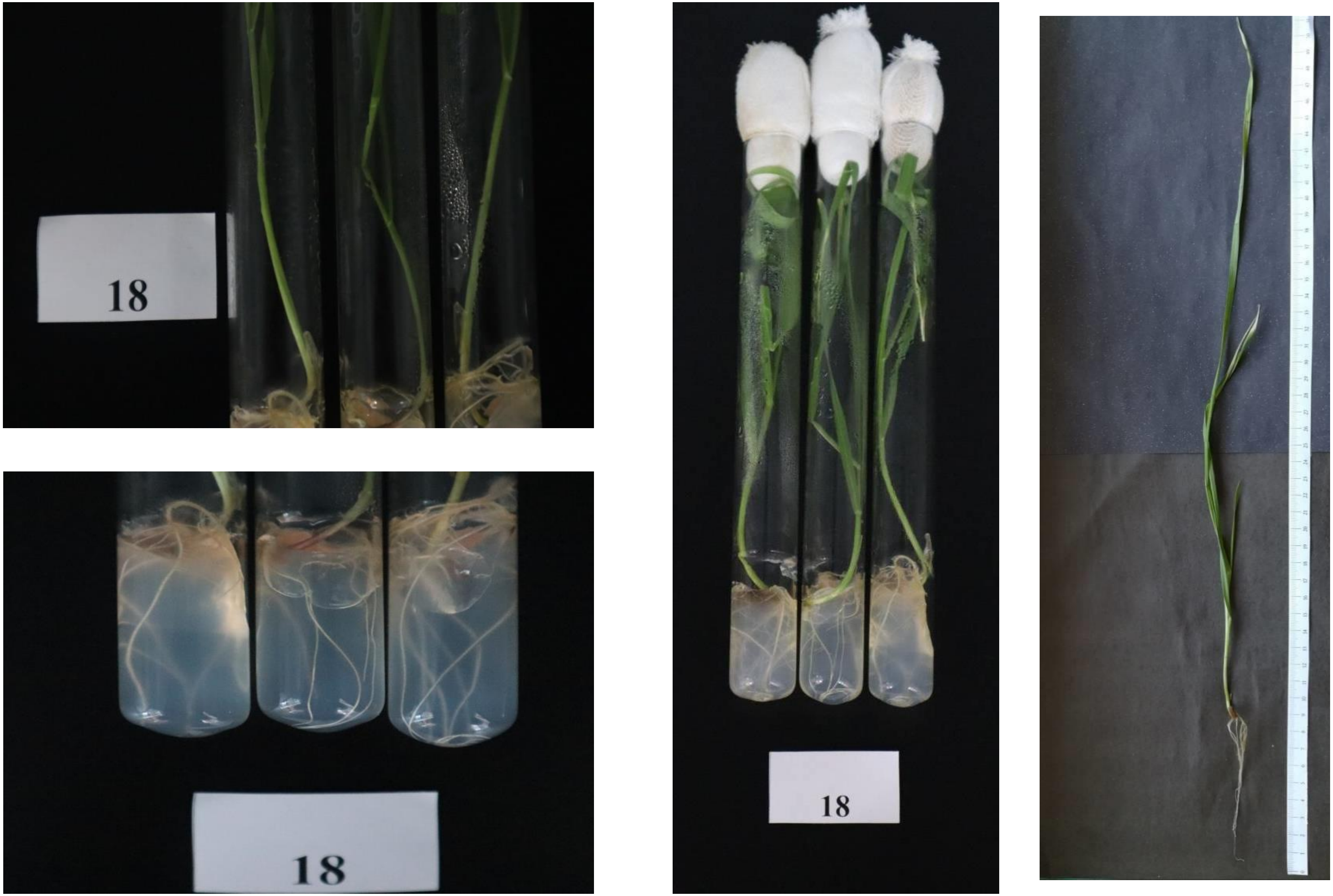

Rye plants infected by *M. nivale* strain 19 (20 dpi)

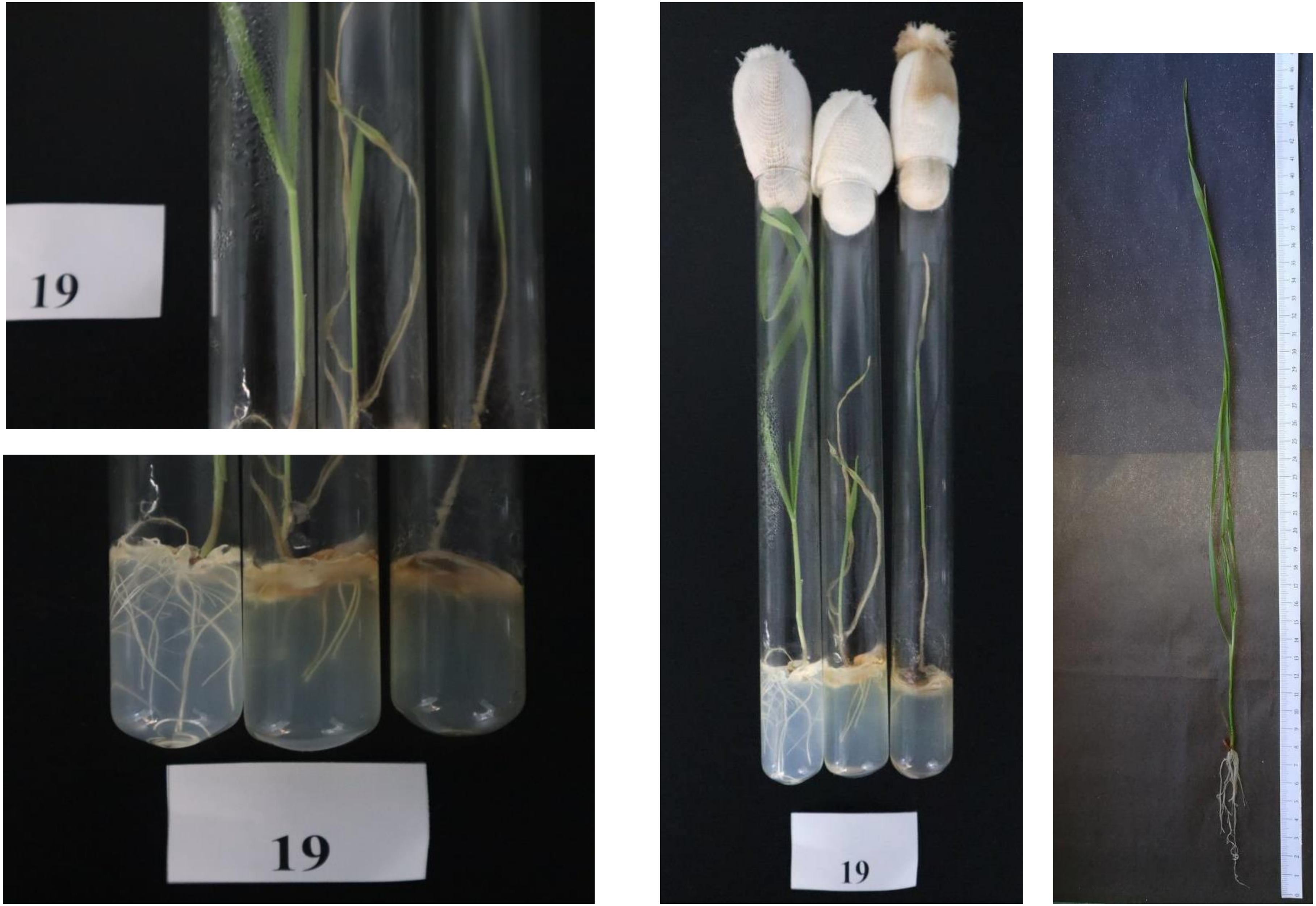

Rye plants infected by *M. nivale* strain 20 (20 dpi)

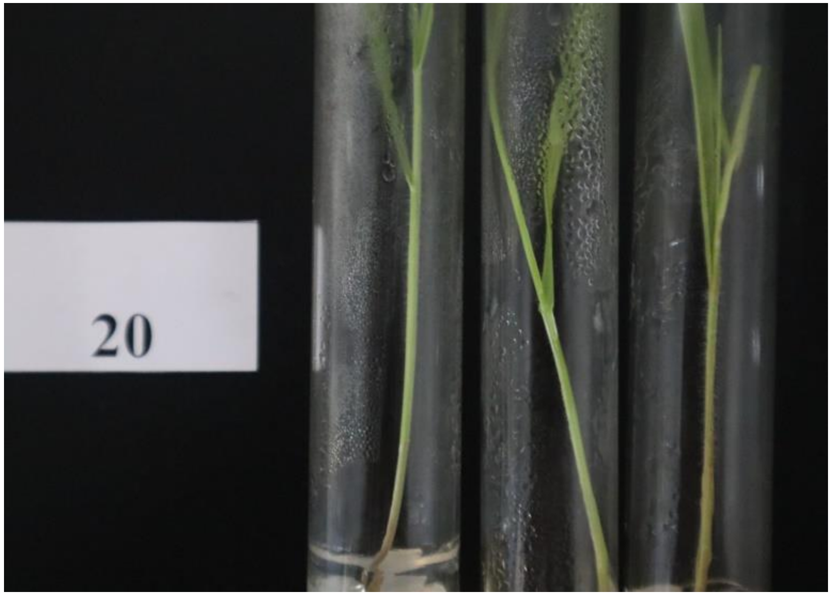

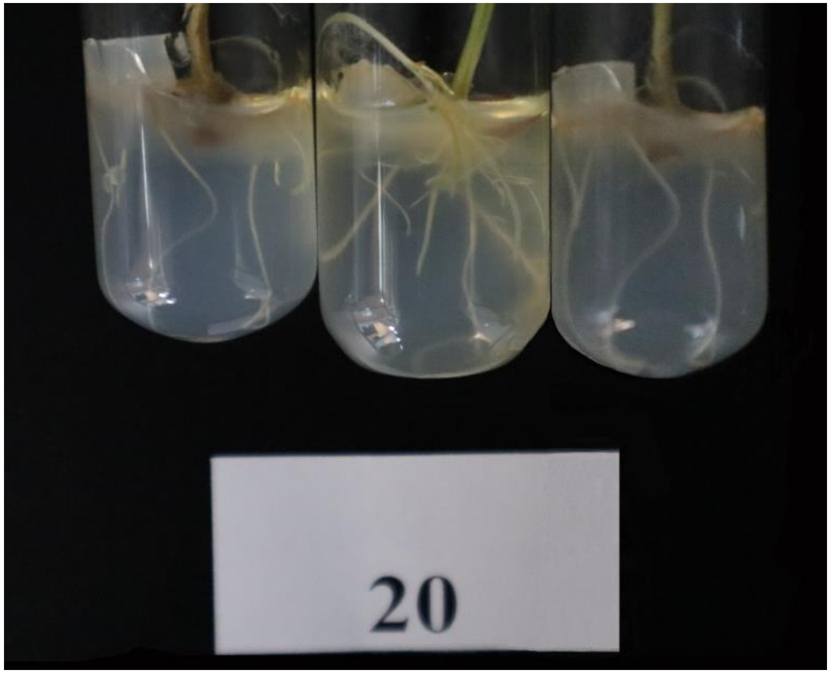

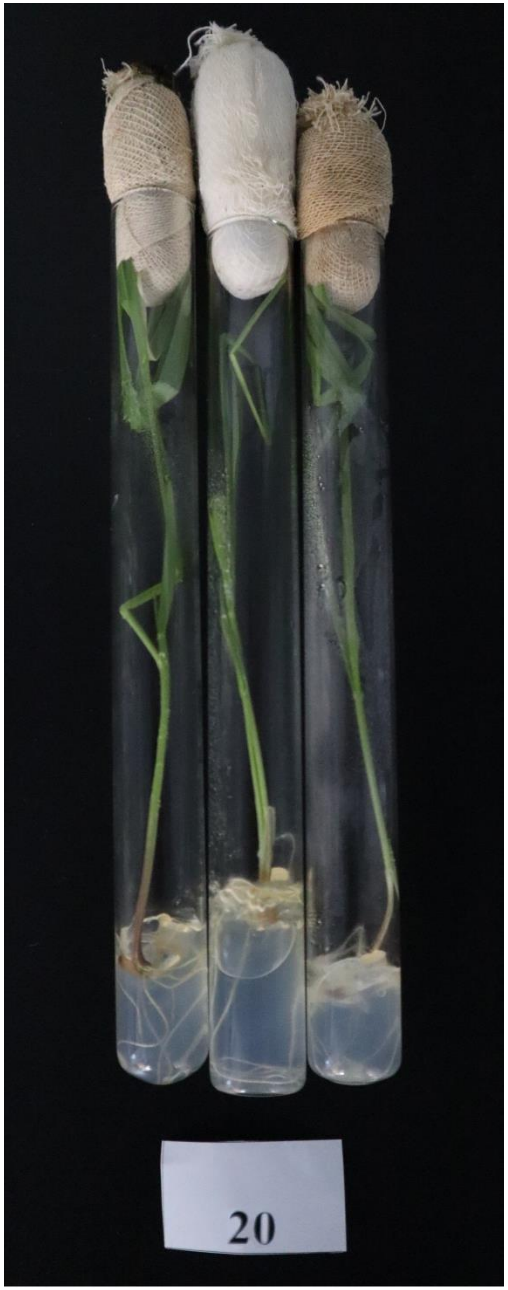

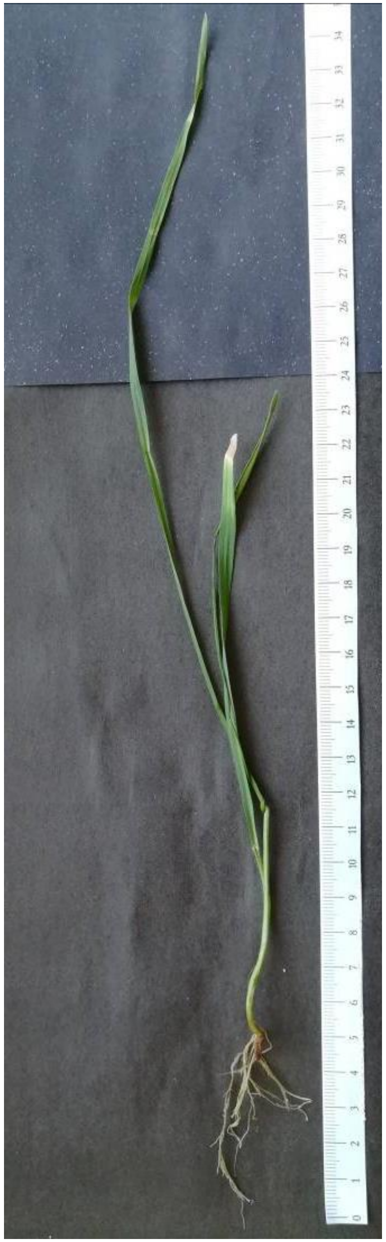

Rye plants infected by *M. nivale* strain 21 (20 dpi)

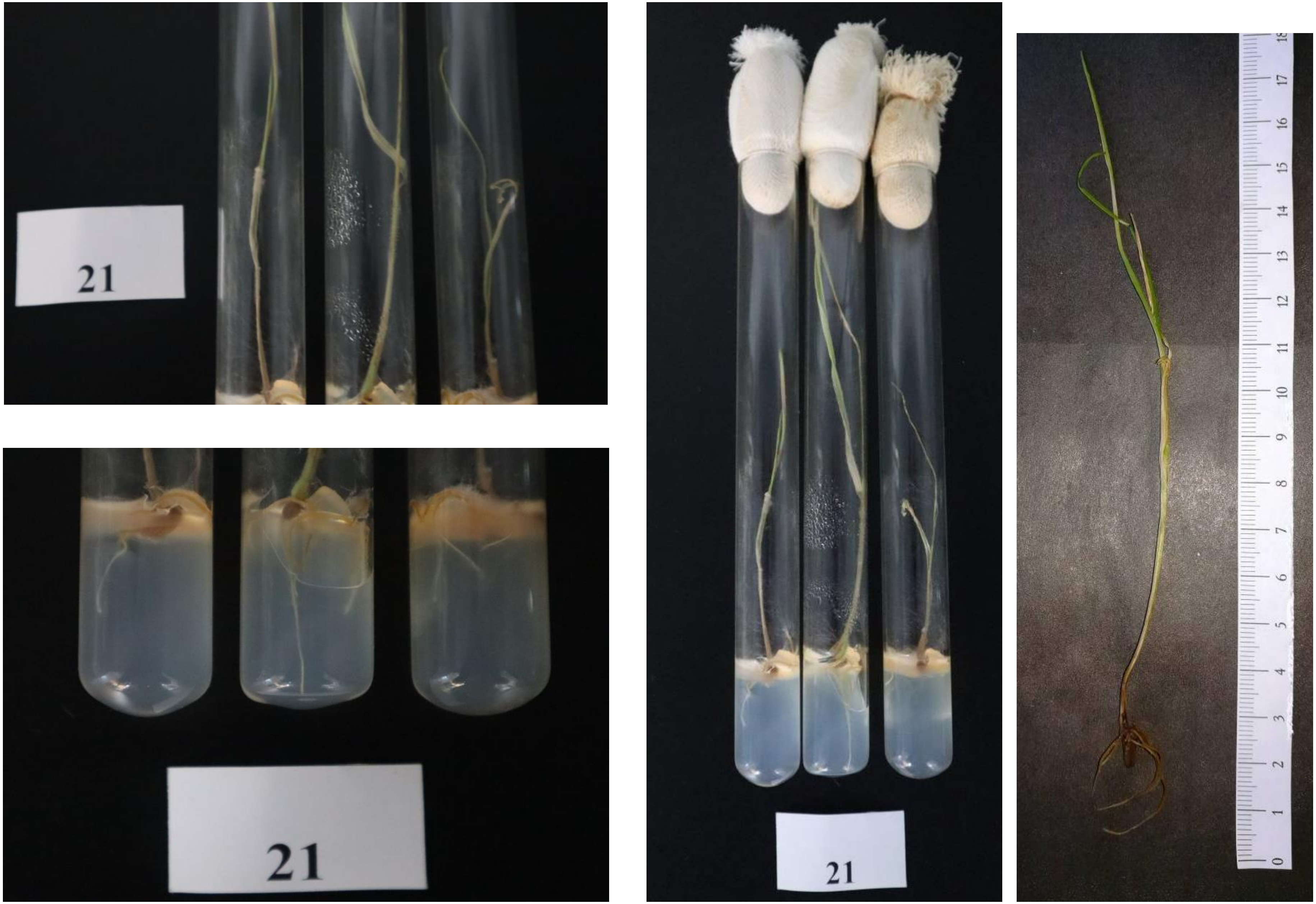

Supplement: Supplementary file 1 [file jof-06-00335-s001.zip › Figure S1.pdf]
