# Supplementary material for: Rye Snow Mold-Associated Microdochium nivale Strains Inhabiting a Common Area: Variability in Genetics, Morphotype, Extracellular Enzymatic Activities, and Virulence
Source: J Fungi (Basel). 2020 Dec 3;6(4):335. doi: 10.3390/jof6040335 (PMC7761817; doi:10.3390/jof6040335)
Supplement: Supplementary file 1 [file jof-06-00335-s001.zip › Table S11.doc]

Table S11. Collection IDs of the isolated strains of *Microdochium nivale*.

| Strain № | Strain ID in the collection |
| --- | --- |
| *Microdochium nivale* 1 | F00608 |
| *Microdochium nivale* 2 | F00609 |
| *Microdochium nivale* 3 | F00610 |
| *Microdochium nivale* 4 | F00611 |
| *Microdochium nivale* 5 | F00612 |
| *Microdochium nivale* 6 | F00613 |
| *Microdochium nivale* 7 | F00614 |
| *Microdochium nivale* 8 | F00615 |
| *Microdochium nivale* 9 | F00616 |
| *Microdochium nivale* 10 | F00617 |
| *Microdochium nivale* 11 | F00618 |
| *Microdochium nivale* 12 | F00619 |
| *Microdochium nivale* 13 | F00620 |
| *Microdochium nivale* 14 | F00621 |
| *Microdochium nivale* 15 | F00622 |
| *Microdochium nivale* 16 | F00623 |
| *Microdochium nivale* 17 | F00624 |
| *Microdochium nivale* 18 | F00625 |
| *Microdochium nivale* 19 | F00626 |
| *Microdochium nivale* 20 | F00627 |
| *Microdochium nivale* 21 | F00628 |
