# Supplementary material for: Rye Snow Mold-Associated Microdochium nivale Strains Inhabiting a Common Area: Variability in Genetics, Morphotype, Extracellular Enzymatic Activities, and Virulence
Source: J Fungi (Basel). 2020 Dec 3;6(4):335. doi: 10.3390/jof6040335 (PMC7761817; doi:10.3390/jof6040335)
Supplement: Supplementary file 1 [file jof-06-00335-s001.zip › Table S12.doc]

Table S12. Morphological characteristics of the isolated *Microdochium nivale* strains after 14 days of cultivation on potato sucrose agar (according to Stalpers, 1978).

| № | Characters of the mat | | | | Exudate |
| --- | --- | --- | --- | --- | --- |
|  | Colony color | Aerial mycelium | Marginal zone | Zonation |  |
| 1 | whitish | pellicular (thin, low, coherent mycelium) | even | concentric | pale yellow-ochraceous |
| 2 | whitish | pellicular (thin, low, coherent mycelium) | even | radial | pale yellow-ochraceous |
| 3 | whitish | pellicular (thin, low, coherent mycelium) | even | concentric | absent |
| 4 | whitish | farinaceous (mealy, powdery) | even | concentric | pale yellow-ochraceous |
| 5 | whitish | pellicular (thin, low, coherent mycelium), asymmetric | even | not pronounced | pale yellow-ochraceous |
| 6 | whitish | farinaceous (mealy, powdery) | even | low, concentric and radial | pale yellow-ochraceous |
| 7 | whitish | absent (mycelium only submerged) | bayed | not pronounced | pale yellow-ochraceous |
| 8 | whitish | felty (cottony or woolly mycelium, which has become matted or packed, emerging hyphae absent), asymmetric | uneven | not pronounced | pale yellow-ochraceous |
| 9 | whitish | pellicular (thin, low, coherent mycelium), asymmetric | even | concentric, radial | pale yellow-ochraceous |
| 10 | whitish | pellicular (thin, low, coherent mycelium), asymmetric | even | low radial and concentric | pale yellow-ochraceous |
| 11 | whitish | pellicular (thin, low, coherent mycelium), asymmetric | even | radial, with solid segments, concentric | pale yellow-ochraceous |
| 12 | whitish | floccose (small hyphal tufts, standing out from the agar or from the aerial mycelium) | even | low concentric | pale yellow-ochraceous |
| 13 | whitish | absent (mycelium only submerged, with solid hyphal bundles) | even | low concentric | pale yellow-ochraceous |
| 14 | whitish | cottony (rather long, single mycelial hyphae spreading in all directions, submerged mycelium forms solid hyphal bundles), asymmetric | uneven, bayed | concentric | pale yellow-ochraceous |
| 15 | whitish | felty (cottony or woolly mycelium, which has become matted or packed, emerging hyphae absent), asymmetric | bayed | concentric | pale yellow-ochraceous |
| 16 | whitish | floccose (small hyphal tufts, standing out from the agar or from the aerial mycelium), asymmetric | even | low concentric | pale yellow-ochraceous |
| 17 | light pink | pellicular (thin, low, coherent mycelium), asymmetric | even | radial | pale yellow-ochraceous |
| 18 | light pink | farinaceous (mealy, powdery) | even | concentric | pale yellow-ochraceous |
| 19 | from whitish to pink | floccose (small hyphal tufts, standing out from the agar or from the aerial mycelium), asymmetric | even | radial, concentric | pale yellow-ochraceous |
| 20 | from whitish to creamy | pellicular (thin, low, coherent mycelium) | even | radial | absent |
| 21 | whitish | silky (long, radiating parallel hyphae or hyphal bundles, more or less prostrate) | even | radial, concentric | absent |
