# Supplementary material for: Rye Snow Mold-Associated Microdochium nivale Strains Inhabiting a Common Area: Variability in Genetics, Morphotype, Extracellular Enzymatic Activities, and Virulence
Source: J Fungi (Basel). 2020 Dec 3;6(4):335. doi: 10.3390/jof6040335 (PMC7761817; doi:10.3390/jof6040335)
Supplement: Supplementary file 1 [file jof-06-00335-s001.zip › Table S13.doc]

Table S13. Morphology of the conidia of the isolated *Microdochium nivale* strains.

| Strain No | 1 | 3 | 5 | 10 | 14 | 17 | 19 |
| --- | --- | --- | --- | --- | --- | --- | --- |
| Sizes (µm) | 15.5-19.6 × 2.8-3.7 | 14-18.2 × 2.4-3.3 | 13.1-19.9 × 2.7-4.1 | 13.0-18.3 × 2.4-3.6 | 12.7-18.9 × 2.2-3.3 | 13.2-20.3 × 1.8-2.8 | 10.1-18.1 × 2.1-2.9 |
| Number of septa | 1-3 | 1 | 1-3 | 0-2 | 1-3 | 0-1 | 0-2 |
